# Demystifying anti-inflammatory therapeutic strategies against pancreatitis and concomitant diseases: a 2025 perspective

**DOI:** 10.7150/thno.127402

**Published:** 2026-01-01

**Authors:** Mengli Yue, Sibtain Muhammad, Song Jiang, Madappa C Maridevaru, Yinghe Zhang, Bing Guo, Pi Liu

**Affiliations:** 1 Department of Gastroenterology, People's Hospital of Longhua Shenzhen, Shenzhen School of Clinical Medicine, Southern Medical University, China.; 2 School of Science, Shenzhen Key Laboratory of Advanced Functional Carbon Materials Research and Comprehensive Application, Harbin Institute of Technology, Shenzhen, 518055, China.; 3 Department of Gastroenterology, People's Hospital of Longhua, Shenzhen, 518109, China.

**Keywords:** oxidative stress, nanomedicine, anti-inflammation, Traditional Chinese Medicine, gas therapy, gene therapy

## Abstract

Pancreatitis is a complex, progressive inflammatory disorder that often transitions from acute to chronic stages, leading to significant comorbidities such as diabetes, cardiovascular diseases, and bone disorders. These complications complicate treatment strategies, highlighting the need for more targeted approaches. Historically, therapeutic strategies have focused on mechanisms addressing immune dysregulation, oxidative stress, and tissue damage. However, recent advancements have focused on anti-inflammatory mechanisms as primary therapeutic targets for pancreatitis and its associated conditions, as anti-inflammatory treatments help in alleviating chronic inflammation, minimizing tissue damage, and inhibiting disease progression, thereby enhancing recovery and overall patient well-being. This review highlights the most recent advancements in anti-inflammatory therapies for pancreatitis and associated diseases. We discuss how nanotechnology, particularly synthetic and biomimetic nanocarrier-based systems, has emerged as a promising approach to enhance the targeted delivery of anti-inflammatory agents, improving bioavailability and reducing systemic side effects. Importantly, we highlight the potential of gas-based therapies, traditional Chinese medicines, gene therapy, specialty food, living probiotics, exosomes, and even multitargeted approaches to enhance the therapeutic efficacy for pancreatitis and concomitant diseases. Despite these advancements, challenges such as treatment stability, immune response modulation, and scalability have been discussed. Moreover, perspective therapies such as single cell therapy and early diagnosis as treatments have been discussed. This review underscores the need for personalized, multimodal strategies to improve outcomes in pancreatitis management.

## 1. Introduction

**Scheme 1:**
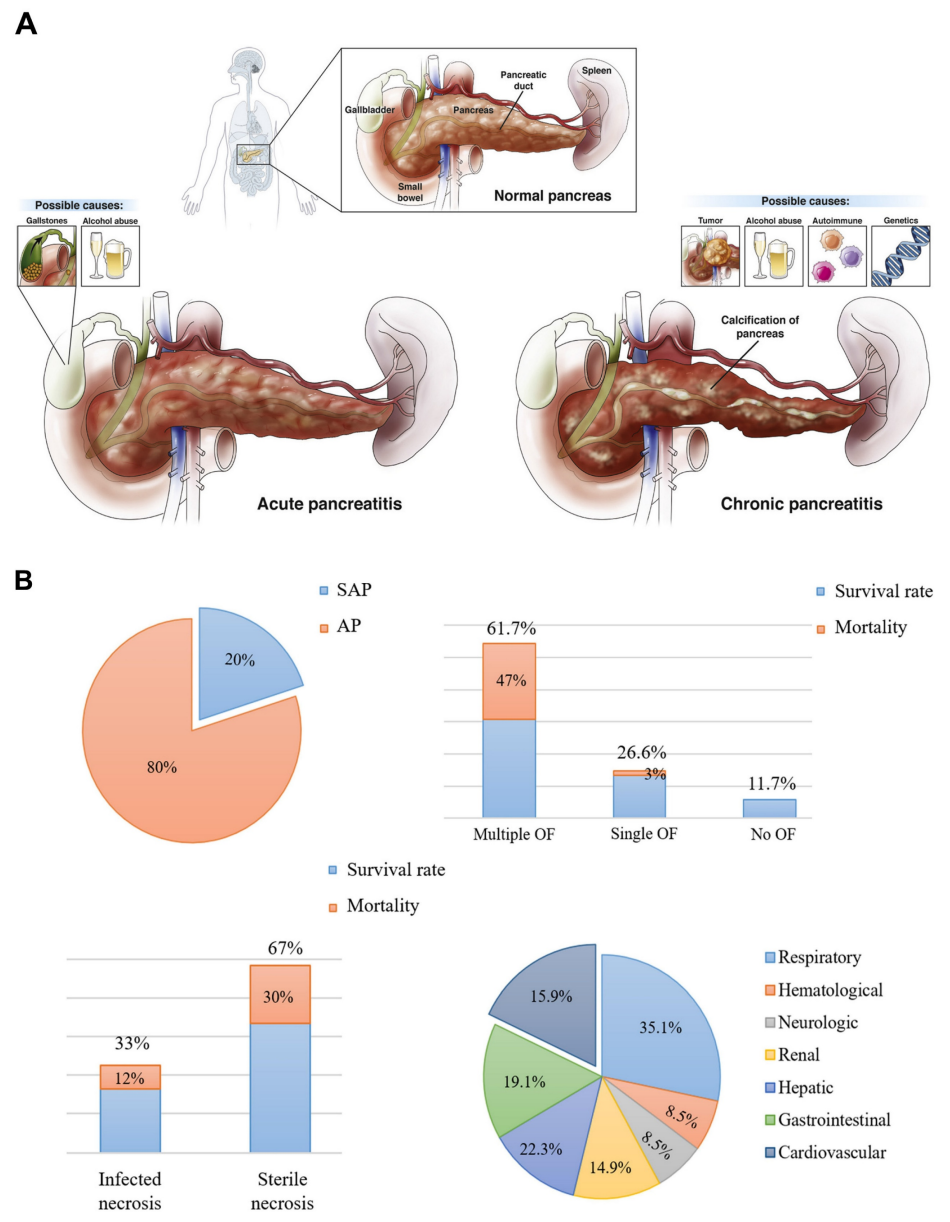
Pancreatitis**. (A)** Illustration of different types of pancreatitis. **(B)** Data on acute pancreatitis including the proportion and fatality rates in AP of varying severity, as well as the proportion and mortality rates in AP exacerbated by organ failure. Adapted with permission from ref. 29 Copyrights 2025, Elsevier.

Pancreatitis is a pathological condition primarily triggered by the premature activation of digestive enzymes, particularly trypsinogen, within the pancreas, leading to autodigestion of pancreatic tissues 1. The condition is generally categorized into two forms: acute pancreatitis (AP) and chronic pancreatitis (CP), which exhibit varying levels of inflammation, hemorrhage, and tissue necrosis that primarily affect the acinar cells and surrounding pancreatic tissues (**Scheme 1A**) 2. While significant progress has been made in understanding the pathophysiology of AP, many aspects remain unclear 3. The condition is associated with numerous etiological factors, such as gallstone-induced biliary obstruction, excessive alcohol consumption, adverse drug reactions, infections, hypercalcemia, genetic predispositions, and autoimmune conditions 4. In AP, the inappropriate activation of trypsinogen leads to a cascade of enzymatic reactions that cause widespread tissue damage and self-digestion. In cases of mild acute pancreatitis (MAP), the inflammation is typically localized, and organ function is largely preserved. However, severe acute pancreatitis (SAP) involves more widespread inflammation, leading to extensive tissue necrosis, organ failure, and septic complications 5. Despite advances in intensive care, the mortality rate for SAP remains high, hovering around 30% 6. Over time, repeated or unresolved inflammation may progress to CP, which is characterized by the replacement of functional pancreatic tissue with fibrotic scar tissue 7. This fibrotic remodeling leads to the atrophy of ducts and glands, along with calcification and obstruction of the pancreatic ducts 8. Studies suggest that approximately 20% of AP cases may eventually evolve into CP 9. CP is marked by persistent inflammation, irreversible fibrosis, and gradual loss of both exocrine and endocrine function 10. Early detection of CP remains a challenge due to the lack of specific biomarkers, making diagnosis reliant on clinical evaluation, imaging studies, and functional tests 11. Diagnosis currently relies on a combination of clinical assessment, imaging studies, and pancreatic function evaluations 12. To date, no disease-modifying therapy has been conclusively established for CP. As a result, treatment is largely palliative, aimed at improving patient quality of life by managing symptoms 13. This includes lifestyle modifications such as abstaining from alcohol and smoking, use of analgesics including both non-opioid and opioid regimens and surgical interventions in cases with anatomical abnormalities or ductal obstruction 14.

AP is a prevalent gastrointestinal disorder that often necessitates hospital admission. Over recent decades, its global incidence has steadily increased by approximately 3% annually 15. This upward trend is closely associated with several contributing factors, including excessive alcohol intake, gallstone disease, obesity, and aging populations 16. Clinically, AP presents along a spectrum of severity ranging from mild and self-limiting to life-threatening forms and is categorized as mild, moderately severe, or severe based on clinical progression and organ involvement 17. Radiologically, AP is typically classified into two distinct types: acute interstitial pancreatitis and necrotizing pancreatitis 18. Each exhibit specific morphological patterns and requires different therapeutic strategies 19. The course and severity of AP are influenced by a complex interplay of genetic factors, environmental exposures, and immune system dynamics (**Scheme 1B**) 20. Emerging evidence highlights the significant role of the gut microbiota in shaping immune responses and regulating inflammation throughout the course of AP. Alterations in microbial composition may contribute to disease severity and outcomes, suggesting a potential avenue for therapeutic intervention 21. This evolving understanding underscores the multifactorial nature of AP and the importance of integrated approaches in its diagnosis, management, and treatment development. Lovett *et al*. 22 was the first to report in 1971 that AP could lead to cardiac complications, specifically myocardial dysfunction. This condition is marked by impaired myocardial contractility, reduced ejection fraction, diminished responsiveness to fluid resuscitation, a lower peak systolic pressure-to-end-systolic volume ratio, and cardiac enlargement. Subsequent studies investigating systemic organ failure in SAP revealed that approximately 26.6% of patients developed failure in a single organ system. Among these, the respiratory system was most commonly affected (35.1%), followed by the gastrointestinal tract (19.1%), cardiovascular system (22.3%), liver (15.9%), kidneys (14.9%), and both hematological and neurological systems (8.5%). Notably, multiple organ failure (MOF) was observed in 61.7% of cases 23.

Additional research has shown that SAP may be responsible for up to 60.5% of myocardial infarctions (MIs) 24. Affected individuals frequently present with coexisting cardiac symptoms such as myocarditis, disrupted cardiac output, troponin elevation, arrhythmias, cardiogenic shock, and different MI subtypes 25. Despite improvements in diagnostics, therapeutic techniques, and imaging, the global burden of AP continues to grow. For instance, AP incidence in the UK has increased substantially from 6.9 to 75 per 100,000 in men and from 11.2 to 48 per 100,000 in women 26. In the U.S., rates have reached roughly 45 per 100,000 27. Managing cardiac injury in the context of AP remains clinically challenging and significantly impacts patient survival. Therefore, reducing mortality hinges on an in-depth, systematic understanding of the mechanisms driving SAP-associated cardiac injury and the development of timely and precise diagnostic strategies 28.

Clinically, numerous landmark trials have addressed critical management problems in AP, such as feeding time and modality, cholecystectomy timing in gallstone-related AP, and infected necrosis therapy. Although current therapies, such as nutritional interventions and surgical procedures, can offer some symptomatic relief, they often fail to tackle the underlying causes of pancreatitis. These conventional approaches manage the disease's effects but do not directly target the complex molecular and cellular pathways driving its progression. Recent advancements in nanomedicine, immune modulation, and precision diagnostics, however, have introduced promising new avenues for more focused and effective treatment.

Recently, in 2023, Liu *et al*. 30 outlined several potential targets within the inflammatory microenvironment of acute pancreatitis and highlighted opportunities for designing functional nanomaterials against these targets. In 2023 Cai *et al*. 31 designed biomaterial-based nanoagents, including biodegradable polymers, lipids, or metal-organic frameworks (MOFs), to target specific tissues for precise drug delivery and therapeutic effects. In 2025, Coman *et al*. 32 provided an overview of the latest clinical and experimental findings on the use of antioxidants in AP. In 2025, Zhang *et al*. 33 summarized the current research trends in nanomedicine for pancreatitis, focusing on both treatment and diagnostic aspects. In 2025, Hong *et al*. 34 conducted a meta-analysis to evaluate the effectiveness of stem cell-derived extracellular vesicles (SC-EVs) in the treatment of severe acute pancreatitis (SAP), providing valuable evidence for their potential clinical application. In 2025, Michetti *et al*. 35 discussed the complex role of autophagy in Pancreatic ductal adenocarcinoma (PDAC) and assessed the potential of modulating autophagy as a therapeutic approach. However, none of these reviews delved into integrated therapeutic strategies that address the full spectrum of pancreatitis. In this review, we aim to provide a timely and integrative update that bridges existing knowledge with emerging therapeutic strategies, offering a broader perspective on pancreatitis treatment.

Recent research on pancreatitis has advanced with targeted therapies using synthetic and biomimetic nanocarriers for controlled drug delivery, as well as gene therapies and stem cell-based approaches for reducing inflammation and promoting tissue regeneration 36,37. However, current advancements have focused on anti-inflammatory mechanisms as primary therapeutic targets for pancreatitis and its associated conditions, as anti-inflammatory treatments help in alleviating chronic inflammation, minimizing tissue damage, and inhibiting disease progression, thereby enhancing recovery and overall patient well-being. In 2021, Akshintala *et al*. 38 summarized the meta-analysis for non-steroidal anti-inflammatory drugs (NSAIDs) for pancreatitis. In 2022, Zhang *et al*. 39 discussed the understanding of the pancreas-intestinal barrier axis. In 2023, Halbrook *et al*. 40 discussed the complexity of the pancreatic tumor microenvironment (TME) and interactions among the numerous cell types within this niche. In 2024, Shi *et al.* 41 developed an engineered bio-heterojunction with robust ROS-Scavenging and Anti-Inflammation for targeted acute pancreatitis. In 2025, Jiang *et al*. 42 discussed quercetin's multi-target mechanisms and its advantages over conventional therapies. Since the anti-inflammatory therapy of pancreatitis and concomitant diseases have developed rapidly in the last several years, herein we offer a timely summary. Based on this research development, we have compiled a table (**Table 1**) that outlines all the mechanistic therapeutic approaches for treating pancreatitis.

**Scheme 2:**
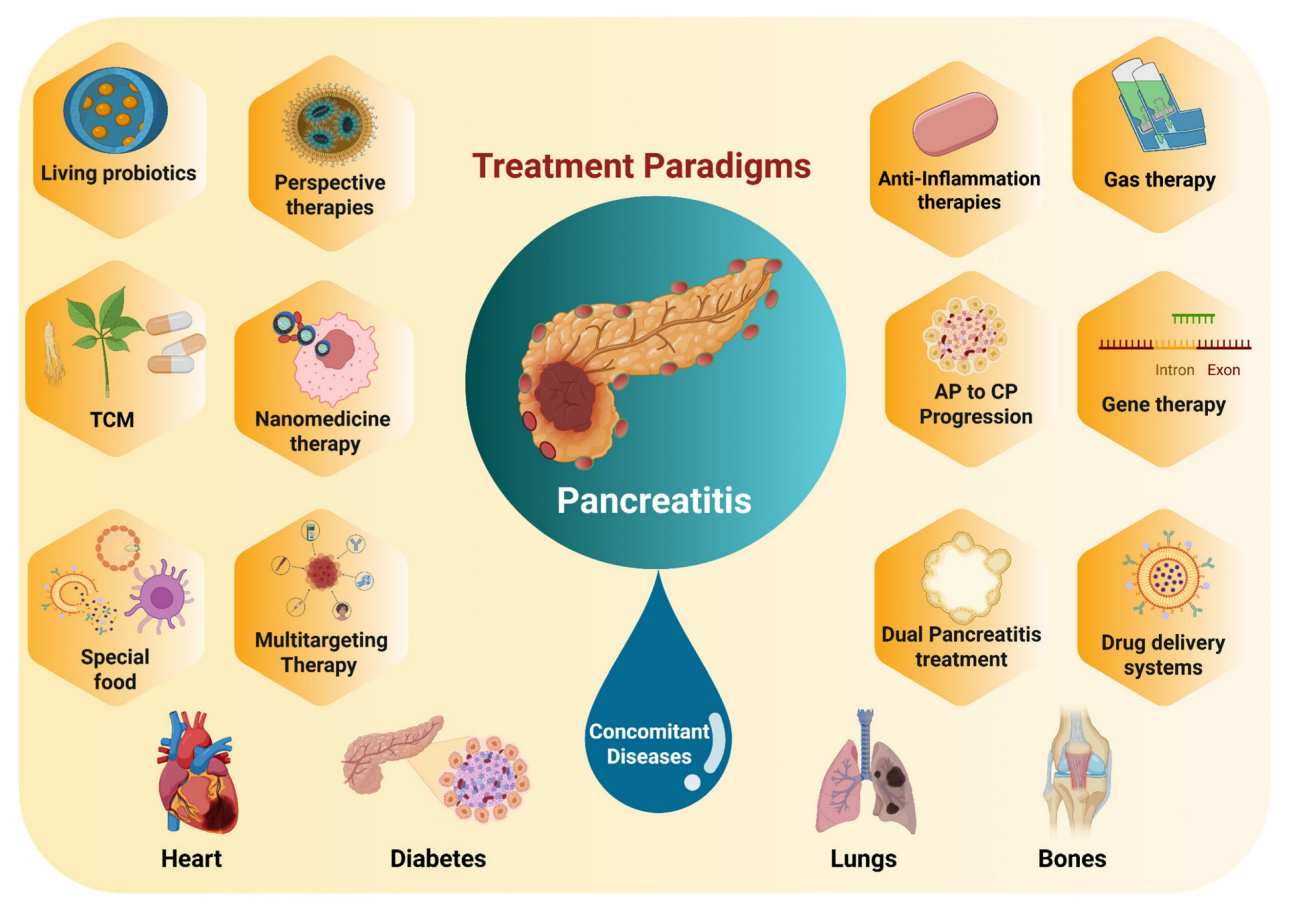
Overview of the treatment paradigms for pancreatitis and its concomitant diseases.

This review highlights the most recent advancements in anti-inflammatory therapies for pancreatitis and associated diseases (**Scheme 2**). We begin with illustration of current pathological understanding of pancreatitis and its progression from acute to chronic stage. Furthermore, we discuss how nanotechnology, particularly synthetic and biomimetic nanocarrier-based systems, has emerged as a promising approach to enhance the targeted delivery of anti-inflammatory agents, improving bioavailability and reducing systemic side effects. Importantly, we highlight the potential of gas-based therapies, traditional Chinese medicines, gene therapy, specialty food, living probiotics, exosomes, and even multitargeted approaches to enhance the therapeutic efficacy for pancreatitis and concomitant diseases. Despite these advancements, challenges such as treatment stability, immune response modulation, and scalability have been discussed. In addition, we present a detailed overview of pancreatitis which outlines the therapeutic paradigms involved in the disease, current therapeutic options, and the potential side effects these treatments may have on other organs. Generally, by integrating the most recent research and therapeutic developments, this review seeks to provide a comprehensive and updated perspective on pancreatitis management, highlighting the need for treatments that target the disease at its core while minimizing off-target effects, ultimately guiding the future of personalized care for pancreatitis patients.

## 2. Acute to chronic pancreatitis

Acute, recurrent acute, and chronic pancreatitis can arise from a variety of causes and are marked by progressive tissue injury, persistent inflammation, fibrosis, scarring, and eventual loss of pancreatic function 57. While not all individuals who develop CP have a history of initial or repeated acute episodes, acute flare-ups can still occur on a background of established CP 58. Recurrent episodes of AP may serve as an early warning sign of chronic disease progression. Supporting this, recent findings from the Hungarian Pancreatitis Research Group revealed that nearly half of the individuals who experienced three or more episodes of AP were simultaneously diagnosed with CP, highlighting the clinical importance of early recognition and intervention 59.

Alcohol remains the most common underlying factor in the progression of pancreatitis, and even moderate consumption has been shown to accelerate pancreatic injury 60. Other significant contributors include cigarette smoking and elevated triglyceride levels. Hereditary pancreatitis, a rare familial form of chronic pancreatitis, often presents in childhood or adolescence as repeated episodes of acute inflammation 61. This condition is frequently associated with autosomal dominant mutations in the PRSS1 gene, which encodes cationic trypsinogen particularly the R122H and N29I variants. However, some familial cases occur without identifiable mutations, even when there is a clear inheritance pattern involving multiple affected first- or second-degree relatives across generations. Mutations in several other genes encoding pancreatic enzymes (e.g., CTRC, CPA1, PNLIP, CEL), enzyme inhibitors (like SPINK1), and ion channels (including CFTR and CLDN2) can increase susceptibility to chronic pancreatitis 62. These genetic variations may lead to increased cellular stress, premature intracellular activation of digestive enzymes, and impaired secretion from acinar or ductal cells, all of which contribute to disease progression 63. Patients may initially present with a sentinel episode followed by recurrent acute episodes before structural pancreatic changes are detectable by imaging methods such as CT, MRI, or endoscopic ultrasound (EUS) or, in infants, by transabdominal ultrasound (TUS). When no obvious environmental trigger (like alcohol or hypertriglyceridemia) is present, the condition may be classified as idiopathic or linked to an underlying genetic mutation 64. Variants associated with lipid metabolism, such as those seen in type I, IV, or V hyperlipidemia due to mutations in LPL and related genes, can also predispose individuals to acute pancreatitis 65.

The field of pancreatic genetics has evolved rapidly since the initial identification of PRSS1 mutations in hereditary pancreatitis 66. These discoveries have reinforced the concept of pancreatitis as a disease of autodigestion, driven by early activation of digestive proteases within the pancreas. While this enzymatic activation initiates the condition, the extent and severity are primarily shaped by inflammatory cells that mediate both local damage and systemic immune responses. AP, recurrent acute pancreatitis (RAP), and CP are now viewed along a disease continuum. In many cases, chronic alcohol intake or genetic predispositions push a sentinel AP event toward RAP and eventually CP 67.

Interpreting genetic findings in AP can be challenging without long-term follow-up, as some cases may progress to RAP or CP over time 68. Many of the known pancreatitis-associated genes encode proteins that are highly expressed in the pancreas, including digestive enzymes and their inhibitors 69. Functional studies have grouped these genetic variants into distinct pathogenic pathways that contribute to the initiation and progression of pancreatitis 70.

The progression from AP to RAP and eventually CP is driven by several critical mechanisms, including the necrosis-fibrosis cascade, cellular senescence, and the activation of pancreatic stellate cells (PSCs). Persistent necrosis leads to chronic inflammation and fibrosis, setting off a cycle of continuous tissue damage and impaired function. Cellular senescence in acinar and ductal cells contributes to this process, as these senescent cells release pro-inflammatory mediators and matrix-degrading enzymes, sustaining the inflammatory environment. In parallel, the activation of PSCs, which transform into myofibroblasts, plays a central role in fibrosis by producing excessive extracellular matrix components. Together, these mechanisms drive the irreversible transition to chronic pancreatitis, extending beyond isolated episodes of inflammation.

**Figure 1:**
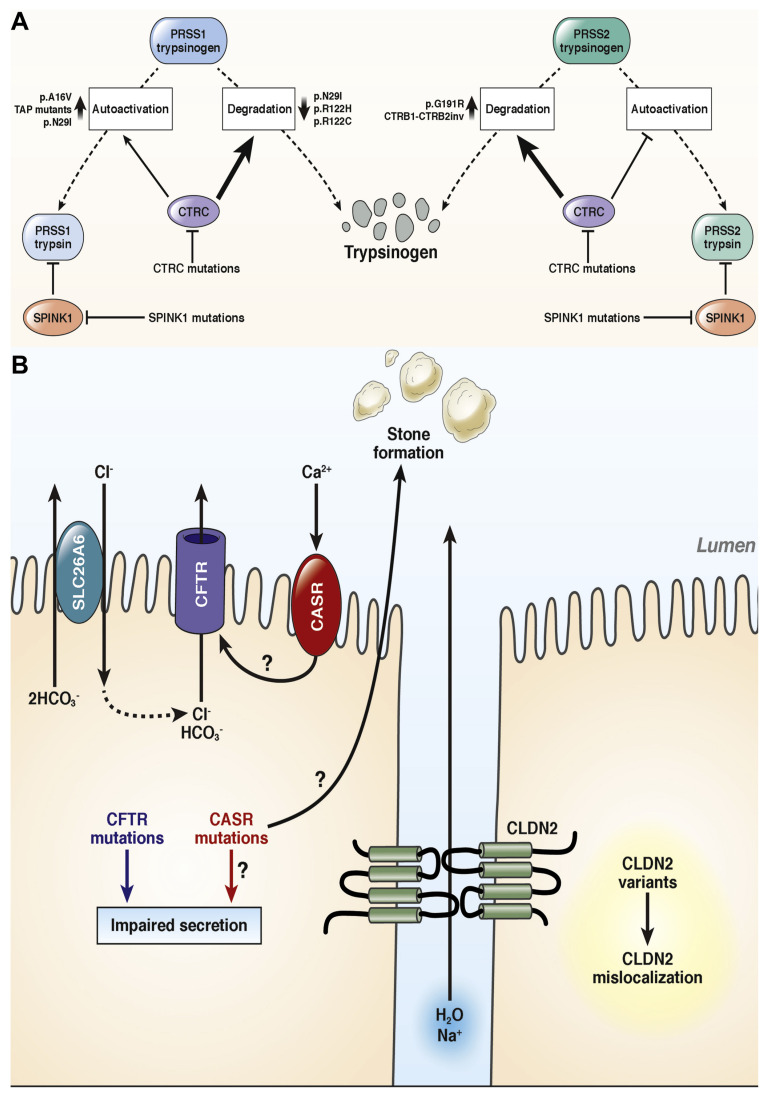
** (A)** Genetic risk factors linked to the pathogenic pathway that is dependent on trypsin. **(B)** Risk factors linked to genetics and the ductal pathologic route. Adapted with permission from ref. 71 Copyrights 2019, Elsevier.

Mayerle *et al.* 71 discussed the development of pancreatitis can follow several distinct mechanistic pathways, including the ductal route, misfolding-dependent processes, and trypsin-dependent mechanisms. One critical trypsin-dependent pathway involves autoactivation, where trypsin itself activates additional trypsinogen molecules. This premature, intra-pancreatic activation can also be initiated by the lysosomal enzyme cathepsin B. To counteract such activation, the pancreas has protective mechanisms, including inhibition of trypsin by the serine protease inhibitor Kazal type 1 (SPINK1) and degradation of trypsinogen by enzymes like chymotrypsin C (CTRC) and cathepsin L. Although CTRC's main role is to promote trypsinogen degradation, it can also enhance activation by modifying the trypsinogen activation peptide into a shorter form that is more easily activated by trypsin (**Figure 1A**). This delicate regulatory balance can be disrupted by specific trypsinogen mutations that override CTRC's regulatory effects, leading to excessive and pathological trypsin activity within the pancreas. While genetic studies in humans have strongly implicated trypsinogen autoactivation and CTRC-mediated degradation in influencing pancreatic trypsin levels, the roles of cathepsins B and L remain less clearly defined due to a lack of equally robust genetic evidence.

In addition to trypsin-related pathways, ductal dysfunction also contributes to pancreatitis risk, particularly through defects in the cystic fibrosis transmembrane conductance regulator (CFTR). CFTR is a chloride and bicarbonate channel located on the apical surface of epithelial cells (**Figure 1B**). Mutations affecting CFTR channel function or membrane expression can produce a wide range of clinical manifestations, from asymptomatic carriers to individuals with full-blown cystic fibrosis. Although the overall impact of CFTR variants on chronic pancreatitis (CP) risk is more modest than earlier thought, recent large-scale cohort studies have confirmed that CFTR mutations can still play a pathogenic role. For example, individuals heterozygous for the common p. F508del mutation have a modestly increased risk of CP (odds ratio ~2.5), while the p.R117H variant is associated with an approximate fourfold increase in risk. In cases where individuals carry one severe and one moderate CFTR mutation (compound heterozygotes), the risk of CP is significantly elevated and may be considered causative. However, the contribution of common CFTR polymorphisms, such as T5 or TG12, and bicarbonate-defective CFTR variants that do not cause cystic fibrosis remains a subject of ongoing debate, as most evidence to date does not strongly link them with CP.

AIP is a rare and distinct form of chronic pancreatitis that can present with acute episodes 72. Initially, before the term "autoimmune pancreatitis" was formally established, it was described by various names, including chronic pancreatitis with hypergammaglobulinemia, lymphoplasmacytic sclerosing pancreatitis with cholangitis, chronic sclerosing pancreatitis, pseudotumorous pancreatitis, duct-narrowing chronic pancreatitis, non-alcoholic duct-destructive chronic pancreatitis, and chronic pancreatitis with autoimmune features 73. Currently, AIP is classified into two subtypes based on histopathological features. Type 1, also known as lymphoplasmacytic sclerosing pancreatitis, is considered part of the broader IgG4-related disease spectrum. Type 2, often referred to as idiopathic duct-centric pancreatitis, is characterized by granulocytic epithelial lesions. Interestingly, similar lesions have also been identified in children with autoimmune sclerosing cholangitis who show no pancreatic involvement. Type 1 AIP is frequently associated with other manifestations of IgG4-related systemic disease, including sclerosing cholangitis, retroperitoneal fibrosis, sclerosing sialadenitis, interstitial nephritis, thyroiditis, lymphadenopathy, and interstitial pneumonia 74. This condition often involves the formation of mass-like lesions in affected organs, along with increased tissue infiltration by IgG4-positive plasma cells or elevated serum IgG4 levels. In contrast, type 2 AIP tends to be more closely associated with inflammatory bowel disease and may carry a less favorable clinical course. From an epidemiological and clinical outcome standpoint, it is well established that with each successive episode of AP, patients may progress closer to developing CP 75. Therefore, analyzing biomarkers collected during AP episodes, regardless of their direct association with the pancreas, could prove valuable in identifying early-stage chronic pancreatitis (ECP). The next section of this study focuses on exploring these diagnostic possibilities 76,77.

**Figure 2:**
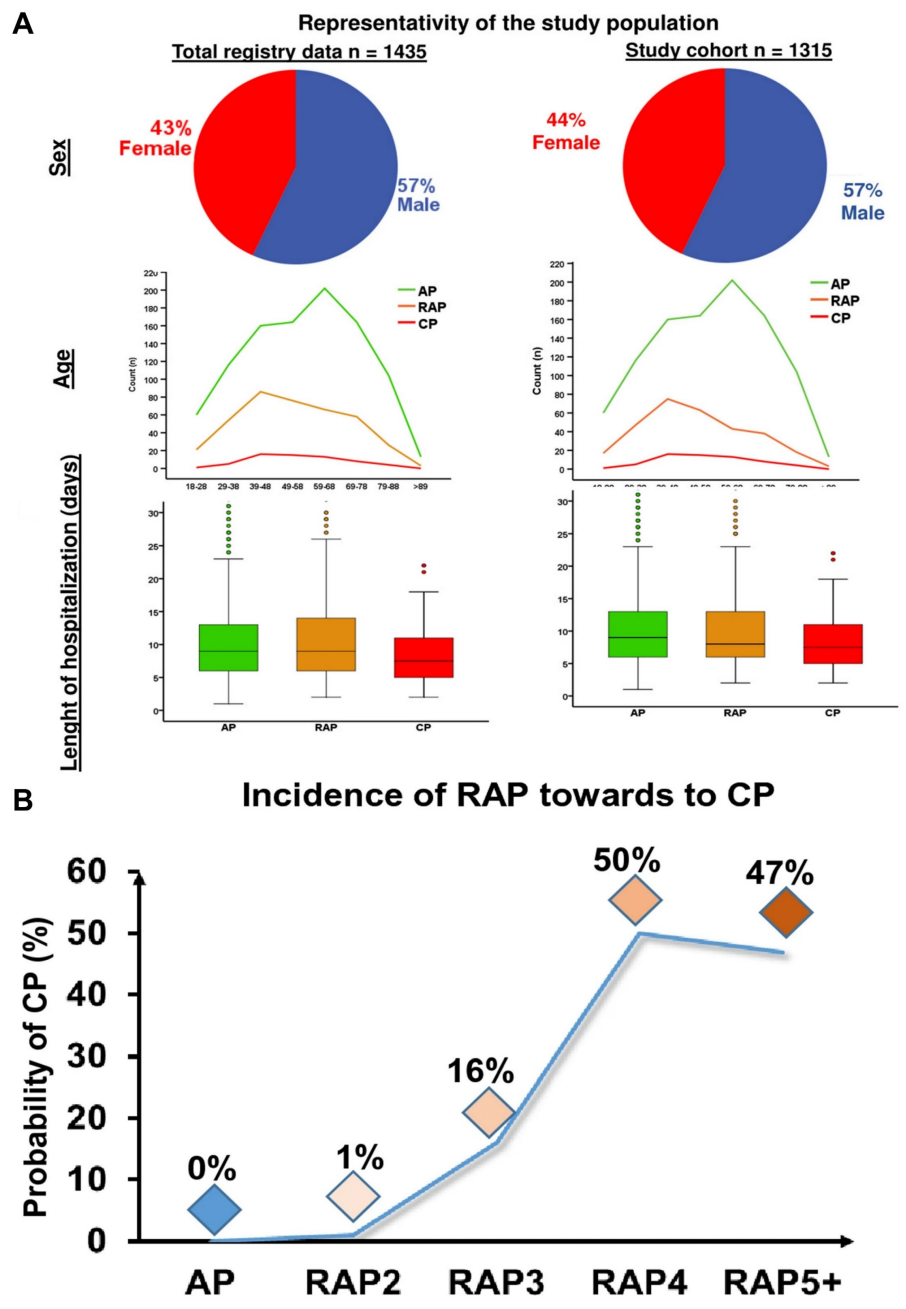
** (A)** representativeness of the population under investigation**. (**B**)** Development of CP following recurring occurrences (RAP) following the initial AP event. Adapted with permission from ref. 78 Copyrights 2021, Springer Nature.

Hegyi *et al.* 78 performed a comprehensive analysis on a total of 102 biomarkers, with the primary findings summarized in **Figure 2A**. The study also assessed whether an increased number of acute inflammation episodes correlates with a heightened risk of developing (CP). Results showed that individuals with only one or two AP episodes had less than a 1% chance of progressing to CP. However, the likelihood rose significantly with repeated episodes those who experienced three AP events had a 16% risk, while those with four or more episodes faced a 50% chance of developing CP. These data suggest that experiencing three or more acute pancreatitis episodes should be considered a strong predictive factor for the future development of chronic pancreatitis (**Figure 2B**).

## 3. Nanomedicines for pancreatitis therapy

### 3.1 Nanomedicines vs free drugs

Nanocarrier-based drug delivery systems provide an effective solution to the challenges posed by traditional free drugs, such as limited bioavailability and non-specific distribution 79. These systems, which include synthetic carriers like lipid nanoparticles, liposomes, and polymeric nanoparticles, as well as biomimetic options like biomacromolecule-based nanoparticles and extracellular vesicles, significantly enhance drug stability, solubility, and targeted delivery 80. By functionalizing nanocarriers with specific targeting agents, drugs can be directed to precise sites of action, improving therapeutic outcomes while minimizing side effects 81. Additionally, nanocarriers enable controlled and sustained drug release, reducing the need for frequent dosing and enhancing patient adherence, while also decreasing systemic toxicity by ensuring localized drug delivery. This makes nanomedicines a powerful and adaptable approach in modern drug therapy 82.

Nanomedicine represents a cutting-edge approach in drug delivery, offering several key advantages over traditional small-molecule drugs 83. These include the ability to deliver treatments directly to diseased tissues, release therapeutic agents in a controlled manner over time, minimize off-target toxicity, and improve overall drug safety. Such capabilities are especially beneficial in treating pancreatitis, where inflammation is localized and conventional treatments often cause systemic side effects 84. In recent years, various nanoparticle-based systems have been investigated for their ability to target the pancreas more effectively, regulate oxidative stress, and modulate inflammatory pathways 85. These include biomimetic nanoparticles cloaked in immune cell membranes, antioxidant-loaded carriers, and nanoscale materials that mimic the activity of protective enzymes. Preclinical studies have shown that these nanoformulations can enhance drug efficacy and reduce complications, making them promising tools for the future of pancreatitis therapy 86.

Cerium-based nanomedicines represent an emerging class of therapeutic agents with considerable potential in treating a variety of diseases, particularly those involving oxidative stress and inflammation. Cerium, a rare earth metal with unique redox properties, can exist in both its trivalent (Ce^+3^) and tetravalent (Ce^+4^) states, making it highly effective in modulating ROS and restoring cellular balance. When engineered into nanoparticles, cerium compounds can harness these properties to deliver targeted therapeutic effects with high specificity, making them an ideal candidate for the treatment of conditions such as pancreatitis, cancer, and neurodegenerative diseases. By exploiting the dual functionality of cerium nanoparticles acting as both antioxidants and calcium stabilizers novel nanomedicines can address the complex cellular disruptions associated with disease progression, offering promising avenues for innovative and effective treatments 87.

AP arises from multiple causes and is characterized by inflammation of the pancreas. During this inflammatory process, the pancreatic environment becomes rich in ROS, inflammatory cells, enzymes, protons (H^+^), and other bioactive molecules, all of which contribute to the progression of AP through various mechanisms 88. Recently, several nanotechnology-based drug delivery systems and nanomedicines have been developed to leverage this inflammatory microenvironment, improving both the diagnosis and treatment of inflammatory conditions such as AP 89. This approach utilizes specific markers within the inflamed tissue to enhance therapeutic precision. Targeting these sites can be achieved either passively or actively 90. Passive targeting exploits abnormal biochemical features of the inflamed pancreas such as altered pH levels, elevated ROS, and increased enzyme activity as well as the enhanced permeability of the tissue, allowing drugs to accumulate more effectively 91. Active targeting, on the other hand, involves designing delivery systems that specifically bind to receptors that are overexpressed in the inflamed pancreatic tissue, thereby directing treatment precisely to the affected area and improving therapeutic outcomes 92,93.

### 3.2 Synthetic nanocarriers

#### 3.2.1 Solid lipid and polymer-based nanoparticles

Lipid and polymeric nanoparticles are among the most thoroughly studied materials in nanomedicine. It is therefore unsurprising that these types of nanocarriers have also been utilized in the treatment of pancreatitis 94. For instance, Cervin *et al*. 95 encapsulated somatostatin, a peptide hormone used in pancreatitis treatment within lipid liquid crystal materials to improve its clinical effectiveness. This nano-formulation extends the peptide's half-life from just a few minutes to about an hour. Additionally, polymeric nanoparticles within a carefully controlled size range can exploit the ELVIS (Enhanced Lipid Vehicle-Induced Selectivity) effect extravasation through leaky blood vessels followed by capture by inflammatory cells. This phenomenon enables passive targeting of nanoparticles to inflamed tissues, reducing systemic side effects. Increasingly, researchers are leveraging the ELVIS effect for drug delivery in inflammatory conditions, including pancreatitis, to enhance targeted therapy. The ELVIS effect is highly dependent on the specific model of AP, and its effectiveness can differ across various experimental setups. This passive targeting approach capitalizes on the changes in vascular permeability and increased blood flow that occur during inflammation, which may vary in different AP models. In models like cerulein-induced AP, the inflammatory response often leads to greater vascular permeability, allowing for enhanced accumulation of nanoparticles in the affected tissue. However, in other models, such as bile duct ligation-induced AP, changes in the microvascular structure, along with the development of fibrosis, can hinder the efficacy of passive targeting strategies, reducing nanoparticle uptake in the pancreas. Additionally, factors like the duration of the disease, the severity of the inflammatory response, and the immune environment in each model play a critical role in the success of passive targeting approaches, further influencing their overall effectiveness 96.

Yang *et al*. 97 developed poly (lactic-co-glycolic acid) PLGA nanoparticles (CQ/pDNA/PLGA NPs) co-loaded with plasmid DNA (pDNA) and chloroquine diphosphate (CQ). In this approach, pDNA was compacted by CQ before being encapsulated within the PLGA nanoparticles. This formulation not only enhanced transfection efficiency but also improved targeting to CT26 transplanted tumors. More importantly, in a mouse model of L-arginine-induced acute pancreatitis (AP), these CQ/pDNA/PLGA nanoparticles showed remarkable targeting capabilities. Building on this work, the same team later introduced tamoxifen-loaded PLGA nanoparticles (TAM-NPs) combined with CQ-loaded liposomes (CQ-LPs). This combined treatment alleviated AP and severe acute pancreatitis (SAP) by modulating IDO signaling pathways in bone marrow-derived mesenchymal stem cells. These studies highlight how PLGA nanoparticles exploit the ELVIS effect, facilitating their uptake by neutrophils and pancreatic macrophages, which in turn enables efficient drug delivery and therapeutic benefit in AP. This macrophage-targeting property of PLGA nanoparticles has also been utilized for precision therapies in pancreatic cancer.

#### 3.2.2 Inorganic-based nanoparticles

Treatment of inflammatory diseases often involves the use of exogenous agents that mimic natural enzymes and exhibit antioxidant properties. Inorganic nanoparticles, compared to polymer or lipid-based ones, typically offer a more uniform size distribution and smaller particle sizes 98. Their surface characteristics also make them particularly suitable for attaching ligands. As a result, synthetic enzymes stand out as highly effective tools for restoring imbalanced redox homeostasis 99. According to Hu *et al*. 100 polyvinylpyrrolidone-stabilized molybdenum diselenide nanoparticles (MoSe2-PVP NPs) were easily synthesized and demonstrated efficient enzyme-mimicking properties, capable of scavenging free radicals such as 3-ethylbenzothiazoline-6-sulfonic acid (ABTS), hydroxyl radicals (•OH), and superoxide anions (•O_2_^-^). In animal models of acute pancreatitis (AP), MoSe2-PVP NPs showed significant protective effects and notably improved cell survival under oxidative stress induced by H_2_O_2_. Similarly, Prussian blue, a traditional dye historically used to treat thallium and radioactive element poisoning, has been engineered into nanoparticles (PB NPs) with remarkable physical, chemical, optical, and magnetic properties, along with high chemical stability. Recently, PB NPs have attracted attention due to their ability to mimic various antioxidant enzymes, making them promising candidates for treating inflammatory diseases.

**Figure 3:**
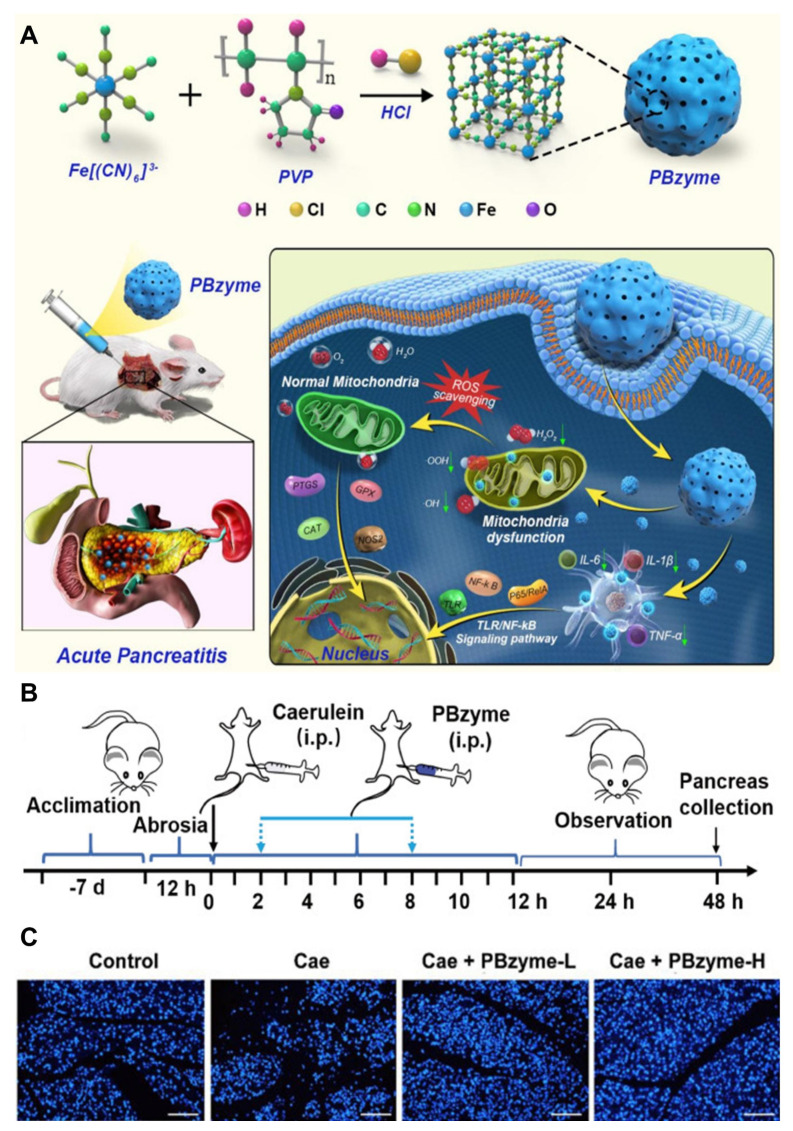
** (A)** Diagrammatic representation of the therapeutic mechanism of PBzyme, which prevents acute pancreatitis by preventing the activation of the TLRs/NF-κB signaling pathway linked to oxidative stress and inflammation. **(B)** PBzyme pretreatment for AP: overall experimental protocol by blocking the production of inflammatory factors *in vivo*. **(C)** Assessment of pancreatic apoptosis with TUNEL fluorescent staining (Scale bar: 100 μm). Adapted with permission from ref. 101 Copyrights 2021, IVYSPRING.

Zheng *et al*. 101 developed prussian blue nanoparticles (PBzyme) that act as nano-enzymes capable of neutralizing various reactive oxygen species (ROS) and pro-inflammatory molecules such as hydroxyl radicals (•OH), hydroperoxyl radicals (•OOH), and hydrogen peroxide (H_2_O_2_), as illustrated in **(Figure 3A)**. PBzyme reduces oxidative damage, including lipid peroxidation, necrosis, and nucleic acid injury, by inhibiting the Toll-like receptor (TLR)-mediated NF-κB signaling pathway. The preventive effects of PBzyme were evaluated *in vivo* using the caerulein-induced AP mouse model, which is widely used due to its ease of induction, non-invasiveness, reproducibility, and histological similarity to human AP. Treatment with PBzyme, especially at higher doses (Caerulein + PBzyme-H group), led to decreased serum amylase (AMS) and lipase (LPS) levels compared to untreated AP mice **(Figure 3B)**. Apoptosis in pancreatic tissues, assessed by TUNEL staining, was elevated in the caerulein-only group but significantly reduced in mice treated with both low and high doses of PBzyme **(Figure 3C)**. The therapeutic action of PBzyme in preventing AP appears to involve its antioxidant and anti-inflammatory properties. By scavenging ROS and suppressing the TLR/NF-κB pathway associated with inflammation and oxidative stress, PBzyme enhances the body's natural defense mechanisms, resulting in effective prevention of AP. Besides targeting TLR/NF-κB signaling, PBzyme's mechanism aligns with several established pharmacological approaches for managing AP. Compared to current AP medications, PBzyme offers advantages such as straightforward synthesis and stable *in vitro* preservation. Moreover, the production process has been optimized to allow gram-scale synthesis of uniform PBzyme nanoparticles.

Given its simple composition, scalable manufacturing, inherent bioactivity, biosafety, and significant preventive efficacy, PBzyme presents a promising candidate for AP prevention. Future studies using non-human primate models are necessary to further elucidate PBzyme's mechanisms, assess long-term safety, and confirm its preventive benefits. These findings provide a foundation for the clinical development of PBzyme for AP and potentially other ROS- and inflammation-related diseases.

Apart from the metallic inorganic nanoparticles discussed above, selenium-based non-metallic inorganic nanoparticles can also be used to alleviate pancreatitis. Abdel-Hakeem *et al*. 102 utilized antioxidant-rich selenium nanoparticles, sized between 10 and 45 nm, to help restore both endocrine and exocrine functions in the pancreas of mice with AP. Meanwhile, non-metallic nanoparticles like porous silica are known for their excellent drug-loading capacity. Although chitosan oligosaccharides (COS) possess antioxidant properties, their widespread, non-targeted distribution in the body limits their effectiveness in producing significant therapeutic outcomes *in vivo*. Zeng *et al*. 103 loaded chitosan oligosaccharides (COS) into porous SiO_2_ materials to create a complex (COS@SiO_2_) that releases COS gradually in response to acidosis caused by SAP in pancreatic tissues. In SAP mouse models, the controlled release of COS effectively reduced systemic inflammation and oxidative stress markers, inhibited the expression of NF-κB and the NLRP3 inflammasome by activating the Nrf2 pathway, and ultimately lessened pancreatic and secondary lung tissue damage. However, it has also been reported that intravenous administration of commonly used inorganic nanoparticles may promote tumor formation by facilitating the spread of breast cancer cells, which raises concerns about their safety and limits their use in medical applications 104,105.

#### 3.2.3 Mesoporous organosilica nanocarriers

Mesoporous silica nanoparticles (MSNs) have attracted significant interest due to the rapid development of mesoporous materials. These nanoparticles offer unique advantages, including a high surface area, tunable pore sizes, remarkable stability, and a diverse range of framework compositions 106. Over the years, a variety of silica-based agents for diagnostic and therapeutic applications have been developed, benefiting from their recognition as safe by the FDA. However, MSNs face challenges such as cumulative toxicity and concerns regarding their long-term safety. These issues stem from the stable Si-O-Si framework of MSNs and their limited ability to degrade in biological systems 107.

To address these concerns, efforts have focused on improving the biodegradability and biosafety of MSNs without compromising their advantageous physicochemical properties. One promising approach is the hybridization of MSNs with organic components, which enhances their biocompatibility 108. Mesoporous organosilica nanoparticles (MONs) are formed by incorporating organic groups into the silica structure at a molecular level using sol-gel processes. This integration is achieved by utilizing bissilylated organosilica precursors along with structure-directing agents (SDAs). Additionally, selective etching methods can be employed to produce hollow mesoporous organosilica nanoparticles (HMONs), which possess a considerable amount of internal void space 109. This hollow architecture and the unique organic/inorganic hybrid silsesquioxane framework of HMONs enable them to overcome limitations inherent in conventional MSNs, such as suboptimal drug loading capacity and poor degradation rates. Furthermore, HMONs can be tailored with advanced functionalities, such as multimodal imaging capabilities and stimuli-responsive degradation, by carefully selecting appropriate organosilica precursors 110. These modifications make HMONs highly versatile and promising for biomedical applications. The size of nanoparticles plays a crucial role in their biological behavior and effectiveness in theranostic applications. Nanoparticles smaller than 50 nm are capable of penetrating deeper tissues and circulating in the bloodstream for extended periods. Recent studies have focused on HMONs in the size range of 50-200 nm, though sub-50 nm HMONs have shown enhanced tumor accumulation, prolonged blood circulation, and reduced risk of vascular occlusion 111. These characteristics lead to better-targeted delivery, improved biocompatibility, and overall enhanced therapeutic efficacy 112.

**Figure 4:**
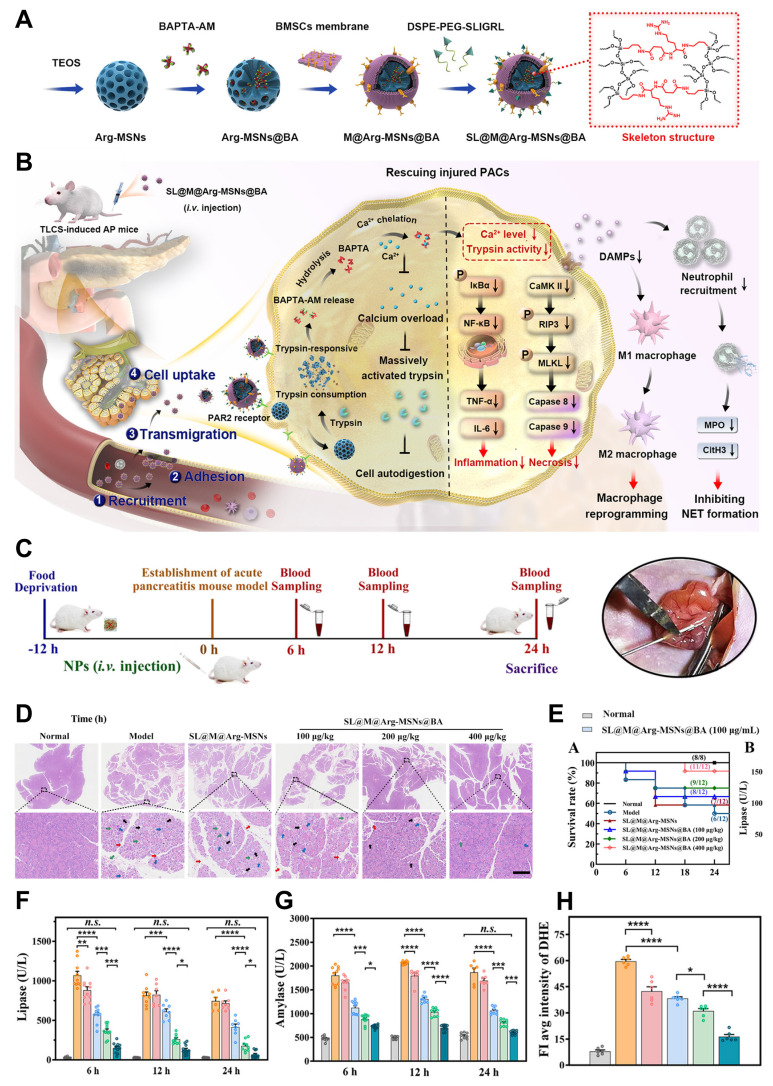
** (A)** Diagrammatic Representation and SL@Arg-MSNs@BA Preparation. **(B)** Treatment Mechanism in a Mouse Model of Acute Pancreatitis Caused by Retrograde Infusion of Sodium Taurocholate. **(C)** Experimental therapy process flowchart. **(D)** Pancreatic H&E staining. **(E)** AP mice's survival rate following various treatments. **(F)** The levels of serum lipase **(G)** and amylase. **(H)** semiquantitative findings regarding its mean fluorescence intensity. Adapted with permission from ref. 114 Copyrights 2024, American Chemical Society.

Huang *et al*. 113 have provided a comprehensive review of the synthesis methods and the latest advancements in sub-50 nm HMONs for theranostic applications. Wang *et al***.** 114 developed an organosilica precursor incorporating an arginine-based amide bond, which can be cleaved by trypsin. This precursor was incorporated into the mesoporous silica framework to form trypsin-responsive organo-bridged mesoporous silica nanoparticles (Arg-MSNs@BA). These nanoparticles were designed to encapsulate 1,2-bis (2-aminophenoxy) ethane-N,N,N,N′-tetraacetic acid (BAPTA-AM, BA), a membrane-permeable calcium chelator, to achieve controlled release of the drug and rapidly eliminate excess intracellular calcium during the early stages of AP **(Figure 4A-B)**. To enhance the specificity and targeting ability of the nanoparticles, the Arg-MSNs@BA were further functionalized with a cell membrane derived from bone marrow mesenchymal stem cells (BMSCs), creating SL@M@Arg-MSNs@BA. This functionalization was designed to promote precise targeting of injured cells, immune evasion, adhesion to inflammatory endothelial cells, and migration across the endothelial barrier. Additionally, a PACs-targeting peptide (SLIGRL, SL) was introduced to the membrane, which binds to protease-activated receptor-2 (PAR2), predominantly expressed on the apical surface of PACs. As a result, the membrane-camouflaged nanoparticles are able to specifically migrate towards and adhere to the inflamed endothelium, allowing for targeted delivery to PACs. Upon internalization by PACs, the core Arg-MSNs@BA degrade rapidly in response to excessive trypsin activation. This degradation process releases the encapsulated BAPTA-AM (BA), which chelates the excess Ca^2+^ ions inside the cells. By regulating intracellular calcium homeostasis and restoring the cell's redox balance, this mechanism helps to mitigate the cascade of apoptosis and necrosis, rebalance the inflammatory microenvironment, reduce pancreatic tissue damage, and prevent damage to distant organs. Moreover, the trypsin-responsive feature of the Arg-MSNs also inhibits acinar cell autodigestion, further preventing damage to pancreatic cells.

The synergistic combination of trypsin inhibition and calcium chelation, facilitated by the biomimetic membrane, provides a comprehensive and multidimensional therapeutic approach for AP. This innovative strategy holds significant promise for enhancing AP treatment by targeting both the symptoms and the underlying causes of the disease. A treatment schedule and a snapshot of the modeling process are illustrated in (**Figure 4C**). One hour after intravenous administration, both DiR-loaded M@Arg-MSNs and SL@M@Arg-MSNs preferentially accumulated in pancreatic tissues, as evidenced by a strong fluorescence signal in the pancreas. Histopathological analysis revealed that while severe injury was observed in the pancreatic tissues of AP mice, administration of SL@M@Arg-MSNs@BA at doses of 100 and 200 μg·kg⁻¹ led to reduced lobular space widening, decreased acinar necrosis, less inflammatory cell infiltration, and moderate effects on pancreatic edema (**Figure 4D**). Treatment with SL@M@Arg-MSNs@BA significantly lowered the TCLS-induced mortality rate in a dose-dependent manner, as shown in (**Figure 4E**). Specifically, at a BA dosage of 400 μg·kg^-1^, the survival rate of AP mice dramatically improved from 50.0% to 91.6% after a single dose. Interestingly, increasing the BA dosage beyond this point did not result in further improvement of pancreatic damage or survival rates, which aligns with previous studies suggesting an optimal therapeutic dosage range.

Regarding typical biomarkers for AP, such as lipase and amylase, significant elevation was observed shortly after the TCLS insult, with increases of 36.0 and 3.8 times, respectively, at the 6-hour mark. These elevated levels remained high at 24 hours (**Figure 4F-G**). After 6 hours of treatment with blank SL@M@Arg-MSNs, lipase levels were reduced by 17.5%, while amylase levels decreased by 13.2% at the 12-hour time point. This therapeutic effect is likely due to the degradation of the Arg-MSNs backbone, which results in the competitive inhibition of excessive trypsin activation. Additionally, the SL peptide, which acts as a receptor agonist, binds to PAR2 receptors, contributing to a reduction in pancreatitis severity. Notably, the group treated with a single dose of SL@M@Arg-MSNs@BA at a BA dosage of 100 μg·kg^-1^ showed significant suppression of oxidative stress, evidenced by a 35.8% reduction in superoxide levels, as indicated by DHE fluorescence intensity, when compared to the AP model group (**Figures 4H**).

By controlling ion homeostasis and inhibiting trypsin activity, our biomimetic trypsin-responsive nanocarrier rapidly and precisely reverses PAC damage at the earliest stages of cell injury, halting the pathophysiological progression of AP and offering significant therapeutic translation potential.

#### 3.2.4 Liposome, micelles and dendrimers

**Figure 5:**
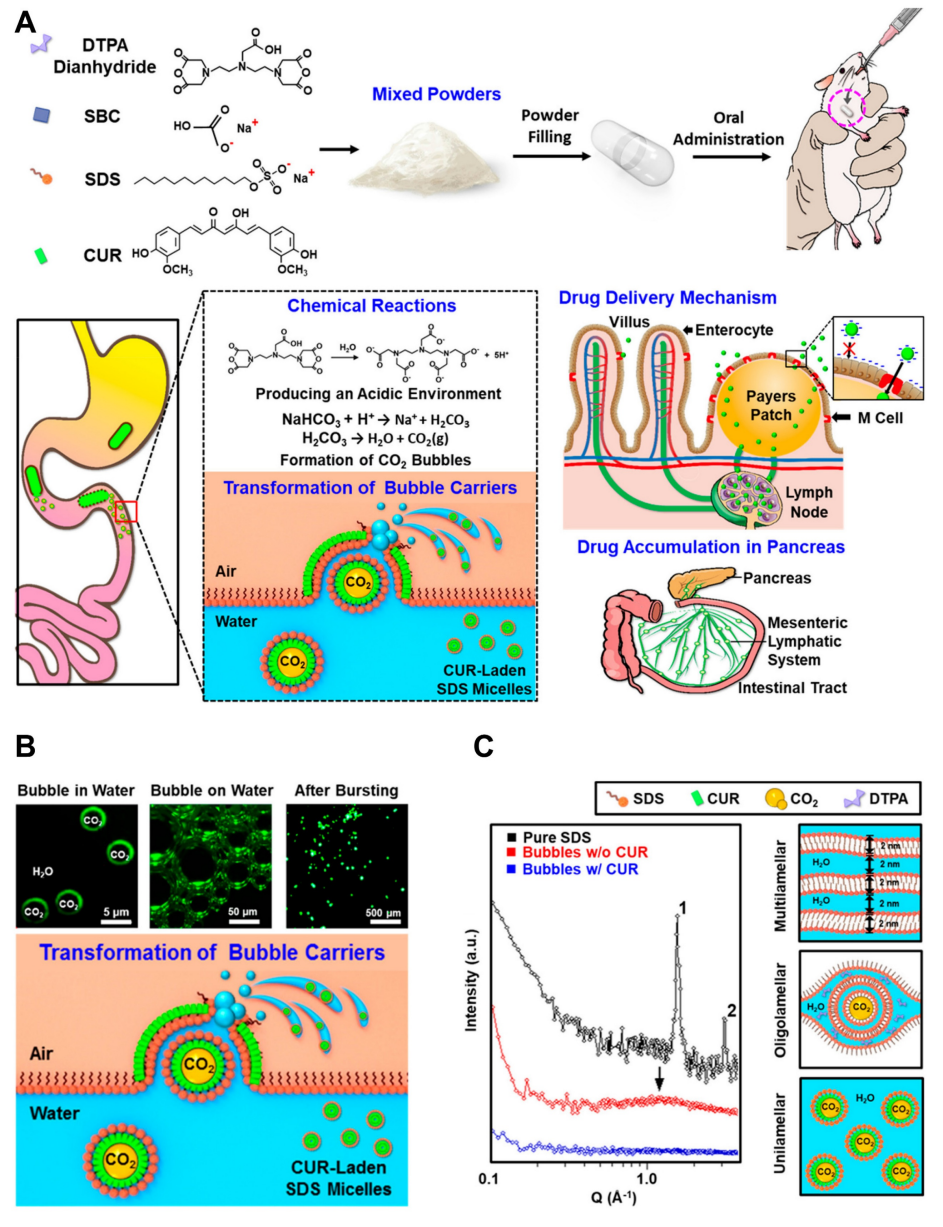
** (A)** Mechanism of CUR-loaded SDS micelle nanoemulsion creation comes from the suggested TLNS that self-assembles in the gastrointestinal tract. **(B)** Fluorescence microscopy of CUR-loaded SDS micelle nanoemulsions. **(C)** CUR-loaded SDS micelle nanoemulsions for X-ray solution scattering (SAXS). Adapted with permission from ref. 117 Copyrights 2018, American Chemical Society.

Dendrimers, liposomes, and micelles have also been described as innovative drug delivery systems for the management of pancreatitis 115. The research team led by Hsing-wen Sung created a nanocarrier system (TLNS) that resembles a transformer. The ability of TLNS to undergo structural alterations in the intestinal environment and form nanoscale micelles with curcumin (CUR) has been verified *in vitro* 116. **Figure 5A** 117 depicts the AP-targeted treatment approach using curcumin (CUR)-loaded micelles. In rats treated with TLNS, the pancreas showed nearly a 12-fold increase in CUR signal compared to those given free CUR, suggesting enhanced recovery from acute pancreatitis. These results indicate that TLNS significantly boosts the gut's capacity to absorb drugs, potentially making oral administration a more effective strategy for treating pancreatitis.

The bubble carriers' shells were composed of monolayers of fluorescent curcumin (CUR), as illustrated in (**Figure 5B**). A significant portion of these fluorescent bubbles eventually reached the water/air interface. Under microscopic observation, the nano-assemblies exhibited a double-layer fluorescence pattern. The proposed TLNS system, which delivers the drug in the form of nano-emulsions, was developed to significantly enhance the oral bioavailability of poorly water-soluble drugs. Structural changes in this TLNS were analyzed using fluorescence microscopy and small-angle X-ray scattering (SAXS). As depicted schematically in the right panel of (**Figure 5C**), the SAXS pattern of powdered sodium dodecyl sulfate (SDS) displayed two distinct peaks with a 1:2 position ratio upon hydration, indicating the surfactant molecules self-assemble into a multilamellar structure. This structure alternates between lipophilic layers (formed by two sublayers of alkyl chains) and hydrophilic layers (composed of head groups and water molecules). Literature suggests that intestinal M cells can be passively targeted by the CUR-loaded SDS micelle nano-emulsions derived from TLNS. These nano-emulsions can then be transported via the mesenteric lymphatic system to accumulate in pancreatic tissue, effectively reducing acute pancreatitis (AP) severity. This TLNS approach offers a promising strategy to efficiently emulsify a broad range of poorly water-soluble drugs within the gastrointestinal tract, improving their solubility and significantly enhancing oral bioavailability.

Additionally, dendrimers bearing polyhydroxyl groups have shown antioxidant properties, suggesting potential use in pancreatitis treatment. Two generation 5 (G5) polyamidoamine (PAMAM) dendrimers, G4.5-COOH and G5-OH, containing carboxyl and hydroxyl groups respectively, were synthesized as previously described. Their protective effects were evaluated in a caerulein-induced AP mouse model. Both dendrimers notably reduced inflammatory responses in AP mice and significantly inhibited LPS-induced inflammatory macrophage production in mouse peritoneal cells. They also caused a marked reduction in monocytes and white blood cells. Between the two, G4.5-COOH demonstrated a stronger protective effect *in vivo* against AP than G5-OH. Finally, the researchers proposed that these dendrimers reduce inflammation by suppressing NF-κB nuclear translocation in macrophages.

Each synthetic nanocarrier Solid Lipid Nanoparticles (SLNs), Poly (lactic-co-glycolic acid) (PLGA), inorganic nanoparticles, and mesoporous silica offers unique advantages and challenges for drug delivery applications. SLNs and PLGA are well-regarded for their excellent drug loading capacities and biocompatibility. PLGA stands out for its biodegradability and ability to provide controlled release, though both rely primarily on passive targeting mechanisms, which limits their precision. On the other hand, inorganic nanoparticles stand out for their high targeting efficiency, enabled by surface modifications that allow for targeted delivery, particularly in cancer therapies. However, issues regarding their biosafety and biodegradability persist, which may hinder their widespread use. Mesoporous silica also offers significant drug loading capabilities due to its high surface area and customizable pore structure, and it can be modified for targeted delivery 118. Yet, its degradation rate and long-term safety are areas that require further refinement. While SLNs and PLGA have seen more clinical success due to their well-established safety profiles, inorganic nanoparticles and mesoporous silica hold great promise for more advanced, targeted therapies. The selection of a nanocarrier ultimately depends on the specific therapeutic needs, with considerations for factors such as targeting precision, drug release control, and overall safety.

### 3.3 Biomimetic nanocarriers

#### 3.3.1 Biomacromolecule-based nanoparticles

**Figure 6:**
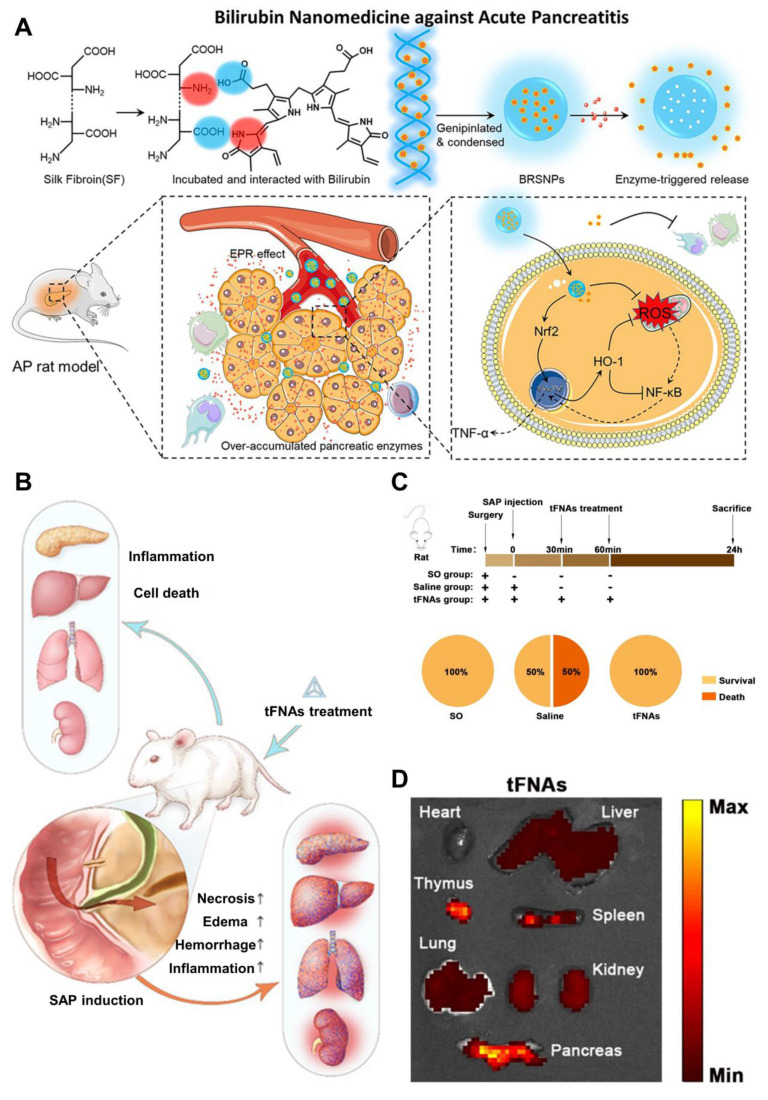
** (A)** Schematic schematic depicting synthesis, targeting, and mechanism of bilirubin nanomedicine (BRSNPs). Reproduced with permission from ref. 121 Copyrights 2020, Elsevier. **(B)** Illustration of tFNAs treatment on severe acute pancreatitis. **(C)** Experimental scheme of tFNAs treatment alleviated Severe acute pancreatitis. **(D)** Images showing the biodistribution of tFNAs in the main organs of SAP mice using fluorescence. Adapted with permission from ref. 122 Copyrights 2025, American Chemical Society.

Biological macromolecules like proteins and nucleic acids provide distinct advantages in biocompatibility and safety compared to synthetic polymers and artificial lipids 119. Additionally, certain proteins have shown responsiveness to the elevated levels of enzymes such as amylase, protease, and lipases found in pancreatitis-affected tissues 120. This property allows them to be used as smart drug carriers that enable effective passive targeting of pancreatitis lesions. Yao *et al.*^121^ utilized silk fibroin as a carrier to construct a primary three-dimensional (3D) structure resembling the DNA double helix that incorporated bilirubin, which then collapsed into nanoparticles. *In vivo* imaging studies showed that these bilirubin-loaded silk fibroin nanoparticles (BRSNPs) accumulated more efficiently in pancreatic tissue, enabling passive targeting. In both acinar cell models and L-arginine-induced AP rat models, BRSNPs released bilirubin in response to elevated pancreatic enzymes, such as trypsin, at the AP site (**Figure 6A**)**.** Bilirubin effectively reduced mitochondrial ROS production, activated the Nrf2 signaling pathway, increased HO-1 expression, and inhibited the pro-inflammatory NF-κB pathway. Treatment with BRSNPs significantly lowered various blood biomarkers, including amylase, alanine aminotransferase, aspartate aminotransferase, creatinine, and blood urea nitrogen. Additionally, it alleviated oxidative stress and lipid peroxidation in pancreatic cells, prevented edema and fibrosis, and showed superior therapeutic effects against AP compared to somatostatin or free bilirubin treatments. Beyond proteins, nucleic acids also serve as promising carrier materials. For instance, tetrahedral framework nucleic acids (tFNAs) have emerged as a novel type of nanoparticle, demonstrating strong anti-inflammatory and anti-apoptotic properties in multiple disease models.

Wang *et al*. 122 explored the therapeutic potential of tetrahedral framework nucleic acids (tFNAs) in treating SAP and its related multiorgan damage using a mouse model. Their findings showed that tFNA treatment effectively reduced SAP severity and organ injury by specifically lowering inflammatory cytokine levels in both tissues and blood, while also regulating the expression of key apoptotic and anti-apoptotic proteins (**Figure 6B**). Moreover, tFNAs helped preserve the normal structure of various organs including the pancreas, lungs, liver, and kidneys by limiting lymphocytic infiltration, a hallmark of inflammation. In the study, twenty-one mice were divided randomly into three groups: SAP treated with saline (Saline group), SAP treated with tFNAs (tFNA group), and a Sham Operation group (SO group). Following SAP induction, mice in the tFNA group received two intravenous injections of tFNAs (125 nM, 100 μL) via the tail vein at 30- and 60-minutes post-induction. The SO group underwent a sham surgical procedure, while the Saline group was administered an equal volume of saline instead of tFNAs. All animals were euthanized 24 hours after the procedure (**Figure 6C**). Using *in vivo* bioluminescence imaging, the researchers tracked the distribution of tFNAs within the body and found prominent accumulation in the pancreas, thymus, spleen, liver, and kidneys (**Figure 6D**).

In summary, this study demonstrates for the first time that tFNA therapy effectively alleviates SAP and its related multiorgan injury in mice. By modulating the release of inflammatory cytokines and regulating proteins involved in pathological cell death and apoptosis, tFNA treatment significantly curbed both local and systemic inflammation and prevented cell death, thereby halting disease progression and preserving normal tissue architecture in affected organs. Beyond protecting the pancreas from acute failure, tFNA therapy also mitigated damage to multiple organs, addressing a key complication associated with SAP.

#### 3.3.2 Extracellular vesicles

Currently, there is no particularly effective treatment for SAP, aside from supportive care, and the precise mechanisms behind AP remain incompletely understood. Recent research has highlighted the involvement of key proteins in pyroptosis, including pro-caspase-1, ASC (apoptosis-associated speck-like protein containing a CARD), and NLRP3, which are activated during AP 123. The inflammasome activation in damaged pancreatic acinar cells (PACs) leads to the release of pro-inflammatory cytokines and chemokines, exacerbating pancreatic damage through pyroptosis, a type of programmed cell death that results in acute inflammation. Therefore, targeting inflammation and preventing PAC pyroptosis are essential for effective AP treatment 124.

Multipotent mesenchymal stem cells (MSCs) show considerable promise in treating refractory pancreatic diseases by promoting angiogenesis, preventing PAC necroptosis, and supporting pancreatic tissue regeneration. Among various MSC types, hair follicle (HF)-derived MSCs are particularly attractive due to their ease of access, high proliferative capacity, and broad differentiation potential 125. However, safety concerns such as iatrogenic tumor formation and immune rejection present challenges for MSC-based therapies. To address these concerns, researchers have suggested that MSCs might act via a paracrine mechanism, in which MSCs release therapeutic factors without needing to directly migrate to the injured tissue. One key component of this mechanism is the release of small extracellular vesicles (sEVs), which range from 30 to 150 nm in size and contain a variety of proteins, lipids, cytokines, and noncoding RNAs. MSC-derived sEVs have been shown to alleviate inflammatory conditions such as ulcerative colitis, acute liver injury, and renal injury 126.

**Figure 7:**
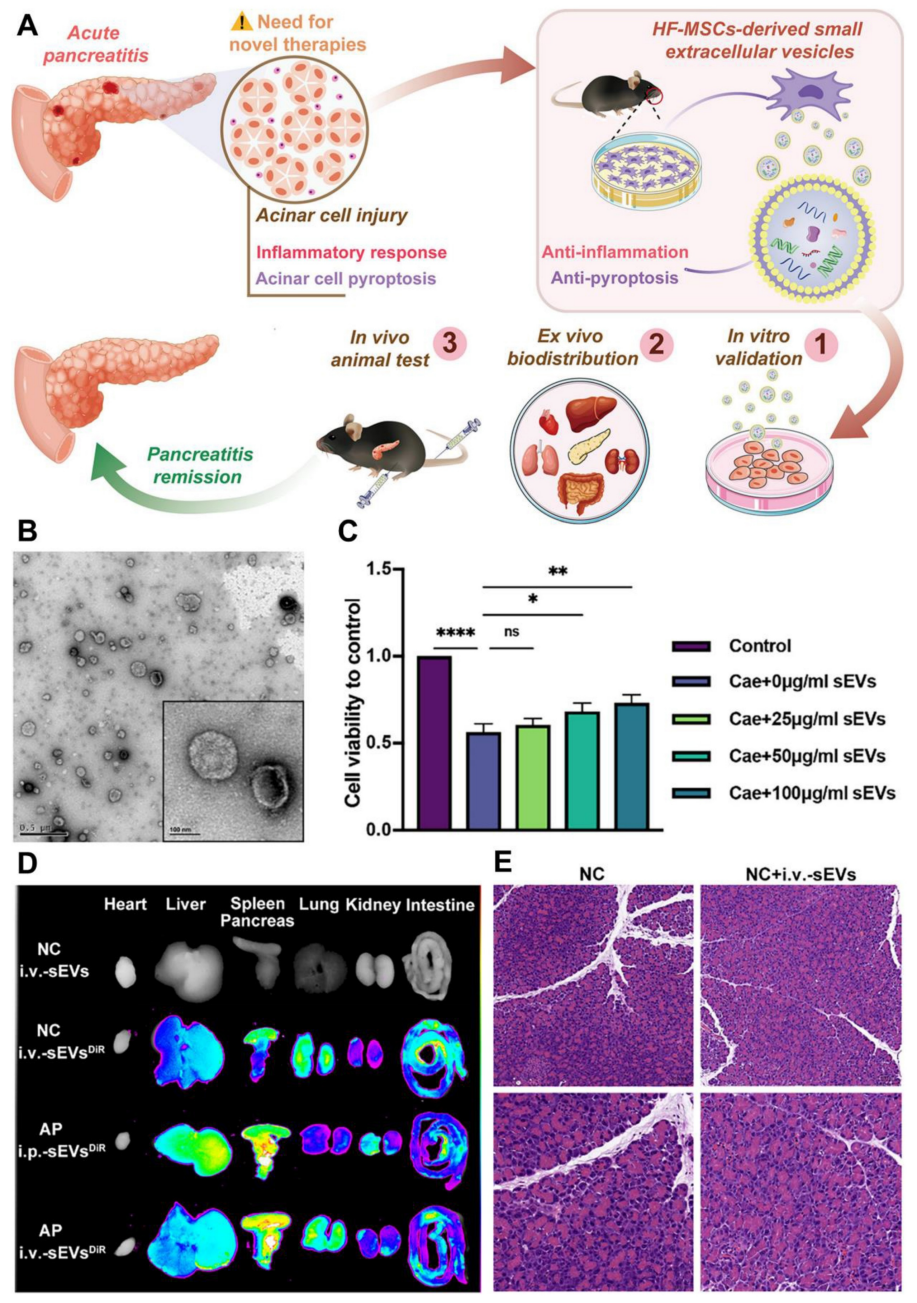
** (A)** Demonstration of HF-MSC-sEVs attenuated AP and the potential mechanism. **(B)** Examining HF-MSC-sEV morphology under TEM. **(C)** Effects of different HF-MSC-sEV concentrations on MPC-83 cell viability. **(D)** NIRF measurement of the fluorescence intensities of the main organs in mice and *ex vivo* organ imaging. **(E)** Typical pathological alterations in pancreatic tissues stained with H&E sections at ×200 and ×400 magnification. Adapted with permission from ref. 127 Copyrights 2022, Elsevier.

In a study by Li *et al*. 127 the therapeutic potential of HF-MSC-derived sEVs for treating AP was investigated, focusing on their mode of action (**Figure 7A**). The study tested the effects of intraperitoneal (i.p.) and intravenous (i.v.) administration of sEVs in mice. Transmission electron microscopy (TEM) analysis of purified sEVs revealed a distinct saucer-like structure with a lipid bilayer, confirming their typical morphology (**Figure 7B**). The results showed that higher concentrations of sEVs improved cell viability, with 100 μg/ml sEVs significantly enhancing cell survival compared to lower doses (**Figure 7C**). Biodistribution studies indicated that both i.p. and i.v. routes resulted in sEVs accumulating in the pancreas of AP-affected mice. Notably, i.p.-sEVs were also found in the liver and spleen, while i.v.-sEVs dispersed more widely across the lung, spleen, liver, and other organs (**Figure 7D**). These findings suggest that sEVs are capable of reaching the damaged pancreas, regardless of the administration route.

Histological analysis of pancreatic tissue revealed significant edema, inflammatory infiltration, and acinar cell necrosis in the AP group. In contrast, these pathological changes were less severe in the sEV-treated mice, suggesting a protective effect. The NC (normal control) group, whether or not treated with sEVs, showed no major changes in tissue pathology (**Figure 7E**). These results provide strong evidence that HF-MSC-derived sEVs could represent a promising therapeutic approach for reducing pancreatic damage in AP.

Overall, a new therapeutic effect of HF-MSC-sEVs, makes them a viable treatment option for AP. In both MPC-83 cells and AP animals, the treatment of sEVs reduced inflammation in acinar cells; the therapeutic effect may be due to the inhibition of pyroptosis signaling. Furthermore, in the caerulein-induced AP paradigm, intravenous sEV delivery was found to be more efficacious than intraperitoneal injection. In conclusion, the clinical use of extracellular vesicle-based therapeutics in incurable pancreatic illnesses may be encouraged by this cell-free method based on sEVs produced from HF-MSCs, which has significant promise as a safe and effective therapy option for AP.

#### 3.3.3. Multitargeting strategies for pancreatitis therapy

In AP, the inflammatory microenvironment is characterized by the presence of ROS, inflammatory cells, hydrogen ions (H^+^), and excess digestive enzymes, all of which contribute to the worsening of the disease through various mechanisms 128. Due to this complexity, relying on a single biomarker for diagnosis and treatment is often insufficient. The involvement of multiple inflammation-related factors opens up new possibilities for improving both the diagnosis and treatment of AP 129. Recently, there has been progress in developing multitargeted drug delivery systems. Among these, dual-targeted delivery approaches have shown greater potential by effectively exploiting the inflammatory microenvironment to enhance drug distribution and therapeutic outcomes 130,131.

Dai *et al*. 132 created a nanosystem to enable synergistic oxidation-chemotherapy with self-enhanced drug release. This system is not only sensitive to pH changes, which improves drug uptake by altering its surface charge, but it also responds to reactive oxygen species (ROS) by releasing cephaeline and β-lapachone. This dual responsiveness helps to overcome multidrug resistance within tumors and promotes the destruction of cancer cells. In another study, Gou *et al*. 133 developed surface-functionalized (SF) nanoparticles (CS-CUR-NP) by coating with chondroitin sulfate (CS). These nanoparticles are designed to release curcumin (CUR) in response to pH changes and ROS. Moreover, CS-CUR-NP can specifically deliver drugs to inflammatory sites and target macrophages, making them effective for treating ulcerative colitis. Interestingly, similar nanoparticle systems have also been engineered for the diagnosis and treatment of AP.

**Figure 8:**
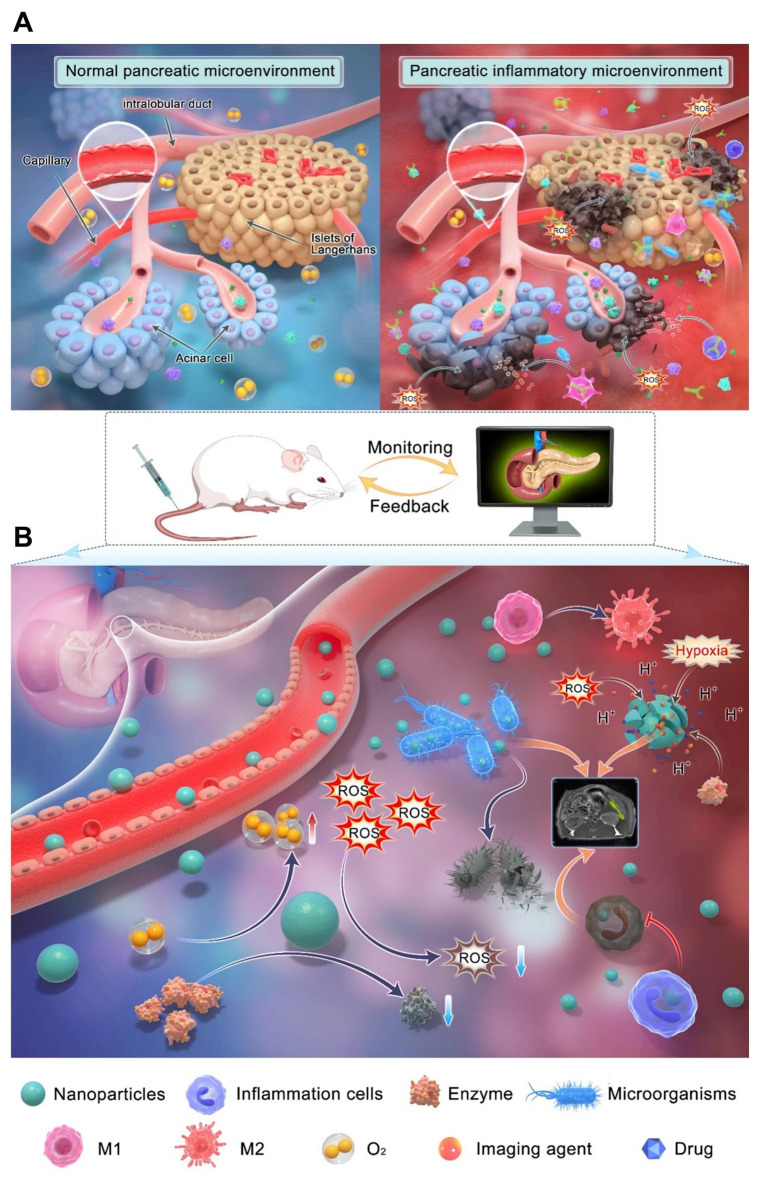
** (A)** Schematic diagram of microenvironmental targets of pancreatic inflammation. **(B)** Schematic diagram of different modes of interaction between nanoparticles and targets in the inflammatory microenvironment. Adapted with permission from ref. 30 Copyrights 2024, Springer Nature.

Liu *et al*. 30 developed nano-theranostic agent, TMSN@PM, that induces excessive ROS generation and mild acidity. This multifunctional platform offers both therapeutic treatment and MRI imaging capabilities for AP. The development of TMSN@PM represents a significant step toward combining diagnosis and therapy in AP management, and it is expected that future research will further advance multifunctional nanoplatform applications for this condition. AP has a complex pathogenesis involving factors such as trypsinogen activation, calcium overload, and mitochondrial dysfunction, alongside diverse causes and clinical symptoms, making its conventional diagnosis and treatment challenging. Early in AP, trypsin activation within pancreatic acinar cells triggers the activation of other digestive enzymes, leading to pancreatic tissue injury. The activation of immune cells like macrophages releases proinflammatory cytokines, which intensify pancreatic inflammation. Excessive ROS production further exacerbates cell damage by generating various harmful molecules. This process activates multiple signaling pathways, including nuclear factor-kappa B (NF-κB) and toll-like receptors (TLR), driving an inflammatory cascade. Additionally, pancreatic cell dysfunction and enhanced glycolysis contribute to a reduction in pH levels. As illustrated in (**Figure 8A**), the AP pathological process involves an accumulation and overexpression of substances such as H^+^, digestive enzymes, ROS, and inflammatory cells, creating an inflammatory microenvironment crucial to disease progression.

**Figure 8B** highlights how the complex microenvironmental changes in AP complicate diagnosis and treatment but also offer new therapeutic targets. Targeting multiple pathways simultaneously is likely to be more effective than focusing on a single factor. Therefore, designing multitarget nanocarriers and discovering additional targets could improve diagnostic and therapeutic approaches for AP. Advances in nanotechnology have addressed many limitations of traditional diagnosis and treatment methods. Nanomaterials with high specificity can enhance detection sensitivity and reduce response times, improving diagnostic accuracy. Various biosensors and nanoprobes have been engineered to detect inflammatory microenvironment markers. Through surface modification, nanomaterials can optimize their properties, enabling targeted accumulation in specific organs and amplifying detection signals. This has significantly improved pancreatic enzyme detection and pancreatic tissue imaging, increasing AP diagnosis rates.

On the therapeutic front, a range of nanodrugs and nanocarrier systems have been developed to repair AP-induced damage. These innovations overcome issues related to poor solubility and bioavailability of conventional drugs and enhance drug delivery efficiency. Moreover, therapeutic nanomaterials exhibiting anti-inflammatory, antioxidant, and antiapoptotic effects have been designed to target key elements within the inflammatory microenvironment. By leveraging surface functionalization and stimulus-responsive materials, drug carriers can be engineered to target abnormal biochemical markers such as elevated H^+^, ROS, and digestive enzymes within the inflammatory microenvironment of AP. This targeted delivery prolongs drug retention at inflammation sites in the pancreas and related organs, offering a promising strategy to improve treatment outcomes for AP.

Despite significant progress, nanotechnology still faces many challenges and limitations in diagnosing and treating AP by targeting the inflammatory microenvironment. One major hurdle is the unique anatomical structure of the pancreas, which includes a specialized blood-pancreas barrier (BPB) composed of the basement membrane, capillary endothelial cells surrounding glandular follicles, and other components. While this barrier protects the pancreas from pathogens, it also restricts drug penetration, resulting in few drugs being able to effectively cross it and achieve therapeutic concentrations within the organ. Currently, only a limited number of nanocarriers are designed to target the inflammatory microenvironment, leading to low delivery efficiency. Future developments should focus on designing carriers that combine the advantages of multiple delivery systems to improve both targeting efficiency and biosafety. Another limitation is the lack of well-defined, precise targets for AP due to its still unclear pathophysiology. This has limited the development of nanodrugs specifically aimed at the pancreatic inflammatory environment. Most current nanodrugs rely on passive targeting mechanisms, such as the ELVIS effect, which increases drug accumulation in inflamed tissues but also causes off-target effects and low delivery precision. Therefore, there is a need to develop actively targeted nanodrugs that recognize specific molecular markers of inflammation to enhance delivery efficiency and therapeutic outcomes 134.

Recent advances in genomics, proteomics, and metabolomics have made it possible to identify more accurate biomarkers for pancreatitis, improving early diagnosis and treatment efficacy. While designing functional nanomaterials to target these diverse markers in the inflammatory microenvironment is a promising direction, several issues hinder their widespread clinical application.

Among these, biosafety remains a critical concern. The lack of standardized protocols and regulatory guidelines for evaluating the toxicity of nanoparticle-based drug delivery systems complicates the assessment of their potential risks. Furthermore, because the human immune system's response to drugs is complex, animal models often fail to accurately replicate the absorption, distribution, metabolism, excretion, and organ-specific effects of nanomaterials in humans. Consequently, most current studies rely heavily on animal experiments, which may not fully predict human responses. Additionally, various animal models exist for AP, each reflecting different causes and features of the disease 135,136. Researchers must carefully select the most appropriate model based on its pathophysiological relevance and limitations for their specific study aims. However, the complex and multifactorial nature of human AP makes it difficult for animal models to perfectly mimic the condition, and the technology for establishing these models is still developing. To facilitate the clinical translation of nanomedicines for AP, it is essential to establish standardized scientific evaluation systems, improve animal models, develop robust evaluation criteria and testing methods, and advance research in nanotoxicology. Addressing these challenges will be crucial for safely and effectively harnessing nanotechnology in the diagnosis and treatment of AP.

## 4. Therapeutic antiinflammation strategies for pancreatitis treatment

**Figure 9:**
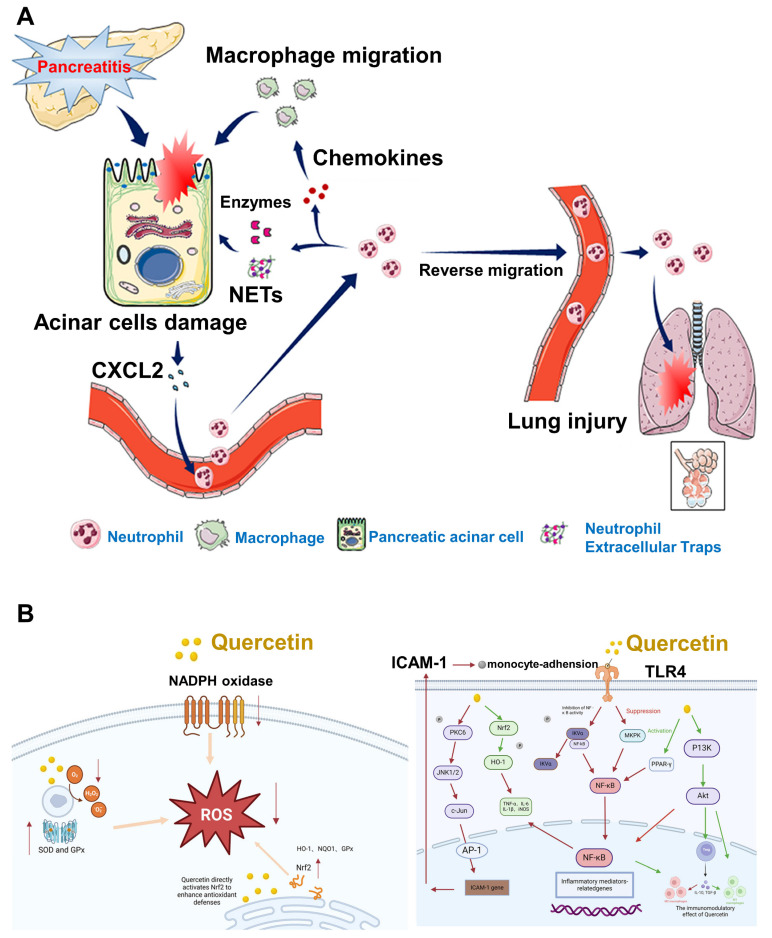
** (A)** The process by which neutrophils worsen AP. Reproduced with permission from ref. 142 Copyrights 2021, Frontiers **(B)** Mechanism of quercetin-induced Nrf2 activation and increased antioxidant defenses, and the Quercetin-mediated signaling pathway in the control of AP. Adapted with permission from ref. 145 Copyrights 2025, Frontiers.

Oxidative stress plays a pivotal role in the development of AP, stemming from a disruption in the balance between ROS and the body's antioxidant defense mechanisms 137. This imbalance causes direct cellular damage and contributes to the progression of local pancreatic inflammation into systemic complications, such as multiple organ dysfunction syndrome (MODS) 138. Despite the availability of supportive treatments like fluid replacement, nutritional intervention, and pain control, no pharmacological solution has been identified that can fully prevent or reverse the disease process. Neutrophils are central to the inflammatory cascade in AP 139. Experimental studies have indicated that reducing neutrophil numbers can lead to a notable decrease in tissue injury. However, this approach often leads to compromised immune function 140. In AP, excessive or misdirected neutrophil activity not only worsens pancreatic injury but also causes damage to remote organs, especially the lungs. Non-steroidal anti-inflammatory drugs (NSAIDs), when administered rectally prior to ERCP procedures, have been shown to reduce post-ERCP pancreatitis by limiting neutrophil migration into pancreatic tissue 141. Wan *et al*. 142 demonstrated that AP exacerbation involves the mobilization of granulocytes, accumulation of neutrophils, and the formation of neutrophil extracellular traps (NETs), as illustrated in (**Figure 9A**). This discovery introduces opportunities for more focused therapies that can attenuate the inflammatory response, minimize damage caused by neutrophils, and reduce overall immune system compromise.

Quercetin, a naturally occurring flavonoid found in various fruits and vegetables, has gained attention due to its potent antioxidant capabilities. It helps protect cells by neutralizing ROS, reducing lipid peroxidation, and stimulating the synthesis of intracellular glutathione (GSH), a key antioxidant molecule. Studies in chronic kidney disease models have shown that quercetin can reverse endothelial dysfunction by lowering oxidative damage. Further *in vitro* evidence from Hossein *et al*. 143 revealed that quercetin treatment significantly reduced ROS levels, elevated sulfhydryl group concentrations, and enhanced cell viability in endothelial cells subjected to oxidative stress. In pancreatitis models, quercetin has consistently demonstrated its ability to diminish oxidative damage in the pancreas by both scavenging harmful radicals and activating antioxidant enzymes, including superoxide dismutase (SOD), catalase (CAT), and glutathione peroxidase (GSH-Px). In cerulein-induced AP models, Faiza *et al***.** 144 observed that quercetin treatment markedly suppressed ROS production while increasing the levels of these protective enzymes. These findings suggest that quercetin not only protects pancreatic tissues from oxidative stress but also enhances the body's internal antioxidant defense systems. The Nrf2 pathway, a crucial regulator of oxidative stress responses, is also positively influenced by quercetin. Jiang *et al*. 145 demonstrated that quercetin activates Nrf2 signaling, which boosts the production of GSH and enhances cellular resilience to oxidative injury mechanisms depicted in (**Figure 9B**).

In addition to its antioxidant functions, quercetin also demonstrates significant anti-inflammatory activity. It modulates key pro-inflammatory pathways, notably NF-κB and MAPK, which are both heavily involved in the progression of pancreatic inflammation. Quercetin's capacity to inhibit these signaling cascades helps to reduce cytokine release and tissue injury. The mechanism suggests that quercetin has the potential to serve as a broad-acting therapeutic agent in AP by simultaneously addressing oxidative stress and inflammation.

Nevertheless, a major barrier to the clinical use of quercetin is its limited bioavailability. Poor absorption and rapid metabolism restrict its therapeutic effectiveness, particularly in acute and severe conditions like AP. Advanced drug delivery systems such as nanoparticles and liposomal encapsulation have been proposed to enhance its systemic availability, but further research is needed to ensure their safety and efficacy in clinical settings. There is also insufficient data regarding optimal dosing regimens, potential side effects, and interactions with standard medications such as corticosteroids or N-acetylcysteine (NAC). This raise concerns that combining quercetin with other therapies might lead to unpredictable outcomes. Even so, quercetin's broad pharmacological profile targeting oxidative damage, immune dysfunction, and inflammation positions it as a promising candidate for future AP therapies. Compared to single-target agents, quercetin offers the advantage of a multi-target approach. Although current evidence from laboratory models is encouraging, clinical trials are necessary to determine its practical utility. With additional research, quercetin could transition from an experimental compound to a mainstream therapeutic option for pancreatitis and related inflammatory diseases.

AP involves extensive pancreatic injury characterized by vacuolization, necrotic cell death, and early activation of digestive enzymes. To avoid such cellular damage, it is crucial to maintain a tightly regulated balance among Ca^+2^ release from intracellular reservoirs, Ca^+2^ influx, and extrusion processes 146. Various triggers of AP disrupt this equilibrium, leading to intracellular Ca^+2^ overload in acinar cells, which contributes to mitochondrial dysfunction through the formation of the mitochondrial permeability transition pore (MPTP), subsequent ATP depletion, and cell necrosis. Oxidative stress has been implicated in the progression of AP and may influence Ca^+2^ dynamics within the acinar cells 147. However, its precise mechanistic role remains unclear, and clinical trials using antioxidant therapies have largely been unsuccessful. It is hypothesized that the pattern of cell death in AP may be partially dictated by ROS production 148.

Recent preclinical findings suggest potential therapeutic strategies to prevent Ca^+2^-induced mitochondrial injury in AP. These include: (1) limiting Ca^+2^ release via inhibition of inositol 1,4,5-trisphosphate receptors (IP3Rs), (2) reducing Ca^+2^ influx by targeting Orai1 channels, and (3) blocking MPTP formation. Several candidate drugs targeting these pathways are nearing clinical evaluation, offering hope for treating a disease that currently lacks effective pharmacological interventions 149. ROS are generated both within pancreatic acinar cells and by infiltrating immune cells, such as neutrophils, during the inflammatory cascade, thereby contributing to local and systemic injury. Mitochondrial complexes I and III are primary sources of ROS during normal respiration. While ROS have been shown to serve essential signaling roles in physiological contexts, excessive ROS production can damage DNA, proteins, and lipid membranes, exacerbating cellular injury. Thus, oxidative stress in AP reflects a complex interplay between ROS generation and the body's antioxidant defenses. The extent of pancreatic injury likely correlates with the severity and persistence of this imbalance 150.

**Figure 10:**
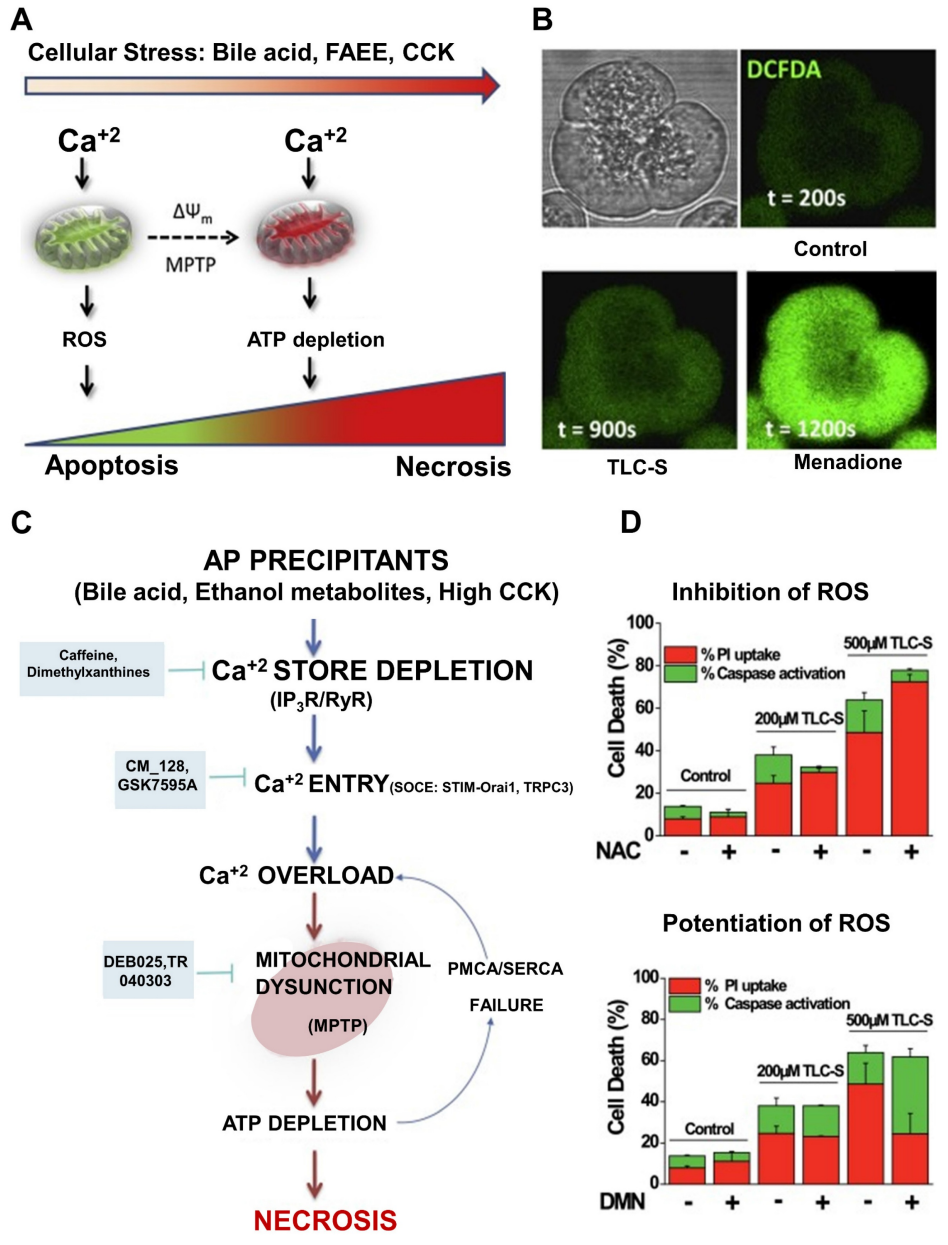
** (A)** A hypothetical model depicting the consequence of higher stress levels on pancreatic acinar cell fate. **(B)** Typical visuals and graphs illustrating Ca^2+^-dependent increases of ROS caused by the bile acid taurolithocholic acid sulphate (TLCS). **(C)** Schematic depicting translational techniques for treating AP. **(D)** Patterns of cell death in pancreatic acinar cells from humans and mice. Adapted with permission from ref. 151 Copyrights 2016, Elsevier.

Criddle *et al*. 151 has shown that the bile acid taurolithocholic acid sulphate (TLCS) induces ROS production in both human and murine pancreatic acinar cells, as visualized using confocal microscopy and the ROS-sensitive fluorescent probe CM-DCFDA (**Figure 10A**). This ROS generation was localized to the mitochondria, confirmed through co-localization with MitoSOX and Mitotracker dyes. The ROS response was significantly reduced by the antioxidant N-acetylcysteine. Additionally, TLCS was found to trigger sustained elevations in cytosolic calcium levels ([Ca^+2^]c), with the observed mitochondrial ROS formation being dependent on this Ca^+2^ rise, as measured by Rhod-2 fluorescence (**Figure 10B**). It has been suggested that ATP depletion is a critical factor leading to necrotic cell death, and that cellular demise may transition from apoptosis to necrosis based on the intensity and duration of the insult. Disruption of Ca^+2^ homeostasis essential for proper exocrine pancreatic function results in calcium overload in acinar cells. This, in turn, induces mitochondrial permeability transition pore (MPTP) formation, impairs ATP production, and leads to necrosis. While low levels of oxidative stress may favor apoptosis, potentially offering protection in AP by limiting inflammation compared to necrosis, the use of antioxidants may disrupt these natural defense mechanisms. This could explain why antioxidant therapies have largely failed in clinical settings for AP.

Recent studies have proposed that protecting mitochondria from calcium-dependent injury may offer a promising strategy for AP treatment. Preclinical models support several approaches: (1) blocking inositol 1,4,5-trisphosphate receptors (IP3Rs) to limit Ca^+2^ release, (2) inhibiting Orai1 channels to prevent Ca^+2^ entry, and (3) preventing MPTP formation. Selective compounds such as CM-128, which target calcium-induced mitochondrial dysfunction in acinar cells, are advancing toward clinical trials and may offer significant benefits for a disease that currently lacks specific pharmacological interventions (**Figure 10C**). The inhibition of ROS-induced apoptotic cell death is arguably the most significant aspect of antioxidants' documented detrimental effects in clinical studies. The balance of pancreatic acinar cell death was switched from apoptosis to necrosis by inhibiting the production of ROS caused by bile acid (**Figure 10D**). A key reason for the disappointing performance of antioxidants in clinical trials may lie in their inhibition of ROS-mediated apoptotic pathways. When bile acid-induced ROS production is suppressed, the mode of cell death in acinar cells may shift from apoptosis to necrosis thereby worsening inflammation and contributing to disease severity.

Anti-inflammatory strategies are crucial for the effective treatment of pancreatitis because inflammation is the primary driver of its progression and complications. While approaches targeting oxidative stress, immune dysregulation, or tissue damage are valuable, they often address secondary aspects of the disease rather than directly managing the underlying inflammatory process. Inflammation accelerates tissue injury and fibrosis, contributing to chronic complications such as pancreatic dysfunction and organ failure. By focusing on anti-inflammatory mechanisms, therapies can target the root cause of these pathological changes, modulating inflammatory mediators like cytokines, immune cells, and inflammatory signaling pathways. This approach not only helps to alleviate immediate symptoms but also plays a key role in preventing long-term damage and reducing the risk of further disease progression. Moreover, anti-inflammatory therapies can be integrated with other treatment modalities, such as antioxidant therapies or those aimed at tissue repair, to provide a more comprehensive treatment. This combination offers a promising avenue for improving outcomes in patients with pancreatitis, reducing the need for invasive interventions, and potentially enhancing overall quality of life. Therefore, prioritizing anti-inflammatory strategies may offer a more focused and effective treatment approach compared to other therapeutic mechanisms.

### 4.1 Traditional Chinese Medicine therapy

Hepatic, biliary, and pancreatic tumors continue to pose significant challenges in modern oncology. These malignancies are frequently diagnosed at advanced stages, which limits the effectiveness of available treatments and contributes to generally poor prognoses. Critical gaps in the management of these cancers include the need for early detection tools, the advancement of personalized therapeutic approaches, strategies to counteract drug resistance, and the enhancement of palliative care protocols. Overcoming these challenges holds great potential for improving treatment outcomes and elevating the standard of care 152. TCM, grounded in a personalized and holistic healing philosophy, offers an increasingly recognized avenue for cancer management. With centuries of empirical application, TCM brings a vast array of plant-based compounds known for diverse therapeutic effects 153. Many of these compounds are used in synergistic herbal formulations and have shown notable anti-cancer activity. They also serve as promising scaffolds for novel drug development 154. Clinical evidence suggests that patients with cancer who receive TCM interventions often experience improved tumor responses, enhanced quality of life, and prolonged survival emphasizing the clinical relevance of TCM-based oncology therapies 155.

In China, TCM has become an integral component of cancer care due to its affordability, accessibility, and long-standing cultural acceptance. Beyond direct anti-tumor effects, TCM has been shown to alleviate the adverse effects of conventional therapies and improve patient prognosis 156. Combined regimens involving TCM and standard therapies such as chemotherapy have been associated with extended survival and better symptom control in patients with hepatocellular carcinoma, lung cancer, and even in cases involving depression-related comorbidities 157. Kaempferol (KA), a flavonoid found in various medicinal herbs, has garnered attention for its antioxidant properties, especially in treating inflammatory digestive diseases characterized by oxidative imbalance. SAP is known to be exacerbated by mitochondrial dysfunction and the excessive generation of ROS, emphasizing the importance of antioxidants that can support mitochondrial health. Despite its potential, KA's clinical use is hampered by its low bioavailability and the need for high therapeutic doses, which may cause side effects 158.

#### 4.1.1 TK therapy

**Figure 11:**
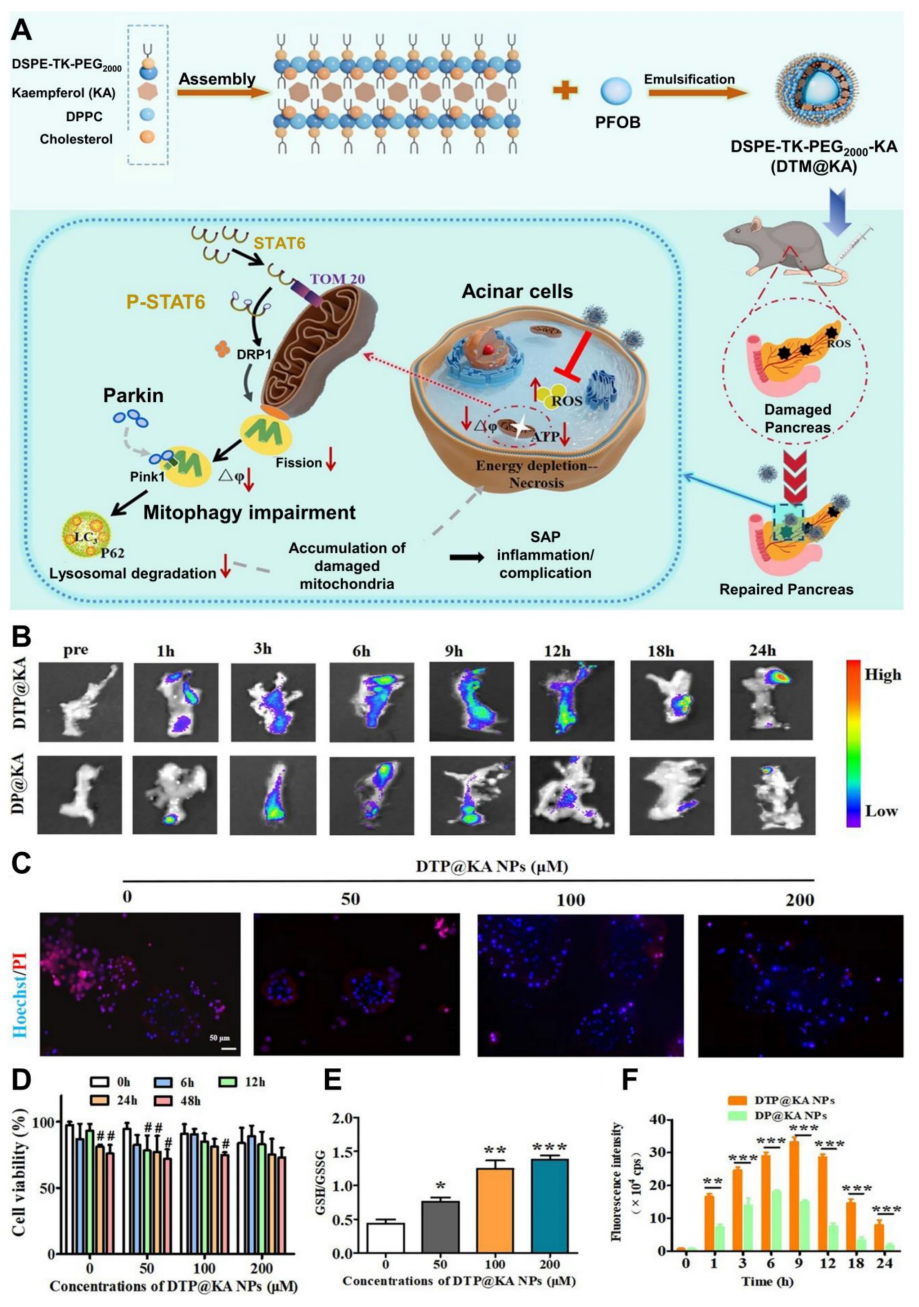
** (A)** Diagram showing the synthesis and protective role of DTM@KA NPs in experimental SAP, along with a potential mechanism relating to mitochondrial function and oxidative stress controlled by TOM20-STAT6-Drp1-mitophagy signaling. **(B)** Images of the pancreas from SAP model mice treated with DTP@KA NPs for 24 hours *in vitro* using DIR fluorescence**. (C)** Images of the pancreas from SAP model mice treated with DTP@KA NPs for 24 hours *in vitro* using DIR fluorescence. **(D)** Viabilities of main PAC co-incubated for 0, 6, 24, and 48 hours with varying concentrations of DTP@KA NPs**. (E)** The ratio of glutathione (GSH) to oxidized glutathione (GSSG) in primary PAC treated with varying doses of DTP@KA NPs. **(F)** Quantitative evaluation of the SAP model mice's pancreas. Adapted with permission from ref. 159 Copyrights 2024, Springer Nature.

To address these limitations, Wen *et al***.** 159 designed a novel liposomal delivery platform using a thioketal (TK)-modified DSPE-PEG2000 structure, named DSPE-TK-PEG2000-KA (DTM@KA NPs). This nanocarrier system significantly enhanced KA's bioavailability and minimized adverse effects. The formulation worked by stabilizing the redox balance within cells reducing GSH depletion and activating the Nrf2 signaling pathway. It also synergized with KA to suppress improper mitochondrial fusion, promote appropriate fission, and restore mitophagy, all of which are critical for reducing inflammation and cell death. Ultimately, this intervention preserved mitochondrial integrity and restored ATP production, which is vital for pancreatic acinar cell (PAC) recovery and function. The presence of the TK bond in the formulation was shown to be crucial for its enhanced therapeutic efficacy compared to a non-TK version. Thus, the resulting nanomedicine (DSPE-TK-MPEG2000-KA, DTM@KA NPs) was developed to regulate mitochondrial function through antioxidant and anti-inflammatory mechanisms, including improved mitochondrial fission and activation of mitophagy (**Figure 11A**). Imaging studies revealed that DIR-labeled DTP@KA NPs accumulated more extensively and remained longer in pancreatic tissues compared to 200 μM DP@KA NPs, demonstrating the TK group's role in targeting pancreatic injury (**Figure 11B**). These results confirmed that DTP@KA NPs are capable of effectively lowering ROS levels in primary PACs, thus limiting oxidative damage and achieving targeted delivery to injured pancreatic tissues. As shown in **Figure 11C**, DTP@KA NPs suppressed propidium iodide (PI) uptake in PACs in a dose-dependent manner. In untreated PACs, 100 μM H_2_O_2_ led to significant cellular damage at both 24 and 48 hours. In contrast, cells treated with 50 μM DTP@KA NPs showed no significant injury, even under oxidative stress, and retained better viability during prolonged H_2_O_2_ exposure (**Figure 11D**). Furthermore, as illustrated in **Figure 11E**, DTP@KA NPs treatment resulted in increased GSH/GSSG ratios across concentrations of 0, 50, 100, and 200 μM indicating restored redox homeostasis. Finally, the targeted accumulation of 200 μM DTP@KA NPs in the pancreas was validated to assess their therapeutic value in experimental SAP. As presented in **Figure 11F**, DIR-labeled DTP@KA NPs showed prolonged retention in pancreatic tissues compared to their non-TK-modified counterparts, reinforcing their suitability as a targeted therapy for pancreatic inflammation and injury.

#### 4.1.2 DTM therapy

Within this nanodelivery system, DTM NPs are designed to actively respond to mitochondrial dysfunction by addressing oxidative stress and cellular imbalance. The thioketal (TK) bond serves as a ROS-sensitive trigger that directs the release of the therapeutic cargo specifically to inflamed, ROS-rich regions of the pancreas. Kaempferol (KA), a naturally occurring compound known for its antioxidant and anti-inflammatory effects, works in synergy with the TK linkage to restore both mitochondrial and redox homeostasis. This dual action helps to suppress tissue injury associated with SAP, reducing damage not only in the pancreas but potentially in other affected organs as well. Furthermore, DTM@KA nanoparticles contribute to the regulation of ROS levels, control excessive mitochondrial fragmentation, and support mitophagy. This is achieved in part through the activation of the STAT6 signaling pathway, facilitated by TOM20, a mitochondrial translocase protein. By enhancing the clearance of dysfunctional mitochondria, this mechanism may help contain inflammation and prevent mitochondrial-driven cell death.

#### 4.1.3 ED therapy

Emodin (ED), a potent anti-inflammatory agent, shows potential for treating acute pancreatitis (AP). As an anthraquinone derivative, ED possesses a range of beneficial properties, including anti-inflammatory, antibacterial, anti-tumor, and immunosuppressive effects. Previous research has highlighted ED's strong therapeutic efficacy in alleviating pancreatic damage, reducing related complications, and promoting tissue regeneration in the pancreas. However, its clinical use has been limited due to challenges such as poor water solubility, low bioavailability, and inadequate targeting specificity. In cases of AP, significant vasodilation and increased capillary permeability occur at the site of inflammation. Nanoparticles (NPs) can take advantage of these conditions to accumulate at the inflamed pancreatic tissue via the bloodstream. Unfortunately, once NPs enter circulation, they are often rapidly recognized and cleared by the mononuclear phagocyte system (MPS), limiting their effectiveness for targeted drug delivery to the inflamed regions. To overcome this, biomimetic modifications of NPs, such as coating them with cell membranes, have emerged as an effective strategy to evade MPS detection. Among these strategies, coating nanoparticles with macrophage membranes offers not only immune evasion but also enhanced targeting of inflammatory tissues. This is facilitated by interactions with adhesive molecules and integrins, ensuring the precise and efficient delivery of drugs to the inflamed pancreatic areas.

Metal-organic frameworks (MOFs) are materials formed by the self-assembly of metal ions or clusters and organic ligands, creating structures with internal pores. MOFs possess several advantages, including a high surface area, excellent stability, and ease of surface modification. These characteristics make MOFs highly promising for drug delivery applications. Building on these properties, Yang *et al*. 160 developed macrophage membrane-coated UiO-66-NH_2_ NPs loaded with ED (MVs-UiO-ED) for the targeted treatment of AP. This approach aims to enhance the clinical effectiveness of ED and improve its therapeutic impact on AP.

### 4.2 Gas therapy

#### 4.2.1 H_2_ therapy

Hydrogen therapy, which utilizes molecular hydrogen (H_2_), is emerging as a promising and safe treatment for a variety of medical conditions. Once considered biochemically inert, molecular hydrogen has recently been recognized for its biological reactivity, particularly its unique bio-reductive properties. It also appears to play a role in restoring the energy balance of damaged cells, acting as a homeostatic regulator. In 1975, Dole *et al*. 161 demonstrated that the tumor growth in squamous carcinoma-bearing mice could be effectively inhibited by inhaling hydrogen gas at high pressures (8 atm). The accelerating progress of hydrogen biology and its clinical applications can largely be attributed to Ohta *et al*. 162 discovery of hydrogen's antioxidant mechanisms, particularly in the context of ischemia reperfusion injury and inflammation. Over the past two decades, hydrogen gas has shown therapeutic potential in a wide variety of diseases associated with oxidative stress and inflammation, including cancer, atherosclerosis, ischemia reperfusion damage, stroke, diabetes, neurodegenerative disorders, arthritis, dermatitis, colitis, hepatitis, and pancreatitis. Due to its broad therapeutic efficacy and excellent safety profile, hydrogen therapy is transitioning from experimental settings to clinical applications.

In recent years, a variety of chemical substances, such as exosomes, polymers, and nanoparticles, have been explored to enhance the capabilities of hydrogen medicine. Among these, exosome-like nanovesicles derived from plants have garnered significant interest due to their favorable properties, such as small size, high biocompatibility, stability in acidic and alkaline conditions, cost-effectiveness, and potential for large-scale production. These nanovesicles, which are bilayer liposomal structures, have shown promise in treating conditions such as obesity, diabetic nephropathy, cancer, and liver damage caused by alcohol. Specifically, ginger-derived exosomes (GE) have been shown to activate the AhR/IL-22 pathway and modulate the gut microbiota, thereby improving conditions like colitis induced by dextran sulfate sodium. This is achieved through the regulation of tight junctions and induction of antimicrobial immunity.

**Figure 12:**
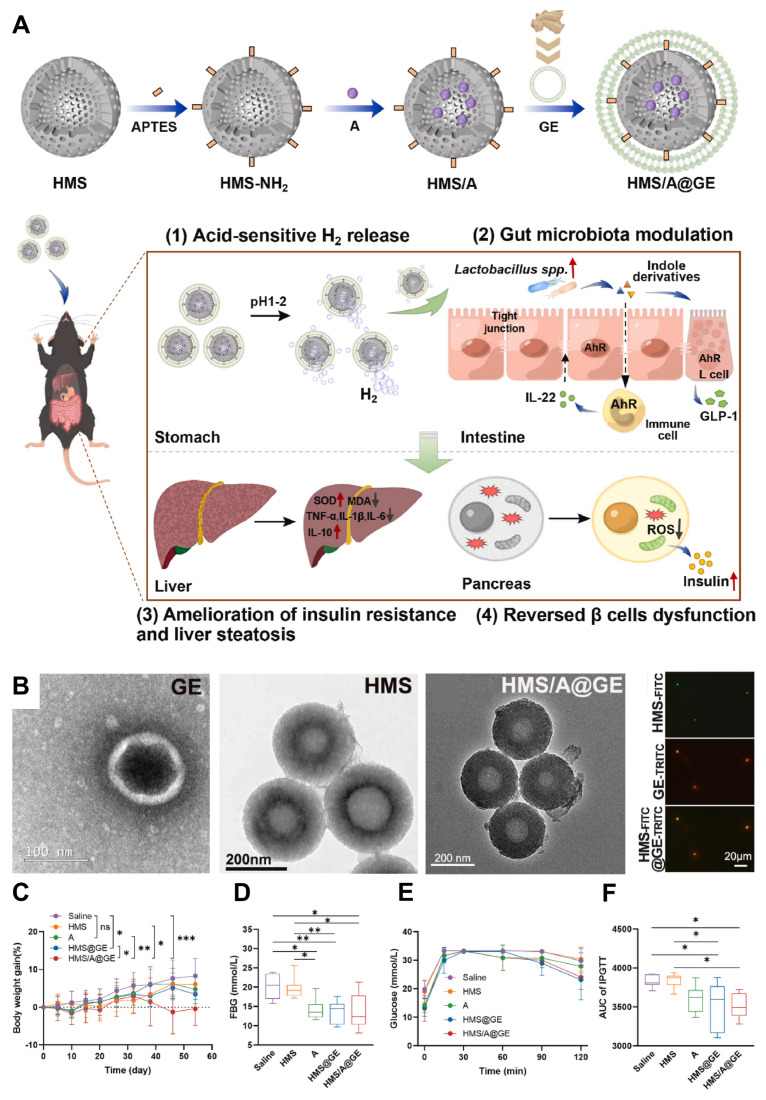
** (A)** Illustration of the fabrication and therapeutic mechanism of a biomimetic acid-responsive micro hydrogen producer (HMS/A@GE). **(B)** Representative TEM depicts of GE, HMS, and HMS/A@GE nanoparticles. **(C)** Body weight fluctuates in mice during the experimental phase. **(D)** Fasting blood glucose levels at the conclusion of the experiment. **(E)** Changes in blood glucose levels during the IPGTT experiment. **(F)** The area under the curve (AUC) of glucose in IPGTT. Adapted with permission from ref. 163 Copyrights 2025, Elsevier.

Building on this, Wang *et al*. 163 developed a biomimetic oral nano-hydrogen producer, termed HMS/A@GE, to address key challenges in type 2 diabetes (T2DM), specifically insulin resistance and pancreatic β-cell dysfunction (**Figure 12A**). HMS nanoparticles, modified with amino groups (HMS-NH2), were coated with ginger-derived exosomes (GE) to form the HMS/A@GE system. This formulation improves biocompatibility and regulates gut microbiota, releasing hydrogen gas in response to acidic conditions in the stomach. The released hydrogen reduces oxidative stress and inflammation in the pancreas and liver, while also enhancing the abundance of Lactobacilli species and promoting the release of indole derivatives such as indole-3-acetic acid (IAA). These compounds help restore intestinal mucosal barriers and regulate fat and carbohydrate metabolism by activating the AhR pathway through IL-22 release.

*In vivo* studies confirmed that HMS/A@GE significantly improved islet β-cell function and insulin resistance, contributing to better glucose metabolism and alleviating hepatic steatosis associated with T2DM. This dual therapeutic approach, which combines microecological and antioxidant treatments through hydrogen gas and ginger-derived exosomes, provides a more comprehensive solution to the complex pathophysiology of T2DM compared to traditional medications. Fresh ginger juice was separated from the exosomes by differential centrifugation, and the morphology of the prepared GE samples was visualized using transmission electron microscopy (TEM). The GE nanovesicles were found to be spherical, with an average diameter of approximately 200 nm, while HMS nanoparticles exhibited a consistent size of around 250 nm (**Figure 12B**).

Throughout the treatment, body weight was monitored, and while the A solution and HMS@GE nanoparticles caused a slight reduction in body weight, the saline-treated mice exhibited an increase in body weight, as shown in (**Figure 12C**). Notably, HMS/A@GE nanoparticles prevented the weight gain associated with a high-fat diet (HFD). Further, fasting blood glucose (FBG) levels were significantly lower in mice treated with HMS/A@GE (**Figure 12D**). Glucose tolerance tests (IPGTT) conducted one week before the end of the experiment revealed that glucose homeostasis was impaired in the T2DM mice treated with saline or HMS nanoparticles, but significantly improved in the HMS@GE and HMS/A@GE-treated mice. The area under the curve (AUC) of glucose levels showed that HMS/A@GE-treated diabetic mice had a lower AUC than those treated with saline (**Figures 12E-F**).

HMS/A@GE demonstrates exceptional efficacy in delivering hydrogen (H2), significantly reducing oxidative stress and inflammation in both the pancreas and liver. The released hydrogen synergizes with the ginger-derived exosomes (GE) to alter the metabolic profile and composition of the gut microbiota. This combined effect helps to maintain the integrity of the intestinal mucosal barrier, while also improving metabolic disorders. Notably, HMS/A@GE increased the abundance of Lactobacillus species and promoted the production of beneficial metabolites such as indole-3-acetic acid (IAA), which further activates the AhR/IL-22 signaling pathway. Importantly, no signs of toxicity were observed in the mice treated with HMS/A@GE. As a result, this approach offers a promising strategy to address key challenges in the management of AP.

#### 4.2.2 H_2_S therapy

Gases like nitric oxide (NO) and carbon monoxide (CO) have well-established roles in both physiological and pathological conditions. In recent years, attention has also turned to other naturally occurring gases, such as hydrogen sulfide (H_2_S), which has been shown to have potent vasodilatory effects, both *in vitro* and *in vivo*. This effect is likely mediated through the opening of vascular smooth muscle K^+^-ATP channels. Both cystathionine-γ-lyase (CSE) and cystathionine-β-synthase (CBS) use l-cysteine as a substrate to produce H_2_S, with CSE being the most prominent enzyme responsible for H_2_S production in the vasculature and heart.

AP, a common clinical condition, has been increasingly observed in recent years, often triggered by gallstones or excessive alcohol consumption. Regardless of the underlying cause, the activation of digestive enzymes within pancreatic acinar cells is considered a critical initiating event. This pancreatic injury induces a localized inflammatory response, which can progress to a systemic response. When detected systemically, this response can lead to leukocyte-mediated damage to distant organs, potentially resulting in multiple organ dysfunction syndrome (MODS). Inflammatory mediators play a crucial role in the pathogenesis of MODS in acute pancreatitis, which is a major contributor to mortality in this condition. Lung injury, clinically known as acute respiratory distress syndrome, is a significant component of MODS in acute pancreatitis.

Recent studies have highlighted that H_2_S may also play a role in various cardiovascular diseases, including septic and endotoxic shock, as well as pulmonary hypertension. Moreover, H_2_S interacts with other gaseous mediators like NO and CO. However, the potential involvement of H_2_S in inflammatory diseases such as acute pancreatitis remains underexplored. In one study by Bhatia *et al*. 164 the role of H_2_S in pancreatitis was investigated using an animal model. In this model, the researchers assessed the activity of H_2_S-synthesizing enzymes and the expression of CSE mRNA in rat pancreatic tissues. Additionally, they explored the effects of DL propargylglycine (PAG), an irreversible inhibitor of CSE, on the progression of pancreatitis and subsequent lung damage.

Although the exact mechanisms by which H_2_S acts as a pro-inflammatory mediator in acute pancreatitis are not fully elucidated, the findings from this study suggest that inhibiting H_2_S synthesis may offer a promising therapeutic strategy for managing acute pancreatitis and its systemic complications. Further research is required to assess the broader applicability of these inhibitors for treating other inflammatory conditions.

### 4.3 Gene therapy

Messenger RNA (mRNA)-based protein replacement therapies have shown great potential in treating various disorders, often requiring precise delivery mechanisms to ensure targeting of specific tissues or organs 165. While lipid nanoparticles (LNPs) are commonly used for systemic mRNA delivery, they generally target the liver due to its significant protein synthesis capabilities. However, achieving targeted mRNA delivery to extrahepatic tissues remains a challenge, with a risk of off-target effects 166. Pancreatitis refers to a group of conditions characterized by pancreatic dysfunction, often accompanied by damage to other organs and systems, typically due to autodigestion of pancreatic tissue. This disease is a leading cause of hospitalization for gastrointestinal issues, associated with high morbidity, mortality, and economic burden. AP recurrent acute pancreatitis, and chronic pancreatitis are all variations of this disease. Causes of pancreatitis include biliary tract and pancreatic duct issues, alcohol use, medications, infections, and immune responses. Current treatments are primarily supportive, highlighting the need for novel therapeutic strategies. Fibroblast growth factor 21 (FGF21) plays a critical role in regulating metabolic stress responses and is present in multiple organs, including the liver, pancreas, and adipose tissue. Studies have shown that FGF21-deficient mice exhibit increased susceptibility to pancreatitis, while genetic overexpression or recombinant FGF21 treatment provides protection in experimental models. The endocrine functions of FGF21 suggest it could be applied in mRNA-based therapies for pancreatitis without requiring targeted delivery to specific organs 167.

To explore this possibility, Liu *et al.* 168 developed a versatile lipopolymer nanoparticle (LPNP) system, named P6CIT-LPNP, that is adaptable for different delivery routes and exhibits an excellent safety profile. When FGF21 mRNA was encapsulated in P6CIT-LPNP (P6CIT-LPNP/mFGF21) and administered via subcutaneous (SC) injection, it led to localized translation at the injection site. Intravenous (IV) administration, however, facilitated greater transduction in the liver and spleen. In both cases, the produced FGF21 entered circulation and ultimately reached the pancreas. Interestingly, intraperitoneal (IP) injection targeted the pancreas more directly, leading to pancreatic FGF21 expression. Despite these differences, all three delivery methods resulted in therapeutic levels of pancreatic FGF21, providing equivalent therapeutic benefits. These results underscore that the endocrine properties of FGF21 allow its therapeutic effects to be independent of organ-specific targeting.

**Figure 13:**
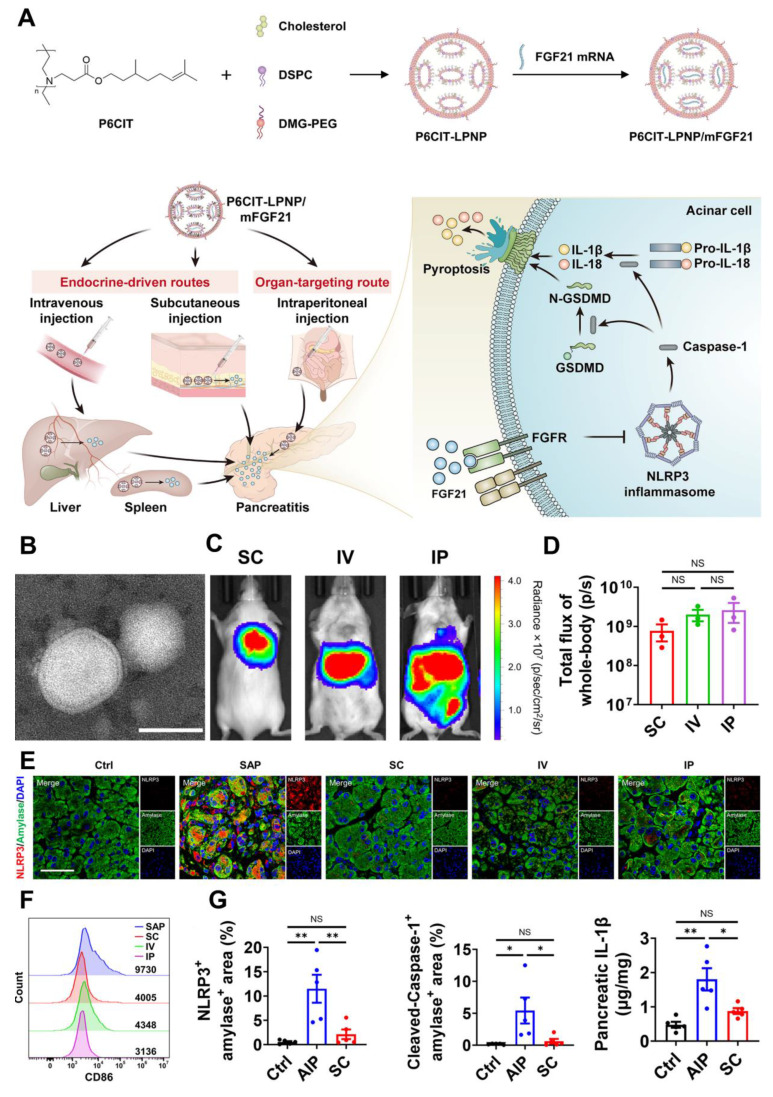
** (A)** A schematic illustration of the therapeutic mechanism of P6CIT-LPNP/mFGF21 administration in pancreatitis. **(B)** A typical picture of P6CIT-LPNP seen using TEM. **(C-D)** ICR mice received P6CIT-LPNP containing 2 μg of Luc mRNA via SC, IV, and IP injections. **(E)** SAP mice were treated with P6CIT-LPNP containing 5 μg of FGF21 mRNA via SC, IV, and IP routes at 2 hours after injection. **(F)** Representative flow cytometry pictures and measurement of the average fluorescence intensity of CD86 and M1 macrophages among living cells. **(G)** Representative illustrations of mIF staining and the percentage of NLRP3^+^amylase^+^ and cleaved-Caspase-1^+^amylase^+^ cells. Pancreatic IL-1β and IL-18 levels are shown with a 50 μm scale bar. Adapted with permission from ref. 168 Copyrights 2025, Elsevier.

FGF21 effectively reduced acinar cell pyroptosis by inhibiting the NLRP3/Caspase-1/gasdermin D (GSDMD) pathway (**Figure 13A**). Pyroptosis is a form of programmed cell death that is central to the pathology of pancreatitis. This cell death is marked by the formation of membrane pores, leading to cell rupture and the release of intracellular contents, which aggravates the inflammatory response. By inhibiting pyroptosis, FGF21 not only protects cells from death but also reduces the production of inflammatory mediators, making it an effective treatment for both severe acute pancreatitis (SAP) and autoimmune pancreatitis (AIP).

Transmission electron microscopy (TEM) imaging revealed that P6CIT-LPNP has a distinct spherical shape (**Figure 13B**). To investigate the biodistribution of P6CIT-LPNP, the team used SC, IV, and IP injection routes to deliver P6CIT-LPNP/mLuc to ICR mice. There were no significant differences in whole-body bioluminescence among the three injection routes (**Figures 13C-D**). In SAP mice, a significant increase in NLRP3^+^amylase^+^ and cleaved-Caspase-1^+^amylase^+^ acinar cells was observed, which was markedly reduced following treatment with P6CIT-LPNP/mFGF21 via all three injection methods. This suggests that FGF21 effectively reduces acinar cell pyroptosis in SAP (**Figures 13E**).

To evaluate the impact of P6CIT-LPNP/mFGF21 on the immune response, pancreatic single cells were isolated and analyzed using flow cytometry. The results showed a reduction in M1 macrophages and dendritic cells (DCs) in treated SAP mice compared to untreated controls, while M2 macrophages and natural killer (NK) cells remained largely unchanged (**Figure 13F**). In AIP mice, a marked increase in NLRP3^+^amylase^+^ and cleaved-Caspase-1^+^amylase^+^ acinar cells was observed, which was reversed by P6CIT-LPNP/mFGF21 injection, demonstrating that FGF21 mRNA treatment effectively suppresses acinar cell pyroptosis in AIP (**Figure 13G**).

To conclude, the administration of P6CIT-LPNP/mFGF21 through various injection methods effectively restored pancreatic FGF21 protein to therapeutic levels and demonstrated similar therapeutic outcomes. Additionally, FGF21 was able to suppress acinar cell pyroptosis, providing protection against both severe acute and autoimmune pancreatitis. These results highlight the potential of FGF21 mRNA replacement therapy for treating pancreatitis without the need for organ-specific targeting, and they underscore its promising applicability for future clinical use.

Gene therapy for pancreatitis faces significant challenges, particularly concerning the stability and scalability of the therapeutic approach. One of the primary issues with stability is the potential degradation of genetic material, which can undermine the long-term effectiveness of the therapy. Additionally, delivering genes to the pancreas in a targeted, efficient manner is complex, as gene delivery systems often experience rapid clearance or fail to precisely target pancreatic tissues. Scalability is another critical obstacle, as transitioning from laboratory-scale production to clinical-scale manufacturing presents challenges in both cost and consistency, especially when producing large quantities of high-quality gene therapies. To address these issues, several strategies have been proposed. Enhancing the stability of gene delivery vectors, such as using more robust, non-viral carriers like lipid nanoparticles or biodegradable polymers, could prevent the rapid degradation of genetic material. Moreover, improving the targeting mechanisms of gene delivery systems, such as utilizing tissue-specific promoters or incorporating advanced biomaterial-based carriers, could ensure more efficient and localized delivery to pancreatic cells. On the scalability front, developing more efficient production techniques, such as optimizing bioreactor systems and streamlining the manufacturing process, is essential to meet the demands of clinical application. These advancements are critical for overcoming the current barriers to the clinical adoption of gene therapy for pancreatitis 169.

Nanomedicine, gas therapy, and gene therapy offer distinct strategies for treating pancreatitis, each with its own strengths and challenges. Nanomedicine allows for targeted and controlled drug delivery, which can enhance therapeutic efficacy while minimizing side effects. However, issues like scaling up production, meeting regulatory requirements, and ensuring deep tissue penetration remain significant obstacles. Gas therapy, which works by modulating inflammation and oxidative stress, provides a non-invasive method that can have immediate therapeutic effects, but its short-lived nature may limit its long-term impact. Gene therapy presents a promising avenue for sustained treatment by delivering genetic material to alter cellular functions directly 170. Despite its potential, challenges like delivery efficiency, safety concerns, and complex clinical implementation need to be overcome. Each therapeutic approach differs in its targeting efficiency, administration methods, stage of clinical development, and ability to address the root causes of pancreatitis, making it essential to consider these factors when evaluating treatment options.

## 5. Specialty food therapy

The pancreas is a vital organ with two main functions: hormone secretion, including insulin, and the regulation of blood glucose levels 171. For academic and research purposes, the pancreas is often divided into exocrine and endocrine categories. However, these two functions are closely interrelated and together form a single organ. From a nutritional perspective, the exocrine function is more important, as it is involved in digestion and nutrient absorption. Disruptions in this function, such as those caused by pancreatic cancer (PC) or acute or chronic pancreatic damage, can lead to significant health challenges 172.

Malnutrition, resulting from impaired food intake or absorption, can severely affect body composition and compromise both physical and mental health 173. When diagnosing pancreatic disorders, and throughout the course of treatment, it is crucial to conduct nutritional assessments 174. The Global Leadership Initiative on Malnutrition advocates for a two-step process to assess malnutrition. The first step involves identifying patients who are at risk of malnutrition, followed by a second step where the diagnosis is confirmed, and the severity of the condition is evaluated 175.

Pablo *et al.* 176 explored the critical role of nutritional support in the management of pancreatic diseases, including AP), CP, and PC, all of which significantly impact the nutritional status and etabolic function of affected patients. The importance of early identification of malnutrition, plays a pivotal role in influencing disease outcomes and survival. In AP, where the body's inflammatory response and high metabolic demand contribute to catabolic stress, the authors recommended initiating enteral nutrition (EN) as soon as possible, with the use of nasogastric (NG) or nasojejunal (NJ) tubes being preferred. This approach not only provides essential nutrients but also has the added benefit of reducing gut permeability, preventing the translocation of bacteria, and minimizing the risk of infections. If EN was not feasible, they advocated for total parenteral nutrition (TPN) as a viable alternative to ensure adequate caloric intake and protein synthesis. The study further highlighted the high prevalence of pancreatic exocrine insufficiency (PEI) in these conditions, which results in malabsorption, fatigue, and weight loss due to insufficient production of digestive enzymes. For managing PEI, pancreatic enzyme replacement therapy (PERT) was considered essential, as it improves digestion, enhances nutrient absorption, and helps correct the nutritional deficits associated with these diseases. In PC, where the tumor burden and systemic inflammatory response worsen nutritional depletion, PERT was shown to significantly improve nutritional status, quality of life, and even survival outcomes when administered early in the disease course. The authors concluded that a multidisciplinary approach integrating nutritional screening, individualized dietary interventions, and the systematic use of PERT is essential for optimizing the clinical management of patients with pancreatic diseases, improving both short- and long-term outcomes. This approach helps address the complex interplay between malnutrition, inflammation, and disease progression in these patients.

The primary goals of medical nutrition therapy are to minimize meal-induced hyperglycemia and address or prevent chronic steatorrhea and malnutrition. Previous studies have shown that oral pancreatic enzyme replacement therapy can enhance insulin secretion and improve postprandial glucose control by restoring the secretion of incretin hormones. While a low-fat diet may alleviate symptoms of steatorrhea, it could potentially lead to an increased intake of carbohydrates, worsening postprandial hyperglycemia. To ensure optimal outcomes, it is essential for nutritionists to carefully adjust pancreatic enzyme dosage and timing, as well as dietary plans that include medium-chain triglycerides and supplements like fat-soluble vitamins, sulfur amino acids, folate, zinc, and other key micronutrients, all while managing the risk of bacterial overgrowth. Nutritionists, familiar with the specific challenges faced by these patients, should play an active role in their care.

Developing effective therapies for pancreatitis remains a complex task, largely due to the numerous biological barriers that drugs must overcome to reach their intended targets 177. Depending on the nature of the disease, its cellular environment, and the organs involved, therapeutic agents often face challenges in achieving sufficient bioavailability at the site of action. While standard treatment strategies typically focus on managing symptoms and providing supportive care, there is a growing emphasis on using small-molecule compounds to address the underlying disease mechanisms 178. These compounds are designed to target specific pathways involved in cellular stress, inflammation, and calcium dysregulation. For instance, pharmacological blockade of the ORAI1 calcium channel has shown potential in reducing acinar cell injury by preventing abnormal calcium influx, a key contributor to pancreatic inflammation 179-181. Simultaneously, plant-derived compounds many with roots in traditional Chinese medicine (TCM) have shown promise in modulating critical signaling pathways 182. Agents such as emodin, baicalein, and scutellarin help dampen inflammatory responses by interfering with molecular regulators like NF-κB, MAPK, and TLR4 183. Moreover, complex herbal mixtures such as the Qing-Yi and Da-Cheng-Qi decoctions appear to work through multiple mechanisms, including reinforcing the gut barrier, reducing proinflammatory cytokine release, and improving microvascular function in the pancreas 184-186. These insights highlight a promising therapeutic direction that combines targeted small-molecule approaches with the holistic, multi-target nature of traditional phytotherapy for the management of pancreatitis.

## 6. Living probiotic therapy

Probiotic-based treatments have shown potential in animal models of AP. However, their relevance in human illness remains uncertain. Four mechanisms by which probiotics may enhance human health are currently under investigation: (1) local and systemic immunomodulatory effects mediated by specific probiotic strains; (2) production of postbiotics, such as Poly-Unsaturated Fatty Acids (PUFAs), SCFAs, and glutamine, which influence intestinal homeostasis and regulate the enteric nervous system; (3) reversing inflammatory changes or strengthening the gut barrier through endogenous pathways, such as defensin production and the mucus layer 187,188. Rycther *et al*. 189 demonstrated that administering multispecies probiotics for two days before the onset of AP significantly corrected the impairment of intestinal barrier function that occurs during the late phase of AP in a mouse model. However, when delivered during the acute phase of AP, the same probiotic regimen failed to show equivalent benefits. Moreover, it highlighted the effectiveness of probiotics in treating AP that can vary depending on the stage of the disease. During the early phase of AP, some probiotic strains may not only fail to provide benefits but could potentially worsen the condition. This could be due to the inflammatory environment in the early stages of the disease, which might make certain probiotic strains counterproductive by exacerbating inflammation or disrupting gut microbiota. The study also questions whether using probiotics with specific, well-researched strains is more beneficial than relying on broad-spectrum, multi-species formulations. While broad-spectrum probiotics are commonly used for a variety of conditions, they may not be the most appropriate choice for early-stage AP, where more targeted probiotic strains may provide better therapeutic outcomes. Thus, the research suggests that selecting probiotics based on their specific actions and the phase of the disease may be a more effective approach than using generalized multi-strain products 190.

Further studies in mouse models examined the combination of probiotics and antibiotics in induced AP. Probiotics were found to reduce pathogenic bacterial translocation, and when combined with antibiotics, they reduced histopathological scores (e.g., edema, inflammatory infiltration, fat necrosis, parenchymal necrosis, and hemorrhage), along with oxidative indicators. These findings suggest that combining probiotics with antibiotics might be more effective in reducing pancreatic damage than using probiotics alone. In clinical trials, probiotic supplementation for AP has led to inconsistent results. Akyol *et al*. 191 conducted a randomized, double-blind trial to investigate whether probiotics could reduce hospital stay length in patients with moderate acute pancreatitis. Their findings suggest that probiotics may help reduce hospital admissions in patients with mild AP, as they were associated with faster resolution of abdominal discomfort and earlier transition to oral feeding than the placebo group. However, several important factors must be considered, such as the form of probiotics and their therapeutic variability.

## 7. Exosome therapy

Exosomes, which facilitate intercellular communication, play a vital role in both the detection and treatment of pancreatic diseases 192. These extracellular vesicles contain a diverse array of proteins, RNAs, and other bioactive molecules that influence gene expression and regulate critical processes such as inflammation, angiogenesis, and cellular protection after injury. Exosomes are implicated in various physiological and pathological mechanisms, including developmental biology, epigenetic regulation, immune system modulation, and tumor initiation and progression. In the context of pancreatic diseases, exosomes contribute to several stages of disease progression, impacting processes like apoptosis, immune modulation, angiogenesis, as well as the migration and proliferation of cells. Their role in these critical pathways makes exosomes a promising target for therapeutic interventions in pancreatic disorders. Changes in the quantity and composition of exosomes can indicate disruptions in pancreatic cell biology, potentially serving as a marker for disease. This makes exosomes a promising candidate for both diagnostic and therapeutic applications. Due to their high sensitivity and specificity, exosomes have been explored in the diagnosis and treatment of various cancers and inflammatory conditions, particularly for detecting tumors that are challenging to identify in their early stages through routine clinical evaluation. Additionally, modifications in specific exosomal components may provide insights into the progression and potential evolution of the disease, offering valuable information for personalized treatment strategies 193.

**Figure 14:**
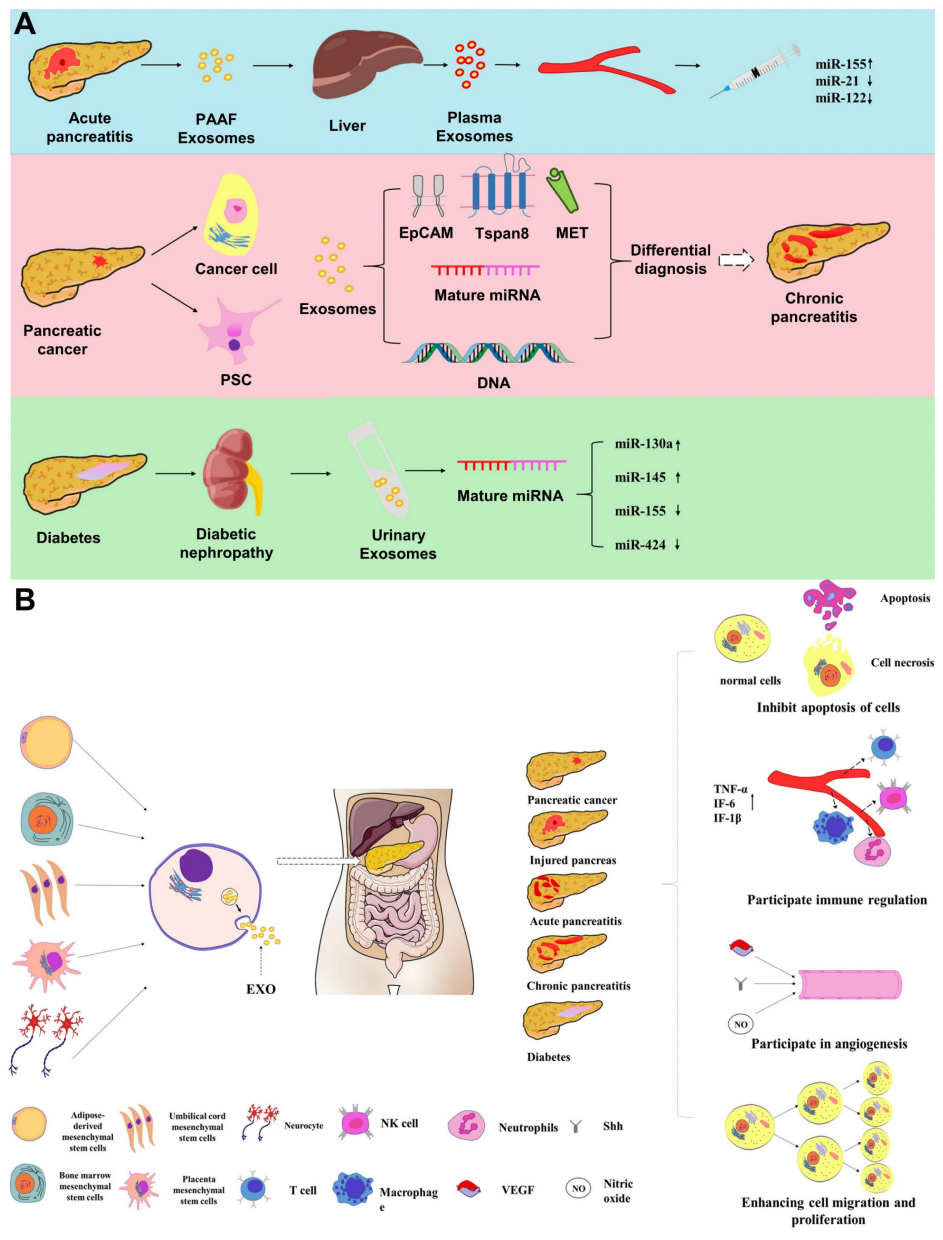
** (A)** Utilizing exosomes to diagnose pancreatic disorders. **(B)** Different cell types' exosomes curing pancreatic disorders using a variety of processes, including as immunological modulation, angiogenesis, cell migration, proliferation, and apoptosis. Adapted with permission from ref. 195 Copyrights 2022, Springer Nature.

Tracking changes in exosome composition can significantly aid in clinical diagnosis and treatment, providing physicians with valuable insights into a patient's condition. This real-time information allows for more accurate and timely adjustments to treatment plans. Consequently, exosomes are gaining recognition as a novel tool for obtaining disease-specific data, as they reflect underlying pathophysiological processes. In summary, research highlights the growing potential of exosomes for various applications in managing pancreatic diseases, offering promising avenues for both diagnosis and therapy 194. Numerous studies are currently exploring the role of microRNAs (miRNAs) carried by exosomes in diagnosing systemic inflammation induced by AP (**Figure 14A**) 195. In a recent study, exosomal miRNAs were extracted and analyzed using microarray technology, revealing that 30 miRNAs were significantly elevated in AP cases. Additionally, the research highlighted that pancreatic acinar cell influence macrophage activation through the secretion of exosomes containing these specific miRNAs. In addition, plasma-derived exosomes exhibited greater pro-inflammatory effects on macrophages compared to those from pancreatic ascitic fluid (PAAF). The miRNA expression profile in the plasma of AP patients has been characterized, and ongoing studies are working to validate these findings with a larger sample size. The detection of exosomes containing specific miRNAs holds promising potential as a diagnostic tool for acute pancreatitis.

Apoptosis is characterized by mitochondrial dysfunction and the activation of apoptotic signaling pathways. Exosomes play a role in inducing apoptosis through various signaling pathways, including Wnt/β-catenin, NF-κB, and Akt/PI3K. The process of exosome-mediated apoptosis is closely associated with the initiation and progression of pancreatic diseases (**Figure 14B**). Bone marrow-derived mesenchymal stem cells (BMSCs) secrete exosomes that regulate inflammatory cytokine production and reduce inflammatory cell infiltration through the NF-κB signaling pathway. This leads to a reduction in serum lipase and amylase levels, suggesting repair and regeneration of necrotic pancreatic tissue in AP. Exosomes influence pancreatitis by modulating various signaling pathways and may also have an impact on other pancreatic conditions, such as diabetes and pancreatic cancer (PaCa).

Through four primary mechanisms, exosomes and their bioactive compounds affect cell apoptosis during the onset and progression of pancreatic diseases by controlling various signaling pathways, immune cell status, and the upregulation or inhibition of related molecule expression. Compared to their parent cells, exosomes are smaller, simpler, easier to make and store, and do not carry the same danger of developing into tumors. Nonetheless, exosomes have a variety of functions at different phases of pancreatic disorders, and the majority of recent studies have concentrated on the intricate signals that underlie exosome-mediated tissue remodeling and functional restoration. The complicated pathophysiology and unclear etiology of pancreatic disorders have drawn scientific attention and pose a severe hazard to human health. Endocytosis creates exosomes, which can be released into extracellular vesicles to aid in cell communication. By controlling cell death, taking part in immunological regulation, and encouraging angiogenesis, cell proliferation, and cell migration, exosomes contribute to the incidence and progression of illnesses. Furthermore, research is now concentrating on using exosomes to diagnose pancreatic disorders.

## 8. Treatment of dual pancreatitis and its concomitant diseases

Severe acute and chronic pancreatitis often contribute to the development of other serious conditions, including ductal adenocarcinoma and lung injury 196. These concomitant diseases share common underlying mechanisms such as chronic inflammation, oxidative stress, and immune system disturbances, which drive the progression of the diseases in parallel. Inflammatory pathways, particularly those involving NF-κB and cytokine release, amplify pancreatic damage and induce systemic effects that impact other organs, including the lungs 197. Additionally, the fibrotic changes that occur in the pancreas extend to the lung tissue, leading to pulmonary fibrosis and compromised lung function. The activation of various cell death pathways, such as apoptosis, necroptosis, and pyroptosis, further exacerbates tissue destruction in both the pancreas and the lungs 198. Moreover, the same molecular processes that facilitate tumor angiogenesis and tumor growth in pancreatic cancer also contribute to vascular changes in the lungs, worsening lung injury. Given the interconnected nature of these concomitant diseases, a comprehensive treatment approach targeting the shared mechanisms is essential for improving patient outcomes.

Inflammatory mediators such as NF-κB and NLRP3 inflammasomes play a significant role in pancreatic β-cell dysfunction and fibrosis, which accelerate the development of pancreatogenic diabetes. The inflammatory cytokines IL-6, TNF-α, and IL-1β also disrupt the balance between bone formation and resorption, leading to conditions like osteopenia and osteoporosis. Cardiovascular risk is heightened due to chronic inflammation, which contributes to endothelial dysfunction and promotes atherosclerosis through signaling pathways like NF-κB and JAK-STAT. Additionally, the inflammatory response in CP compromises the integrity of the gut barrier, increasing intestinal permeability and triggering further systemic inflammation. Targeting these inflammatory pathways with specific therapies could offer a comprehensive approach to treating both the underlying pancreatitis and its associated comorbidities, ultimately improving patient quality of life and long-term outcomes. Herein, we summarized a table for the key comorbidities, mutual mechanisms and their impact on pancreatitis **(Table 2)** 199-202**.**

### 8.1 Treatment of dual ductal adenocarcinoma and pancreatitis

Pancreatic ductal adenocarcinoma (PDAC), which arises from the exocrine pancreas, accounts for about 85% of all malignant pancreatic tumors, making it one of the deadliest digestive system cancers 203. One Its mortality and incidence are still on the rise worldwide. For example, estimates in the European Union (EU) indicate that by 2025, about 111,500 people will die from pancreatic cancer, a nearly 50% increase over 2010 mortality rates, making pancreatic cancer the third most common cause of cancer-related deaths, after lung and colorectal cancers 204. A substantial public health concern exists in China as a result of increased incidence and mortality rates brought on by both improved living standards and shifting dietary patterns. The 5-year survival rate is still below 10% 205.

**Figure 15:**
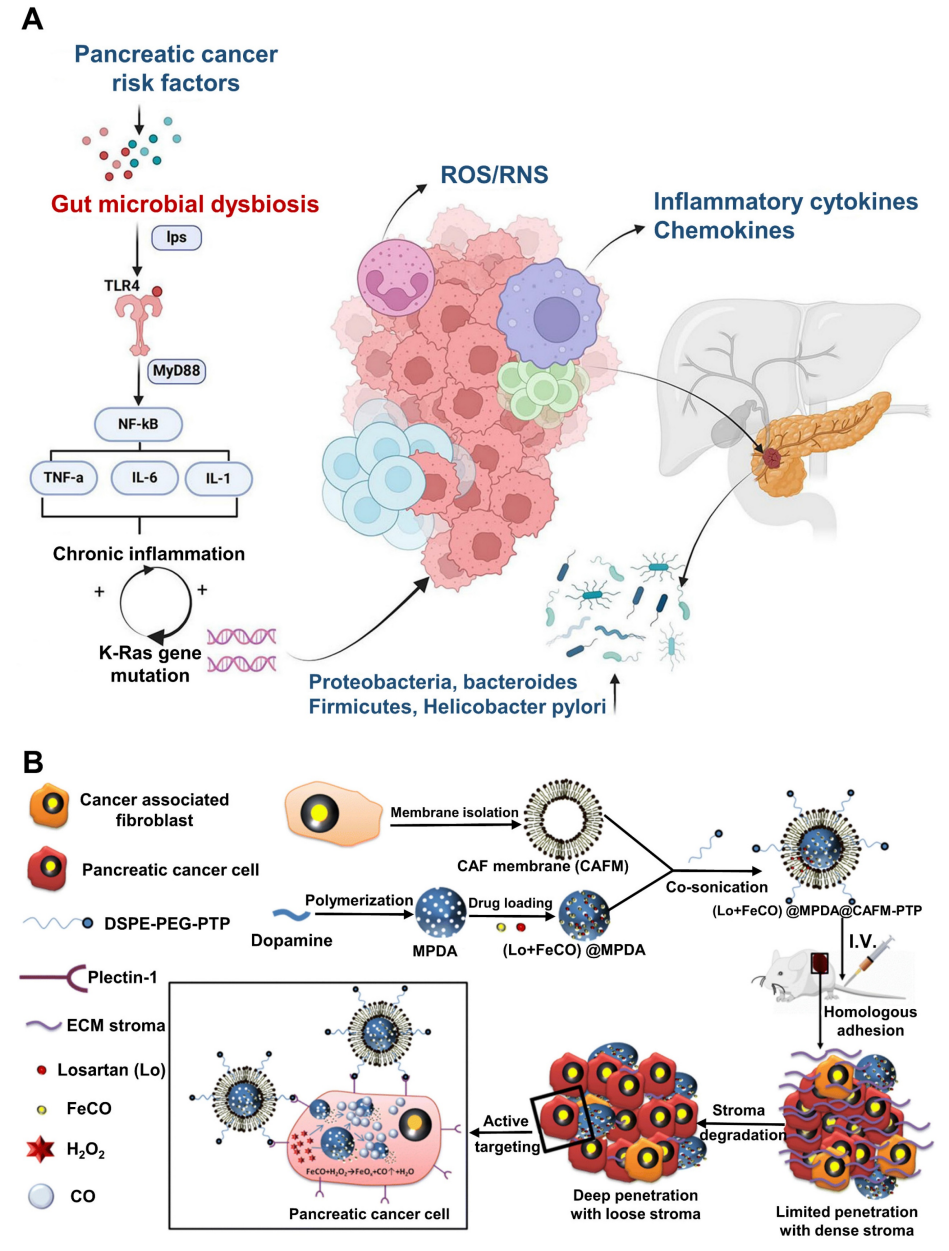
** (A)** The role of gut-microbiota-derived metabolites in the etiology and progression of pancreatic cancer. 203 **(B)** Diagrammatic representation of the biomimetic dual-targeting nanomedicine preparation (Lo + FeCO)@MPDA@CAFM-PTP and its potential use in treating pancreatic cancer. Adapted with permission from ref. 208 Copyrights 2025, Royal Society of Chemistry.

In addition to clinical difficulties, new data suggests a complex relationship between gut microbiota and pancreatic cancer development. About 100 trillion bacteria live in the human gastrointestinal system, generating a complex microecological network that is vital to innate immunity, intestinal homeostasis, and the integrity of the epithelial barrier 206. Pancreatic cancer has been associated with disruptions in this network, which are marked by an increase in opportunistic pathogens and a decrease in helpful microorganisms 207. The tumor microenvironment may change as a result of persistent immunological reactions and ongoing inflammation brought on by dysbiosis. By changing metabolic pathways, inducing pro-inflammatory signals, and suppressing immune cell differentiation, microbial metabolites that enter the pancreas through the mesenteric veins may accelerate tumor growth and metastasis. Zhou *et al*. 208 demonstrated that the gut microbiota plays a crucial role in the etiology and development of pancreatic cancer, despite the fact that the exact processes are still unclear (**Figure 15A**).

Nanoparticles can boost tumor tissue penetration and therapy efficacy, and this has been extensively researched. Among these, mesoporous polydopamine (MPDA) stands out as a bioinspired nanoparticle that is polymerized by dopamine molecules spontaneously under alkaline circumstances. MPDA has a number of special qualities that set it apart from other nanoparticles, such as high biocompatibility and biodegradability, ease of synthesis, pH responsiveness, etc. 13, 30, and 36 In recent years, MPDA has received a lot of attention in biological application research because of these qualities. Researchers have recently looked into it in great detail because of its porous structure and high drug-loading capacity, which make it a promising choice for smart drug delivery systems. As foreign materials, nanoparticles must overcome a number of obstacles, including as early immune system identification, rapid removal from the bloodstream, and inadequate accumulation at tumor locations. The biocompatibility of nanoparticles can be improved using a biomimetic technique that uses cell membrane coating to conceal them. This method enhances the efficacy of nanoparticles in biomedical applications by giving them the intrinsic characteristics and capabilities of cell membranes.

A biomimetic multifunctional nanomedicine was developed that can kill or inhibit PC cells by releasing CO, degrading the TME stroma, and targeting both CAF and PC cells. To create (Lo + FeCO)@MPDA nanoparticles, we first added both FeCO and Lo to MPDA nanoparticles. Then, in order to create (Lo + FeCO)@MPDA@CAFM nanoparticles, we isolated CAF cell membrane (CAFM) nano nanovesicles and coated them on the surface of the nanoparticles. The (Lo + FeCO)@MPDA@CAFM-PTP nanomedicine was then created by altering the surface of (Lo + FeCO)@MPDA@CAFM with the targeted molecule PTP. We predict that the (Lo + FeCO)@MPDA@CAFM-PTP nanomedicine will initially target the TME's CAF cells via homologous adhesion between CAFM and CAF cells in order to treat PC cancers. MPDA will then break down and release Lo, which will reduce the TME stroma and solid stress because of PC's acidic TME and MPDA's sensitivity to pH. As the solid stress decreases, the solid PC tumor becomes less rigid, which makes it possible for more (Lo + FeCO)@MPDA@CAFM-PTP nanomedicine to diffuse deeper into the tissue and reach PC cells. The (Lo + FeCO)@MPDA@CAFM-PTP nanomedicine to be internalized by PC cells through the interaction between PTP and plectin-1, which is overexpressed on PC cells. In the acidic microenvironment of PC cells, FeCO is released, which then reacts with the intracellularly produced ROS to release CO, which kills PC tumor cells (**Figure 15B**).

The creation of the (Lo + FeCO)@MPDA@CAFM-PTP nanomedicine, successfully targets PC tumors, destroys the tumor matrix, and kills cancer cells. These effects are explained by plectin-1 identification, CAF cell membrane-homologous adhesion, Lo matrix disintegration, and CO's ability to inhibit cancer cells. The findings showed that compared to other nanoformulations, the (Lo + FeCO)@MPDA@CAFM-PTP nanomedicine's targeting capability and anti-cancer effects were noticeably stronger. As a possible therapy approach for additional solid tumors, this nanomedicine shows promise.

### 8.2 Treatment of dual acute pancreatitis and acute lung injury

Patients with SAP who develop acute lung injury (ALI) face significantly increased rates of mortality, cardiovascular failure, and renal failure 209. ALI is one of the most serious complications of SAP, with early mortality (within the first week of onset) reaching as high as 70%. Consequently, preventing or treating ALI is essential for reducing the mortality associated with SAP 210. Various factors, such as microcirculation disturbances, activation of pro-inflammatory immune cells, toxins derived from pathogens, and cytokine imbalance, are believed to play a role in the onset of SAP-ALI 211. A primary route for these factors to reach the lungs is through the mesenteric lymphatic circulation. In animal models of acute pancreatitis, blocking mesenteric lymph flow has been shown to prevent the development of ALI, but this strategy has not been successful in human clinical trials due to the complexity of human physiology. As a result, the current treatment approach for SAP-ALI remains limited to supportive care and fluid management 212.

A deeper understanding of the underlying pathophysiological mechanisms driving SAP-ALI is essential 213. Developing therapies that target these mechanisms could significantly improve the prognosis for SAP patients. One critical structure for maintaining lung tissue homeostasis is the alveolar-capillary barrier, composed of pulmonary microvascular endothelial cells (PMVECs), fibroblasts, alveolar macrophages, and epithelial cells 214. ALI is characterized by damage to this barrier, leading to increased permeability of the alveoli. This allows the infiltration of water, red blood cells, pro-inflammatory molecules (such as tumor necrosis factor-α [TNFα] and ROS), and immune cells like neutrophils and monocytes, which exacerbates inflammation and lung dysfunction. PMVECs, which line the pulmonary circulatory system, form a tightly regulated barrier that prevents the free passage of solutes, pathogens, and immune cells between the microvessels and lung interstitial space. Disruption of this barrier through apoptosis, necrosis, tight junction breakdown, or abnormal cell proliferation contributes to the development of ALI 215.

Dysregulation of endogenous cytokines, such as TNFα and IL-6, plays a key role in promoting ALI. Restoring the integrity of the PMVEC barrier or preventing its breakdown may help mitigate this condition 216. For example, blocking ROS formation in animal models of sepsis-induced ALI has been shown to enhance the expression of tight junction proteins like occludin and zonula occludens-1 (ZO-1) in endothelial cells, thereby preserving barrier function. Extracellular vesicles (EVs), small vesicles that carry bioactive materials such as proteins, lipids, and nucleic acids, have the ability to influence various physiological processes and disease progression 217.

However, the exact role of SAP-EVs in targeting PMVECs and mediating SAP-ALI remains unclear, especially since PMVECs are among the first cells to be affected in SAP-ALI. Certain tissues, including the lungs, can selectively absorb EVs via surface molecules such as integrins. For example, tumor-derived EVs that express integrins α6β4 and α6β1 are known to promote lung metastasis by binding to specific lung cell types, such as fibroblasts positive for S100A4 and epithelial cells expressing surfactant protein C (SPC). In our study, we found that certain integrins were enriched on SAP-EVs isolated from animal models of SAP and MAP. Given that integrins are involved in regulating the cytoskeletal structure, barrier function, angiogenesis, and inflammatory responses of PMVECs, we hypothesize that the enhanced expression of these integrins on SAP-EVs may enable these vesicles to target PMVECs, thereby contributing to the development of ALI during SAP. Identifying the surface proteins of SAP-EVs that promote their accumulation in the lungs could provide valuable insights into the pathogenesis of SAP-ALI and help guide the development of therapeutic strategies aimed at mitigating this serious complication 218.

To address the role of extracellular vesicles (EVs) in SAP-induced ALI, Hu *et al*. 219 demonstrated that the accumulation of SAP-derived EVs in lung tissue occurs through an integrin-dependent mechanism. Healthy mice injected with SAP-EVs showed lung tissue damage and inflammatory responses consistent with ALI. This accumulation was significantly reduced when SAP-EVs were pre-incubated with the integrin antagonist peptide HYD-1, or when EVs engineered to overexpress ITGAM or ITG2B integrins that are highly expressed on SAP-EVs were injected. These results suggest that targeting the accumulation of SAP-EVs in the lung by utilizing EVs or particles with high integrin expression could offer a therapeutic approach to improve the prognosis of SAP-ALI.

The nuclear receptor subfamily 4 group A member 3 (Nr4a3), also known as neuron-derived orphan receptor 1, is an orphan nuclear receptor that is widely expressed in several tissues, including the pancreas and pulmonary smooth muscle cells. Nr4a3 is activated as part of an early stress response and is associated with various biological processes, including inflammation and apoptosis. Despite these known associations, the precise role of Nr4a3 in SAP and SAP-related ALI remains unclear. Previous studies have shown that inhibiting histone deacetylases can increase Nr4a3 transcription, and that Sirtuin 1 (Sirt1), an NAD^+^-dependent deacetylase, functions as a transcriptional repressor by deacetylating histones and promoting chromatin condensation. The expression of Sirt1 is linked to SAP progression, with lower levels observed in the serum of SAP patients. Moreover, activating Sirt1 expression has been shown to reduce lung injury due to inflammation and apoptosis, and elevated Sirt1 levels significantly reduce SAP-associated ALI. These findings suggest a potential link between Nr4a3 and Sirt1 in the context of SAP-associated ALI.

**Figure 16:**
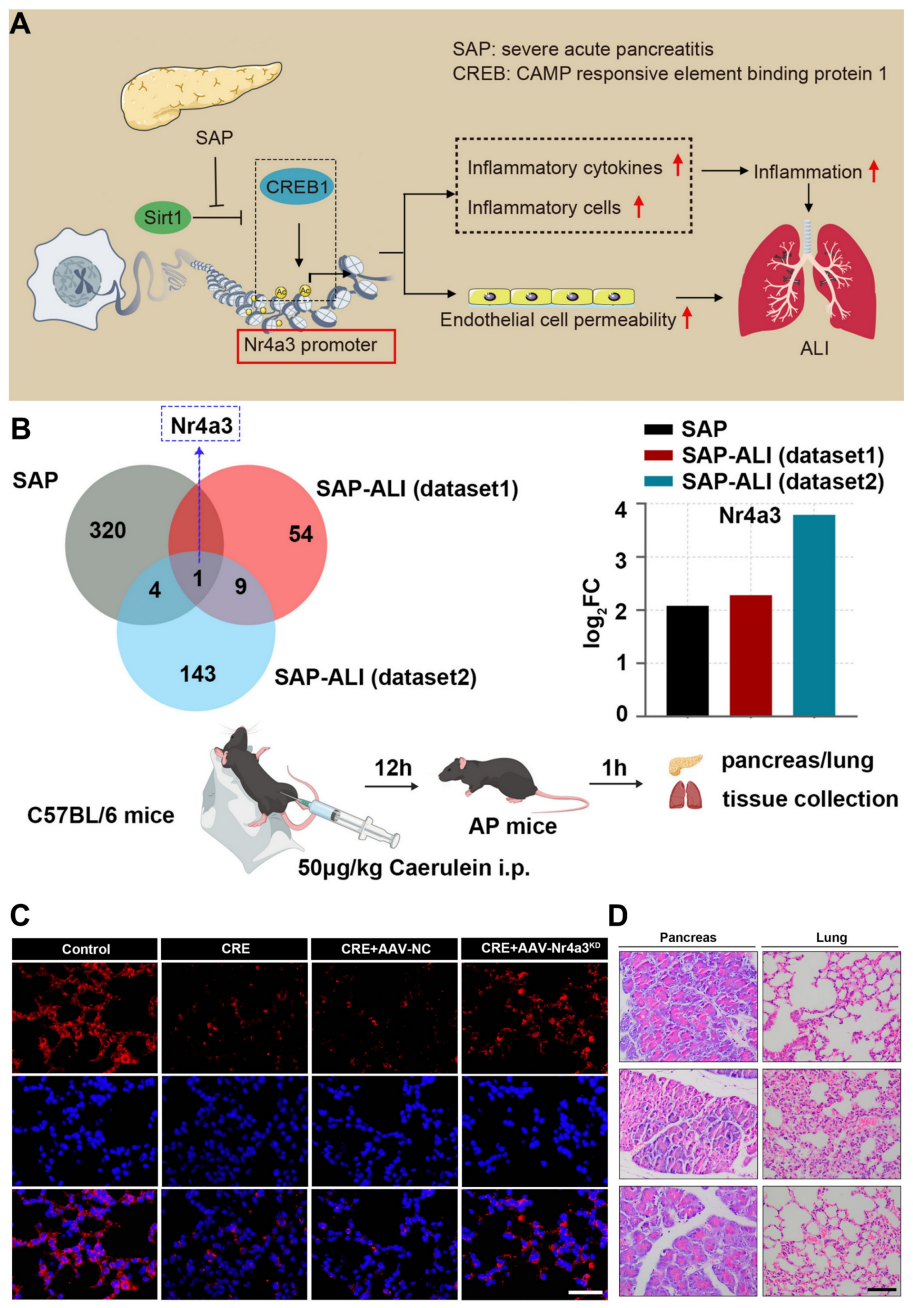
** (A)** A schematic of how Nr4a3 inhibition alleviates SAP-associated ALI. **(B)** A Venn diagram illustrating the relationship between Nr4a3 expression and differentially expressed genes in lung damage or pancreatitis, as well as the schematic diagram of the establishment of an acute pancreatitis (AP) mice model. **(C)** Images showing VE-cadherin immunostaining in mice's lung tissues after various treatments. **(D)** Examples of H&E-stained lung and pancreatic tissues in mice from several groups (Scale bars: 100 μm). Adapted with permission from ref. 220 Copyrights 2025, John Wiley and Sons.

To further investigate this relationship, Dou *et al.* 220 used a CRE-induced acute pancreatitis (AP) mouse model and a TNF-α-induced human pulmonary microvascular endothelial cell (hPMVEC) model to study the involvement of Nr4a3 and Sirt1 in SAP-related ALI. They explored the relationship between Nr4a3 and Sirt1 *in vitro* and *in vivo* and the impact of Sirt1 on Nr4a3 in regulating SAP-associated ALI (**Figure 16A**). Transcriptome data from both pancreatic and lung tissues of SAP and SAP-related ALI *in vivo* models identified a significant increase in Nr4a3 expression (**Figure 16B**), suggesting that Nr4a3 may play a role in the development of SAP or SAP-associated ALI. In the CRE-induced AP mouse model, Nr4a3 knockdown resulted in a significant restoration of VE-cadherin expression in lung tissue, which was reduced in CRE-induced animals (**Figure 16C**). This finding indicates that Nr4a3 knockdown may protect the pulmonary endothelial barrier from CRE-induced damage. Further, Nr4a3 knockdown alleviated tissue damage in both lung and pancreatic tissues in the CRE-induced AP model, whereas Sirt1 inhibition exacerbated the damage (**Figure 16D**). Histone acetylation, which regulates Nr4a3 transcription, was enhanced by histone deacetylase inhibition, making it easier for CREB to bind to the Nr4a3 promoter and activate its transcription. Sirt1, a histone and non-histone deacetylase, regulates a variety of biological functions, including gene transcription.

Overall, the *in vitro* and *in vivo* results demonstrate that Nr4a3 knockdown reduces inflammation and damage to the pulmonary endothelial barrier, thus alleviating SAP-associated ALI. By inhibiting Nr4a3 transcription, Sirt1 reduces CRE-induced ALI in AP mice, highlighting the Sirt1/Nr4a3 axis as a potential therapeutic target for SAP-related ALI. These findings underscore the involvement of this axis in SAP-associated ALI and offer a promising avenue for future therapeutic strategies.

### 8.3 Treatment of dual pancreatitis and diabetes

Pancreatogenic diabetes, also known as Type 3C diabetes or diabetes mellitus (DM associated with exocrine pancreatic disorders, can be classified into two categories 221. Both benign and malignant conditions affecting the exocrine pancreas are associated with pancreatogenic diabetes, which has a unique pathogenesis. Earlier studies indicated that pancreatogenic diabetes accounted for 0.5% to 1.15% of all diabetes cases in North America, but this percentage ranged from 15% to 20% in Southeast Asia, where tropical pancreatitis is more common. A recent study suggests that 5% of all diabetes cases in Germany are pancreatogenic, which raises the possibility that its prevalence in North America may be underreported 222. Chronic pancreatitis (CP) is responsible for over 80% of pancreatogenic diabetes cases. CP is a progressive degenerative disorder that affects both the exocrine and endocrine functions of the pancreas. According to various cohort studies, diabetes develops in 26-80% of CP patients, and within 25 years of alcoholic CP's clinical onset, up to 83% of patients will develop diabetes, with over half of them eventually requiring insulin. Risk factors for CP-related diabetes include early-onset CP, pancreatic calcification, a history of distal pancreatectomy, concomitant liver cirrhosis, smoking, and other typical type 2 diabetes risk factors. Contrary to earlier reports, the incidence of diabetic retinopathy and other microvascular and macrovascular complications in CP-associated diabetes (CP-DM) appears to be similar to that seen in type 1 and type 2 diabetes 223,224.

Like other forms of diabetes, CP-DM is primarily diagnosed based on clinical symptoms and biochemical testing. Exocrine pancreatic insufficiency is common among CP-DM patients. In catabolic states, overt hyperglycemia may present as rapid weight loss, polyuria, polydipsia, and polyphagia. Since patients with CP often have mild-to-moderate hyperglycemia that may be asymptomatic, they are at high risk for metabolic dysfunction, making regular screening for diabetes or glucose intolerance essential. Screening should involve periodic measurement of hemoglobin A1C and fasting glucose, ideally once a year 225. To distinguish CP-DM from type 1 and type 2 diabetes, Ewald *et al*. 226 proposed diagnostic criteria that include confirming CP and identifying signs of dysfunction in both the endocrine and exocrine functions of the pancreas. In order to differentiate CP-DM from type 2 or early-onset type 1 diabetes, the postprandial (PP) response to insulin-induced hypoglycemia, secretin infusion, or mixed-meal testing may be considered. Functional beta-cell mass can be assessed using serum C-peptide levels during oral glucose tolerance tests, mixed-meal testing, or glucose-potentiated arginine testing after confirming the diagnosis. Due to its complex nature, managing CP-DM requires a multidisciplinary approach that takes into account clinical presentation, the extent of pancreatic insufficiency, the mechanisms of anti-diabetic medications, patient adherence, and lifestyle adjustments.

New therapeutic strategies, such as dual insulin-glucagon delivery systems and pancreatic polypeptide analogs, show promise, though concerns regarding their safety and efficacy remain. As islet cell therapies continue to evolve, there is hope that these innovations will improve clinical outcomes and potentially alter the course of diabetes in CP patients.

### 8.4 Treatment of dual pancreatitis and bone disease

Osteoporosis is characterized by reduced bone mass and structural deterioration of bone tissue, leading to increased bone fragility and a higher risk of fractures 227. Around 25% of patients with chronic pancreatitis (CP) are predicted to develop osteoporosis, and nearly two-thirds are affected by either osteoporosis or osteopenia, according to a meta-analysis 228. These bone-related issues often go unnoticed but contribute significantly to the morbidity in CP patients. As per current European guidelines, preventive measures should be adopted by all CP patients. Those with confirmed low bone mineral density (BMD) should undergo BMD assessments every two years using dual-energy X-ray absorptiometry (DXA). However, routine clinical practice often overlooks bone health evaluations 229. Studies investigating the clinical significance of low-trauma fractures in CP patients have shown a 10-year fracture rate of 4.8%, comparable to that seen in patients who have undergone gastrectomy or suffer from high-risk conditions such as liver cirrhosis, inflammatory bowel disease, and celiac disease. Many studies on this topic have been hindered by small sample sizes that are inadequate for subgroup analysis and the diversity of data collected 230-232.

Metabolic bone disease is recognized as an extra-intestinal manifestation of inflammatory bowel disease (IBD). Harbord *et al*. 233 outlined the contributing factors for these symptoms in their European consensus guidelines. A meta-analysis conducted by Szafors *et al*. 234 examined fracture risks in IBD patients, revealing that individuals with IBD face a significantly higher risk of fractures compared to healthy controls (relative risk of 2.26). Specifically, vertebral fractures were much more common in IBD patients (odds ratio of 2.26), while fractures at other sites were not as prevalent. Since bone mineral density (BMD) is a key predictor of osteoporotic fractures, the analysis also showed that IBD patients typically had lower average BMD compared to controls, which has clinical significance. Patients with chronic pancreatitis (CP) may have additional risk factors for secondary osteoporosis, such as advancing age, low body mass index (BMI) due to food aversion, maldigestion caused by exocrine pancreatic insufficiency (EPI) leading to vitamin D deficiency, and lifestyle factors like smoking and alcohol consumption.

The goal of bone health recommendations for patients with cerebral palsy (CP) is to prevent fractures associated with osteoporosis, thereby reducing morbidity and improving quality of life 235. Osteoporosis can be classified into two main types: primary osteoporosis, which includes postmenopausal and age-related forms, and secondary osteoporosis, which may result from conditions like malabsorption, hyperparathyroidism, and certain medications. Various gastrointestinal disorders are linked to bone loss, including Crohn's disease (CD), inflammatory bowel disease (IBD), cirrhosis, post-gastrectomy conditions, and CP. Osteoporosis often remains asymptomatic until a fracture occurs, highlighting the need for early screening in at-risk patients 236.

Screening aims to assess bone mineral density (BMD), often represented as a "T Score." This score measures the number of standard deviations a person's BMD deviates from the average value of young, healthy individuals 237. A T score above -1.0 SD indicates normal BMD, a score between -1.0 and -2.5 SD suggests osteopenia, and a score below -2.5 SD points to osteoporosis. Given that approximately two-thirds of CP patients are affected by bone disease, early screening for CP-related osteopathy, including osteopenia and osteoporosis, is essential 238. A primary approach should include using pancreatic enzyme replacement therapy (PERT) to address malabsorption due to exocrine pancreatic insufficiency (EPI) 239,240. This, along with efforts to improve body mass index (BMI), should help enhance calcium absorption, address vitamin D deficiencies, and support overall nutritional health. In conclusion, osteopathy is common in CP patients, significantly contributes to morbidity, and requires regular screening and treatment. Managing the condition through PERT and fat-soluble vitamin supplements can help prevent further complications.

### 8.5 Treatment of dual pancreatitis and cardiovascular disease

In SAP, organ failure is the leading cause of premature mortality. While cardiac injury (CI) is rare, acute kidney injury (AKI) and ALI are common and significantly contribute to the high mortality rate 241,242. Various studies have revealed notable ultrastructural changes in the heart during SAP, including myocardial edema, cardiac hypertrophy, and collagen deposition in the myocardial interstitial space. Clinically, patients with SAP often exhibit abnormal cardiac function and electrocardiogram (ECG) findings 243. In some cases, complications such as pericardial tamponade and acute myocardial infarction (AMI) have been observed, sometimes progressing to stress-induced cardiomyopathy. Despite these findings, the mechanisms behind SAP-associated cardiac injury (SACI) are not fully understood, and there is currently no standardized treatment or comprehensive therapeutic approach. This article aims to explore recent research on the pathogenesis, potential biomarkers, and treatment options for SACI 244.

**Figure 17:**
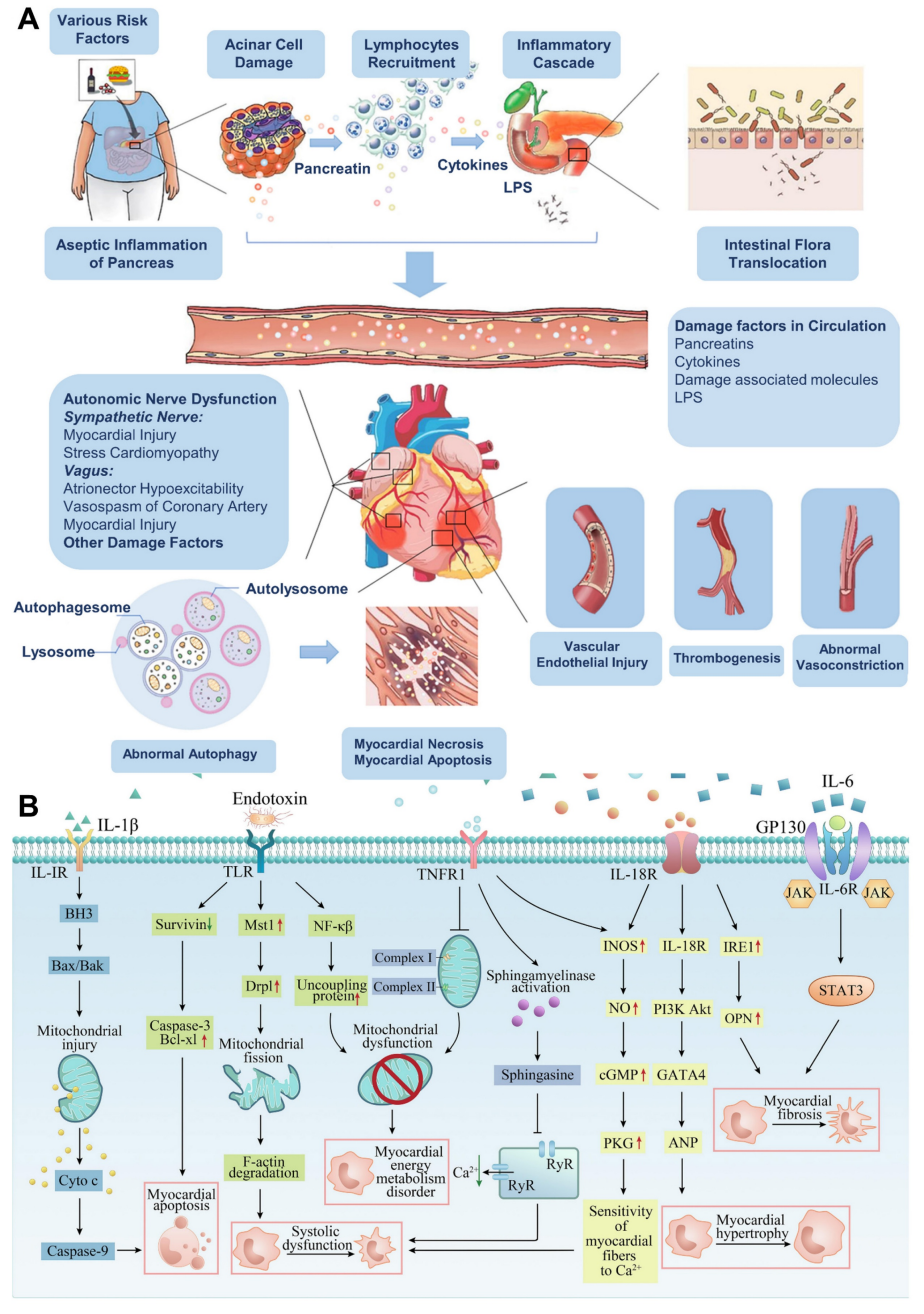
** (A)** Pathophysiological processes affecting how SAP cardiac injury develops. **(B)** The precise mechanisms via which inflammation-related variables lead to myocardial damage and cardiac failure. Adapted with permission from ref. 245 Copyrights 2025, Taylor & Francis.

The pathophysiology of SACI is complex, involving inflammatory damage across multiple organs. Luo *et al*. 245 identified three key processes that contribute to the development of SACI (**Figure 17A**): 1) In the early stages of acute pancreatitis (AP), damage to acinar cells and the subsequent release of trypsin trigger local pancreatic inflammation. This inflammation activates a cascade of mediators, leading to systemic inflammatory response syndrome (SIRS) and setting the stage for organ failure, including cardiac injury. 2) The intestinal barrier becomes compromised, allowing endotoxins to enter the bloodstream, further exacerbating pancreatic necrosis and spreading damage to extra-pancreatic organs, such as the heart. 3) SAP also leads to damage of both the nerve and vascular structures associated with the heart. Studies worldwide have shown that distant organ failure in SAP is closely linked to SIRS. Myocardial injury in AP is primarily caused by an imbalance in homeostasis, triggered by excessive trypsin, pro-inflammatory cytokines (e.g., TNF-α, IL-1β, IL-6, IL-18), and other inflammatory mediators (e.g., NO, ROS, HMGB1). These factors activate complex signaling pathways that collectively result in abnormal electrical activity in cardiomyocytes, mitochondrial dysfunction, disturbances in energy metabolism, systolic myocardial dysfunction, myocardial hypertrophy, fibrosis, and apoptosis (**Figure 17B**).

Damage-associated molecular patterns (DAMPs) and pathogen-associated molecular patterns (PAMPs), such as trypsin, inflammatory cytokines, and endotoxins, are released from the pancreas and intestines. These molecules contribute to endothelial cell damage, abnormal autophagy, and dysfunction of the autonomic nervous system, which in turn leads to both direct and indirect cardiac damage through vascular and neural pathways, and even within cardiomyocytes themselves. While the molecular mechanisms driving SAP-induced cardiac dysfunction are still poorly understood, research into sepsis-related cardiac injury provides some insights and guidance. The "pancreas-heart axis" encompasses a variety of factors, including trypsin, inflammatory mediators, reactive oxygen species (ROS), endotoxins, vascular disease, and autonomic nerve dysfunction. At present, there are no specific treatments to prevent SAP from progressing to myocardial infarction (MI); instead, supportive care aimed at modulating the systemic inflammatory response remains the standard approach. Despite these efforts, the mortality rate remains high, and existing therapies have limited effectiveness due to the complexity of SAP-induced MI, which is not solely driven by SIRS. Thus, understanding the pathophysiology of cardiac injury in acute pancreatitis and developing targeted molecular therapies are critical for improving outcomes.

## 9. Challenges and perspectives

Pancreatitis remains a challenging condition to treat, despite growing knowledge of its underlying biological processes. Extensive studies have revealed key contributors such as calcium overload, premature enzyme activation, impaired autophagy, mitochondrial dysfunction, endoplasmic reticulum stress, and disordered immune responses including mechanisms like necroptosis and exosome signaling. These insights have expanded our understanding but have not yet translated into widely effective therapies. In particular, cases involving progression from acute to chronic pancreatitis present added complexity due to persistent tissue damage and inflammatory cycles. Current treatment options are largely limited to symptom control and supportive care. Efforts to introduce targeted therapies such as small-molecule inhibitors or anti-inflammatory drugs have shown only modest success. Similarly, TCM provides a rich source of bioactive compounds that act on multiple biological pathways, but issues such as inconsistent formulation, variability in patient response, and lack of standardized evidence continue to limit its integration into mainstream care.

**Scheme 3:**
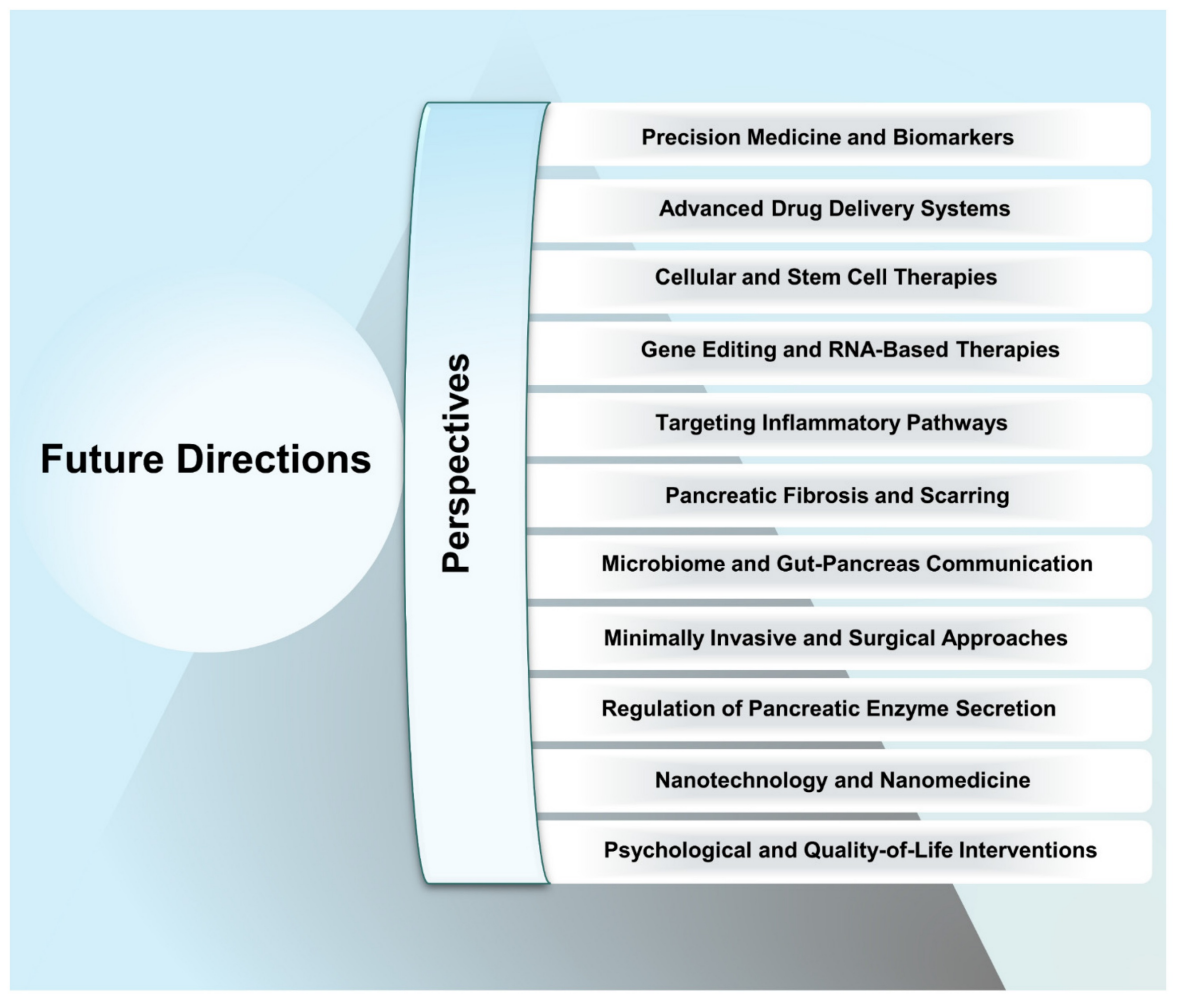
Future directions and perspectives to combat pancreatitis.

Nanomedicine presents a compelling opportunity to address several of these therapeutic gaps. By leveraging nanoscale drug carriers including solid lipid nanoparticles, biodegradable polymers, mesoporous silica, dendrimers, liposomes, micelles, and biologically derived nanocarriers researchers have been able to enhance the delivery of drugs to inflamed pancreatic tissue, control drug release profiles, and improve the overall therapeutic index. Some platforms have also enabled the co-delivery of conventional agents with herbal compounds, providing a multitarget approach that mirrors the complexity of the disease. Yet, despite their potential, the clinical application of nanomedicines faces several challenges: uncertainties around long-term safety, immune responses, scalability of production, and the absence of standardized regulatory pathways. Additionally, many existing animal models fail to fully reflect the human form of pancreatitis, making it difficult to predict therapeutic responses accurately. This limits the reliability of preclinical studies and hinders drug development pipelines (**Scheme 3**).

To enable the successful clinical translation of nanomedicine for pancreatitis treatment, it is essential to address several fundamental challenges. One of the primary hurdles is improving pancreatic-specific targeting, which requires advancing beyond passive ELVIS effects by developing innovative targeting strategies, such as ligand-based or receptor-mediated approaches, to ensure precise nanoparticle delivery to the pancreas while minimizing off-target effects. Additionally, overcoming the blood-pancreas barrier remains a significant challenge, demanding the design of nanoparticles that can effectively penetrate this barrier, possibly through size optimization, surface modification, or advanced delivery mechanisms that facilitate enhanced permeability. Alongside these technological advances, meeting Good Manufacturing Practice (GMP) standards is crucial for ensuring the large-scale production of nanomedicines that are consistent, safe, and compliant with regulatory requirements. Moreover, robust frameworks for long-term toxicological evaluation must be established to assess the safety of nanomedicines over extended periods, addressing concerns about accumulation and potential adverse effects 246,247. By tackling these issues, the clinical viability of nanomedicines for pancreatitis treatment can be significantly enhanced, paving the way for more targeted, effective, and safe therapies.

Moreover, the absence of early diagnostic markers and personalized therapeutic options further contributes to poor clinical outcomes in severe cases. Going forward, innovation in biomaterials and bioengineering paired with insights from systems biology and personalized medicine may offer the tools needed to design more effective, patient-specific treatments. Improved nanocarrier systems capable of responding to disease microenvironments, integrating real-time diagnostics, or delivering combination therapies will likely play a vital role. Multidisciplinary collaboration will be crucial, combining expertise in molecular biology, pharmacology, and materials science to bring these strategies from research to clinical application. Addressing these barriers could shift the treatment paradigm for pancreatitis from symptomatic management toward mechanism-driven, disease-modifying therapies.

### 9.1 Early diagnosis and treatment

Numerous studies have highlighted the pivotal role of oxidative stress in the development of AP. Oxidative stress is closely linked to the progression of AP, arising from an imbalance between oxidative and antioxidative systems within the body. This imbalance is typically driven by an excess production of free radicals, particularly ROS 248. The amount of oxidative stress correlates positively with ROS levels, and the body usually regulates the balance between ROS generation and clearance through various mechanisms to maintain homeostasis. When ROS scavenging mechanisms fail, oxidative stress ensues, disrupting the equilibrium between free radical production and neutralization, which in turn causes cellular damage to proteins, lipids, and DNA. This process accelerates the development of AP. Despite these insights, a straightforward, real-time method for assessing ROS levels to gauge the severity of AP remains absent. Various optical imaging techniques have been explored for *in vivo* ROS detection, such as fluorescence, persistent luminescence, and chemiluminescence (CL). However, most of these techniques require external light sources for activation, and their effectiveness is often hindered by background interference 249.

In response to these challenges, Li *et al*. 250 introduced a novel approach for diagnosing and evaluating the severity of early-stage AP. They developed an integrated semiconducting polymer nanoplatform (SPN) capable of correlating CL signals with ROS concentrations. Their research demonstrated a strong relationship between the intensity of CL imaging and the severity of AP *in vivo*. This study presents a straightforward method utilizing SPN-based CL imaging for assessing the severity of AP. In the future, this nanoplatform could offer a promising diagnostic tool for clinical settings, enabling early detection and severity prediction of AP through CL intensity measurements. This approach could improve treatment outcomes and patient prognosis by facilitating more accurate assessments of disease progression.

### 9.2 Single-cell atlas therapy

Neutrophils, the most abundant leukocytes in peripheral blood, are crucial in defending the body against infections 251. In the context of AP, neutrophils are among the first responders to the site of inflammation, where their activation and persistent infiltration significantly contribute to the inflammatory process and pancreatic damage 252. One key feature of this process is the formation of neutrophil extracellular traps (NETs), which have been shown to exacerbate inflammation in the pancreas and potentially lead to damage in distant organs. Additionally, ROS-induced pancreatic enzymes (PEs) worsen the progression of AP, making the scavenging of ROS a crucial therapeutic target in managing the condition. Moreover, ROS is thought to play a pivotal role in the polarization of neutrophils during inflammation. Recent advancements in nanotechnology, particularly in the development of nanomedicines with multi-enzyme mimicking properties, have opened new avenues for improving AP treatment. These nanomedicines, with their strong ROS-scavenging abilities, hold promise in mitigating oxidative stress in AP. However, the use of broad-spectrum antioxidants at high doses may disrupt the redox balance in normal tissues, potentially causing unintended adverse effects 253.

**Figure 18:**
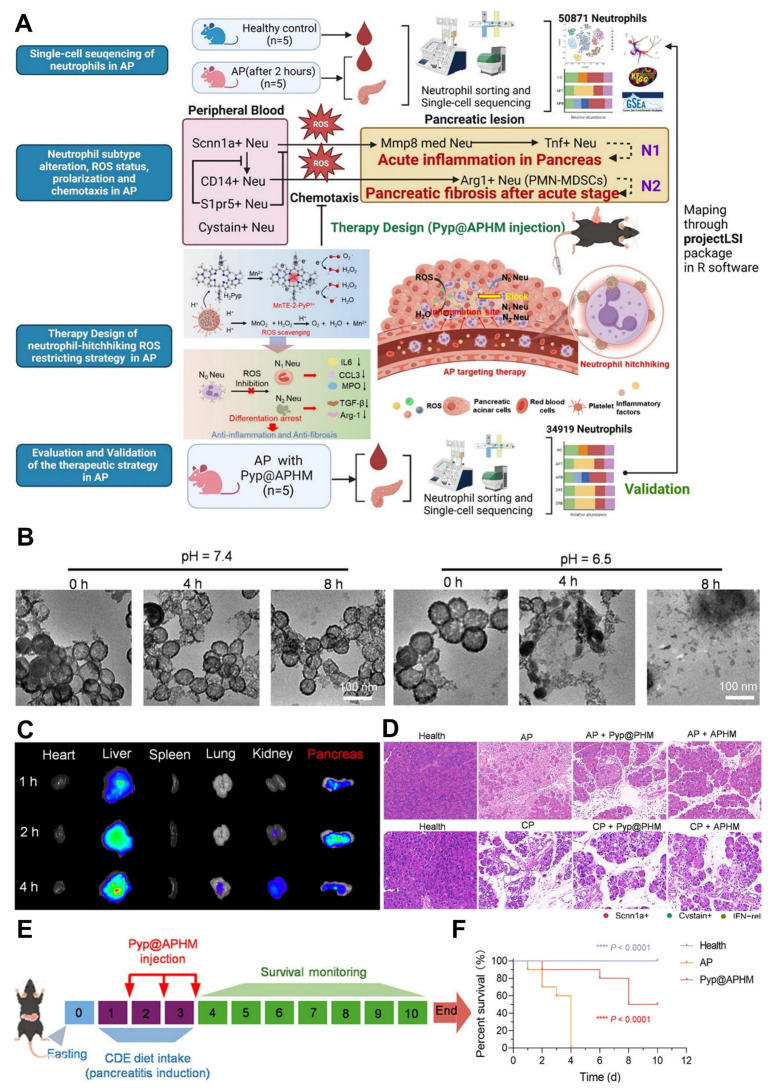
** (A)** A schematic representation of the single-cell sequencing research procedure and the mechanism of Pyp@APHM nanoreactor-mediated hitchhiking treatment for AP. **(B)** Examination of Pyp@APHM's performance both *in vivo* and *ex vivo*. The Pyp@APHM degradation TEM images in neutral PBS (pH 7.4) and moderately acidic PBS (pH 6.5) at various time intervals (0, 4, and 8 hours). **(C)** *Ex vivo* fluorescence images of the heart, liver, spleen, lungs, kidneys, and pancreas showing IR783-labeled Pyp@PHM i) and Pyp@APHM. **(D)** The pathology scores and representative pictures of H&E stains on pancreatic sections (Scale bar:100 μm) from mild AP mice taken 24 hours after therapy. **(E)** The SAP induction and Pyp@APHM therapy trial procedure. **(F)** Mice survival rates during a 10-day period following initial food consumption. Typical digital picture representations. Adapted with permission from ref. 254 Copyrights 2025, Wiley.

In light of these challenges, Zhang *et al*. 254 introduced an innovative approach using a single-cell atlas to map neutrophil alterations and polarization during their migration from peripheral blood to the pancreas in AP. This study employed neutrophil-specific single-cell sequencing to compare the neutrophil populations in the blood and pancreas of AP mice versus healthy controls, identifying key cell subgroups and their potential roles in inflammation (**Figure 18A**). Based on this information, the researchers developed a novel hollow manganese dioxide (HMnO2)-based nanoreactor (Pyp@APHM) that binds specifically to neutrophils via the Ly-6G antibody. This nanoreactor was loaded with porphyrin to modulate neutrophil polarization directly within the inflamed pancreatic tissue. By exploiting the weakly acidic environment characteristic of the inflamed area, Pyp@APHM disintegrates, releasing Mn^2+^ and H_2_TE-2-PyP_4_^+^, which then combine to form MnTE-2-PyP_5_^+^, a compound with catalytic properties similar to natural enzymes like superoxide dismutase (SOD) and catalase (CAT), effectively neutralizing excess ROS, including superoxide anions (O2•^-^) and hydrogen peroxide (H_2_O_2_).

To investigate the nanoreactor's *in vivo* targeting efficiency, Pyp@APHM was dispersed in phosphate-buffered saline (PBS) at pH levels 6.5 and 7.4, mimicking the conditions of AP and healthy pancreatic tissue, respectively. Transmission electron microscopy (TEM) images showed minimal changes in the nanoreactor's structure after 4 hours in neutral PBS (pH 7.4) (**Figure 18B**). *In vivo* targeting efficacy was further validated using an imaging system, showing successful accumulation at the site of inflammation (**Figure 18C**). Histological analysis of pancreatic tissues from AP mice revealed significant damage, including acinar cell necrosis and immune cell infiltration (**Figure 18D**). Interestingly, the Pyp@APHM-treated mice exhibited less severe histopathological changes (**Figure 18E**). The therapeutic potential of Pyp@APHM was also assessed through survival analysis, showing a significant increase in the survival rate of treated mice (50%) after a 10-day observation period, suggesting that this nanoreactor may offer a promising approach for managing SAP (**Figure 18F**).

The recruitment and infiltration of neutrophils in pancreatic tissues play a critical role in AP pathogenesis. Although previous studies have demonstrated that neutrophils, as a uniform population of cells, largely influence AP through ROS production, enzyme secretion, and NET formation, recent research has shown that neutrophils may undergo dynamic reprogramming in response to the microenvironment. This reprogramming results in functional diversity, which influences inflammation, tissue damage, and repair processes. However, comprehensive studies examining the complete transcriptional landscapes of neutrophil phenotypes and their functional roles in AP remain limited.

Neutrophil polarization plays a critical role in the progression of acute pancreatitis (AP), and we have designed a novel nanoreactor that harnesses neutrophils for targeted delivery to inflamed tissues. This innovative approach has shown significant promise in both treating and preventing AP in our experimental models. While the mouse AP model used in our studies does not fully replicate the complex pathophysiological changes seen in human pancreatitis, the "hitchhiking" nanomedicine we developed can specifically target areas of inflammation and facilitate the local production of antioxidant enzyme mimics. This strategy holds potential as a highly effective therapeutic option for clinical management of AP patients. Moreover, Single-cell therapy, along with advancements in early diagnostic tools, presents a novel approach to managing pancreatitis by addressing the disease at the cellular level. This strategy focuses on correcting the underlying biological imbalances, such as oxidative stress, calcium dysregulation, and immune system dysfunction, which are central to pancreatitis pathogenesis. By enabling early detection, these technologies allow for earlier intervention, which can prevent the progression of the disease and minimize the risk of complications like pancreatic necrosis or fibrosis. Moreover, personalized treatment plans derived from single-cell analysis can target specific cellular mechanisms, improving the precision and effectiveness of therapies while reducing the likelihood of adverse effects 255. This shift toward tailored, cellular-level treatments has the potential to significantly improve both short-term recovery and long-term disease management, offering a more effective, efficient solution for pancreatitis care.

## 10. Conclusion

The management of pancreatitis is evolving toward a more integrated, targeted approach, with a strong focus on controlling inflammation and immune dysfunction at the heart of current therapeutic strategies. Anti-inflammatory treatments have become central to modern interventions, with the goal of addressing both acute and chronic phases of the disease. One of the most promising avenues is the use of nanomedicine, which includes synthetic and biomimetic nanocarriers that improve the delivery and absorption of anti-inflammatory agents. These nanocarrier systems provide a means to precisely target pancreatic tissue while minimizing off-target effects, offering enhanced therapeutic outcomes in reducing inflammation and mitigating long-term damage from pancreatitis. Beyond nanotechnology, other innovative therapies, such as exosome-based interventions, gas therapies (like hydrogen and hydrogen sulfide), gene therapy, specialty food, living probiotics therapy and the use of single cell therapy and early diagnosis as perspective therapies are gaining traction in the treatment of pancreatitis. Exosomes, with their ability to modulate immune responses and facilitate tissue repair, represent a novel approach to managing the inflammatory processes central to disease progression. Similarly, gas-based therapies have been shown to alleviate oxidative stress, which is crucial for controlling inflammation, while probiotics are explored for their potential to restore balance to the gut microbiome and reduce systemic inflammation.

Traditional therapeutic options, including traditional Chinese medicine, are also being integrated into contemporary treatment strategies, working synergistically with newer modalities to improve patient outcomes. The convergence of these treatments, alongside advancements in gene therapy and multitargeted interventions, enables a comprehensive approach to managing both the immediate inflammatory response and the long-term consequences, such as fibrosis, that arise from chronic pancreatitis. Ultimately, the future of pancreatitis treatment lies in personalized, precision medicine approaches and perspective therapies like single cell therapy and early diagnosis that combine the latest in nanotechnology, immune modulation, and advanced therapeutic strategies. These innovations will not only improve disease management but also allow clinicians to offer more individualized care, potentially transforming pancreatitis from a chronic, often debilitating condition into a manageable illness with a better quality of life for patients. As research in these fields continues to expand, the next generation of therapies promises to address the underlying mechanisms of the disease more effectively, providing improved outcomes for those affected by pancreatitis.

## Figures and Tables

**Table 1:** Summary of all types of mechanisms involved in treatment of pancreatitis.

Mechanism Type	Specific Pathway	Target	Importance	Examples	References
Anti-inflammatory	NF-κB Signaling Inhibition	Reduces cytokines (TNF-α, IL-6) and immune activation	Prevents inflammation and tissue damage	Inflixima**,** Tocilizua**,** Curcumin	43
	Cytokine Modulation	Suppresses TNF-α, IL-6, IL-1β release	Reduces inflammati on, prevents immune storm	Adalimub, Anakinra, Canakinab	44
	JAK-STAT Inhibition	Blocks cytokine signaling, reduces inflammation	Limits cytokine-driven inflammation	Tofacitin, Ruxolitinb	45
	NLRP3 Inflammasome Inhibition	Decreases IL-1β, IL-18 secretion	Prevents inflammasome-driven injury	MCC950, VX-765	46
	Pro-resolving Mediators	Promotes inflammation resolution and tissue repair	Enhances repair, reduces chronic inflammation	Resolvins, Protectins	47
Oxidative stress	Antioxidant Activation	Scavenges ROS, reduces oxidative damage	Prevents pancreatic cell injury	N-acetylcysteine, Sulforaphane, Vitamin C	48
Protease inhibition	Serine Protease Inhibition	Prevents trypsin activation, reduces autodigestion	Prevents premature enzyme activation and pancreatic damage	Gabexate, Nafamostat	49
	Cysteine Protease Inhibition	Inhibits cathepsins, prevents trypsinogen activation	Reduces enzyme-induced pancreatic injury	E64, Cathepsin inhibitors	50
Pain modulation	CGRP Antagonism	Reduces neurogenic pain and inflammation	Alleviates pain and reduces inflammation	Telcagepant, CGRP antagonists	51
	Opioid Receptor Modulation	Reduces opioid-induced inflammation and pain	Modulates pain response, reduces opioid side effects	Morphine, Gabapentin	52
Immunomodulation	Treg Induction	Expands Tregs, suppresses immune response	Reduces inflammation, prevents fibrosis	IL-2, Rapamycin	53
	M2 Macrophage Polarization	Promotes M2 macrophages, resolves inflammation	Enhances tissue repair and inflammation resolution	IL-4, PPAR-γ agonists	54
Fibrosis modulation	Hedgehog Inhibition	Reduces fibrosis, limits collagen deposition	Prevents fibrotic tissue formation	Vismodegib, GDC-0449	55
	TGF-β Inhibition	Reduces fibrosis, prevents collagen synthesis	Prevents fibrosis, preserves pancreatic function	Fresolimumab, TGF-β monoclonals	56

**Table 2:** Chronic complications of pancreatitis, the inflammatory pathways involved, and potential anti-inflammatory targets.

Comorbidity	Common Mechanisms	Impact on Pancreatitis	Impact on Comorbidity
Pancreatitis + Diabetes	Inflammation, Oxidative stress, Insulin resistance	Worsens β-cell dysfunction, Impaired insulin secretion	Poor blood glucose control, Increased diabetes complications
Pancreatitis + cardiovascular disease	Inflammation, Endothelial dysfunction, Oxidative stress	Impaired pancreatic blood flow, Increased thrombosis risk	Accelerates atherosclerosis- Higher cardiovascular risk
Pancreatitis + Liver Disease	Inflammation, Oxidative stress, Bile acid dysregulation	Liver dysfunction impacts detoxification, Bile flow disruption	Liver fibrosis progression, Reduced detox capacity
Pancreatitis + renal disease	Hypoperfusion, Inflammation, Oxidative stress	Risk of acute kidney injury (AKI), Fluid imbalance	AKI progression to CKD, Electrolyte imbalances
Pancreatitis + Obesity	Chronic inflammation, Insulin resistance, Dysbiosis	Increased severity of pancreatitis, Worsens pancreatic fibrosis	Worse metabolic dysfunction- Increased risk of diabetes
